# Sex-Specific Effects of Nutritional Supplements for Infants Born Early or Small: An Individual Participant Data Meta-Analysis (ESSENCE IPD-MA) II: Growth

**DOI:** 10.3390/nu14020392

**Published:** 2022-01-17

**Authors:** Luling Lin, Greg D. Gamble, Caroline A. Crowther, Frank H. Bloomfield, Massimo Agosti, Stephanie A. Atkinson, Augusto Biasini, Nicholas D. Embleton, Fernando Lamy Filho, Christoph Fusch, Maria L. Gianni, Hayriye Gözde Kanmaz Kutman, Winston Koo, Ita Litmanovitz, Colin Morgan, Kanya Mukhopadhyay, Erica Neri, Jean-Charles Picaud, Niels Rochow, Paola Roggero, Kenneth Stroemmen, Maw J. Tan, Francesco M. Tandoi, Claire L. Wood, Gitte Zachariassen, Jane E. Harding

**Affiliations:** 1 Liggins Institute, University of Auckland, Auckland 1023, New Zealand; luling.lin@auckland.ac.nz (L.L.); gd.gamble@auckland.ac.nz (G.D.G.); c.crowther@auckland.ac.nz (C.A.C.); f.bloomfield@auckland.ac.nz (F.H.B.); 2 NICU, Woman and Child Department, Ospedale Del Ponte, Insubria University, 21100 Varese, Italy; massimo.agosti@asst-settelaghi.it (M.A.); Francesco.Tandoi@asst-settelaghi.it (F.M.T.); 3 Department of Pediatrics, Faculty of Health Sciences, McMaster University, Hamilton, ON L8S 4L8, Canada; satkins@mcmaster.ca (S.A.A.); Christoph.Fusch@klinikum-nuernberg.de (C.F.); nielsrochow@gmail.com (N.R.); 4 Donor Human Milk Bank Italian Association (AIBLUD), 20126 Milan, Italy; augustoclimb@gmail.com; 5 Population Health Sciences Institute, Newcastle University, Newcastle upon Tyne NE2 4AX, UK; nicholas.embleton@newcastle.ac.uk; 6 Departamento de Medicina, Universidade Federal do Maranhão (UFMA), São Luís 65080-805, Brazil; lamyfilho@gmail.com; 7 Department of Pediatrics, Nuremberg General Hospital, Paracelsus Medical University, 90471 Nuremberg, Germany; 8 Fondazione IRCCS Cà Granda Ospedale Maggiore Policlinico, Via Commenda 12, 20122 Milan, Italy; maria.gianni@unimi.it (M.L.G.); paola.roggero@unimi.it (P.R.); 9 Department of Clinical Sciences and Community Health, University of Milan, Via Commenda 19, 20122 Milan, Italy; 10 Department of Neonatology, Bilkent City Hospital, Ankara 6800, Turkey; gzdekanmaz@yahoo.com; 11 Department of Nutrition and Food Science, Wayne State University, Detroit, MI 48202, USA; aa3796@wayne.edu; 12 Department of Neonatology, Meir Medical Center, Kfar Saba 4428164, Israel; litmani@clalit.org.il; 13 Department of Neonatology, Liverpool Women’s Hospital, Liverpool L8 7SS, UK; colin.morgan@lwh.nhs.uk; 14 Department of Pediatrics, Post Graduate Institute of Medical Education and Research (PGIMER), Chandigarh 160012, India; kanyapgi@gmail.com; 15 Department of Psychology, University of Bologna, 40126 Bologna, Italy; erica.neri4@unibo.it; 16 Division of Neonatology, Hôpital de la Croix-Rousse, Hospices Civils de Lyon, 69004 Lyon, France; jean-charles.picaud@chu-lyon.fr; 17 CarMen Laboratory, INSERM, INRA, Claude Bernard University Lyon 1, 69310 Pierre Benite, France; 18 Department of Neonatal Intensive Care, Division of Paediatric and Adolescent Medicine, Rikshospitalet, Oslo University Hospital, 0188 Oslo, Norway; kestromm@gmail.com; 19 Department of Developmental Paediatrics, Alder Hey Children’s NHS Foundation Trust, Liverpool L12 2AP, UK; tanyeo001@aol.com; 20 Translational and Clinical Research Institute, Faculty of Medical Sciences, Newcastle University, Newcastle upon Tyne NE2 4AX, UK; claire.wood@newcastle.ac.uk; 21 H.C. Andersen Children’s Hospital, Odense University Hospital and University of Southern Denmark, 5000 Odense, Denmark; gitte.zachariassen@rsyd.dk

**Keywords:** macronutrient supplementation, preterm infants, small-for-gestational-age infants, growth, individual participants data meta-analysis, systematic review

## Abstract

Neonatal nutritional supplements may improve early growth for infants born small, but effects on long-term growth are unclear and may differ by sex. We assessed the effects of early macronutrient supplements on later growth. We searched databases and clinical trials registers from inception to April 2019. Participant-level data from randomised trials were included if the intention was to increase macronutrient intake to improve growth or development of infants born preterm or small-for-gestational-age. Co-primary outcomes were cognitive impairment and metabolic risk. Supplementation did not alter BMI in childhood (kg/m^2^: adjusted mean difference (aMD) −0.11[95% CI −0.47, 0.25], *p* = 0.54; 3 trials, *n* = 333). Supplementation increased length (cm: aMD 0.37[0.01, 0.72], *p* = 0.04; 18 trials, *n* = 2008) and bone mineral content (g: aMD 10.22[0.52, 19.92], *p* = 0.04; 6 trials, *n* = 313) in infancy, but not at older ages. There were no differences between supplemented and unsupplemented groups for other outcomes. In subgroup analysis, supplementation increased the height z-score in male toddlers (aMD 0.20[0.02, 0.37], *p* = 0.03; 10 trials, *n* = 595) but not in females, and no significant sex interaction was observed (*p* = 0.21). Macronutrient supplementation for infants born small may not alter BMI in childhood. Supplementation increased growth in infancy, but these effects did not persist in later life. The effects did not differ between boys and girls.

## 1. Introduction

The risk of poor growth, slow development and disability are increased in preterm and small-for-gestational-age (SGA) infants [1,2,3,4]. Providing preterm and SGA infants with more protein and energy during the first few weeks after birth may improve short-term growth and result in better developmental outcomes from infancy to adolescence [5,6,7,8,9,10]. However, in observational studies, rapid Body Mass Index (BMI) gain and linear growth in infancy are associated with better cognitive development, but at the expense of increased risk for adiposity, metabolic and cardiovascular disease in adulthood [11]. Further, bone mineral content is often lower in preterm than term infants and the rate of metabolic bone disease is inversely proportional to birthweight and gestational age [12,13,14]. In turn, metabolic bone disease during infancy may result in neonatal rickets, childhood fractures, and impaired growth [14].

Nutritional supplementation is widespread as standard practice for infants born preterm or SGA [8], but there has been limited evidence regarding potential long-term consequences. Further, different prenatal growth patterns between girls and boys may potentially result in sex-specific responses to nutritional supplements [15]. Previous randomised trials have found sex-specific effects of early macronutrient supplements [6,16], and a recent systematic review found enhanced nutrition may improve length in toddler boys but not girls [17]. However, although thousands of infants have participated in randomised trials of enhanced nutrition, few trials have analysed girls and boys separately, and the long-term and the sex-specific effects of supplementation remain unclear.

Therefore, we undertook an individual participant data (IPD) meta-analysis (MA) of trials reporting long-term outcomes after macronutrient supplements for infants born preterm or SGA.

## 2. Methods

The protocol of the ESSENCE (Sex-Specific Effects of Early Nutritional Supplements in Children Born Early or Small) IPD-MA was published [18]. The study followed the IPD-MA approach [19] registered prospectively in PROSPERO (CRD42017072683) and reported according to the Preferred Reporting Items for Systematic Reviews and Meta-Analyses (PRISMA) guidelines (Text S1).

### 2.1. Search Strategies

We searched Ovid MEDLINE, Embase, Cochrane Library Central Registry of Controlled Trials, and Cochrane Database of Systematic Reviews from inception to 1 April 2019. We also searched for eligible ongoing trials in Current Controlled Trials (www.controlled-trials.com), Clinical Trials (www.clinicaltrials.gov), the Australian and New Zealand Clinical Trials Registry (www.anzctr.org.au), and WHO ICTRP Search Portal (https://apps.who.int/trialsearch/) on 30 January 2021 (Search Table S10).

### 2.2. Criteria for Inclusion and Exclusion

We included published and unpublished randomised and quasi-randomised trials without restrictions on the date of publication or language. Eligible trials studied infants born preterm (<37 weeks’ gestation) or born SGA (birthweight <10th centile for gestational age), and the intervention was intended to increase the intake of one or more macronutrients (protein, carbohydrate, fat, energy content, or protein to energy ratio), with the primary aim of improving growth and development.

Interventions could be enteral or parenteral or a combination, commence at any time during the hospitalisation or after discharge from hospital, and must have been provided for a minimum duration of one week. Trials were eligible if they reported comparisons between unsupplemented nutrition and supplemented nutrition with parenteral supplements, human breast milk supplements, formula milk, or other macronutrients. Trials were excluded that examined the timing of the introduction of nutrition (early versus delayed feeding); that compared macronutrients of different composition (e.g., different types of lipids or proteins); whose outcomes focussed on gastrointestinal development rather than growth and development; and reporting on variations in the composition of micronutrients (including sodium, potassium, calcium, phosphorus, vitamins, other minerals, amino acids, fatty acids).

Outcome data required reporting beyond term equivalent age (>37 weeks’ postmenstrual age) or following discharge from hospital after birth. Outcomes were categorised and evaluated in infancy (≤1 years), toddlers (1 to 3 years), childhood (4 to 8 years), adolescence (9 to 18 years), and adulthood (>18 years).

The primary outcome was BMI in childhood (4 to 8 years).

Secondary outcomes were (1) Growth assessments: weight (raw data and z scores), length/height (raw data and z scores), head circumference (raw data and z scores), Ponderal Index, BMI, body composition (fat mass, fat-free mass, measured by bioimpedance or (Dual-energy X-ray absorptiometry) DXA, skinfold thickness, or other methods); (2) Bone development: bone mineral content, volumetric bone mineral density, bone fractures; (3) Nutrition: feeding tolerance; intake (protein, energy); appetite; breastfeeding and duration.

### 2.3. Quality Assessment

The methods specified in the Cochrane Handbook for Systematic Reviews of Interventions [20] were used to evaluate the quality of eligible trials: (1) random sequence generation (selection bias); (2) allocation concealment (selection bias); (3) blinding of participants, personnel, and outcome assessment (performance and detection bias); (4) incomplete outcome data (attrition bias); (5) selective reporting (reporting bias); (6) Other bias (checking for bias due to problems not covered by (1) to (5) above).

### 2.4. Data Synthesis and Statistical Analysis

Trialists provided de-identified data, which were recoded as required, verified, and checked for consistency with published data. Each final trial dataset was verified by the original trialists before analysis individually using IPD-MA pre-specified variables and outcomes, and the results were returned to the trialists for verification. The individual trial datasets were then combined for IPD meta-analysis. There was no imputation for missing data.

We used a one-stage approach to analyse each outcome so that the IPD from all eligible trials were included in a single random effects model. Binary outcomes were analysed using log-binomial regression models, and data were reported as Relative Risk (RR) with 95% CIs and associated 2-sided *p*-values. We changed the algorithm to encourage the models to converge where specified. Continuous data were analysed using linear regression models, and data were reported as mean differences (MD) with 95% CIs and associated 2-sided *p*-values. All models included adjustment for pre-specified confounders. The analyses of IPD were adjusted for sex, gestational age, and birth weight z-scores.

We explored the effects of infant sex by presenting data separately for each sex as pre-specified subgroups and by testing a treatment by identifying sex interaction terms within the model. Statistical analyses were performed using SAS (v.9.4, SAS Institute, Cary, NC, USA). We validated the one-stage model using a two-stage approach in RevMan 5.3 [21].

### 2.5. Planned Subgroup and Sensitivity Analyses

Where data were available, subgroup analyses were performed to explore whether the effect of supplements differed between subgroups with regard to sex, size of infant at birth (≤1 kg vs. >1 kg at birth), size for gestation at birth (≤10th centile vs. >10th centile), gestational age (≤28 completed weeks vs. 29 to 32 completed weeks vs. 33 to 36 completed weeks), timing of supplementation (in hospital vs. after discharge vs. both), type of supplement (protein vs. carbohydrate vs. fat vs. multicomponent and their interactions), primary feed (breastmilk vs. formula vs. parenteral combined with enteral feed), trial timing (conducted before or after 2000), and duration of supplement (1 to 2 weeks vs. 3 to 6 weeks vs. more than 7 weeks). We also tested for interactions between effects. No unplanned analyses were performed.

Sensitivity analyses were performed to assess whether the results were robust enough for trial design by excluding trials assessed as high risk of bias. Where trials were unable to contribute IPD, we assessed the robustness and exclusion of these trials by combining their aggregate data (AD) with the IPD. The analyses of combined IPD and AD were adjusted for gestational age.

## 3. Results

### 3.1. Search Results

**Figure 1:**
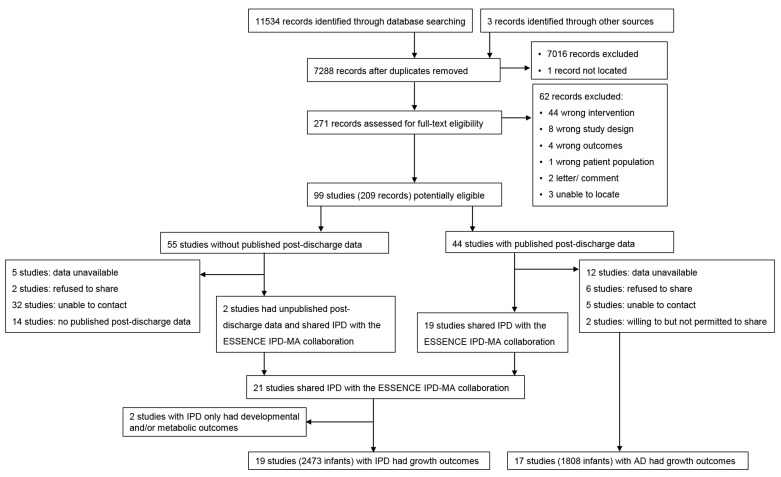
Flow diagram of included studies.

We identified 7288 records after de-duplication. Title and abstract screening left 271 records for full-text screening. We excluded 62 records that did not meet the inclusion criteria (Table S11). The remaining 99 potentially eligible trials (209 records) were included, among which 44 trials had published post-discharge data, and 55 trials did not. We attempted to contact the authors of all 99 trials. The authors of 21 trials agreed to share their IPD. Of these, 19 trials with 2473 infants had post-discharge growth outcome data, and an additional 17 trials with 1808 infants for which IPD data were not available had published growth outcomes (Figure 1).

Details of the studies are described in Table 1.

Included studies were of variable methodological quality (Table S1). Two studies [22,23] with IPD available were at high risk of selection bias because infants were quasi-randomised by birth date. We considered 32% of included trials at high risk of performance bias because of lack of blinding. For many studies, blinding was not possible, e.g., when the intervention was breastfeeding. Attrition bias was assessed for BMI (primary outcome). One study [24] was at high risk of attrition bias due to an imbalance in baseline characteristics between supplemented and unsupplemented groups in those who were followed up.

### 3.2. Primary Outcome-BMI in Childhood

**Figure 2:**
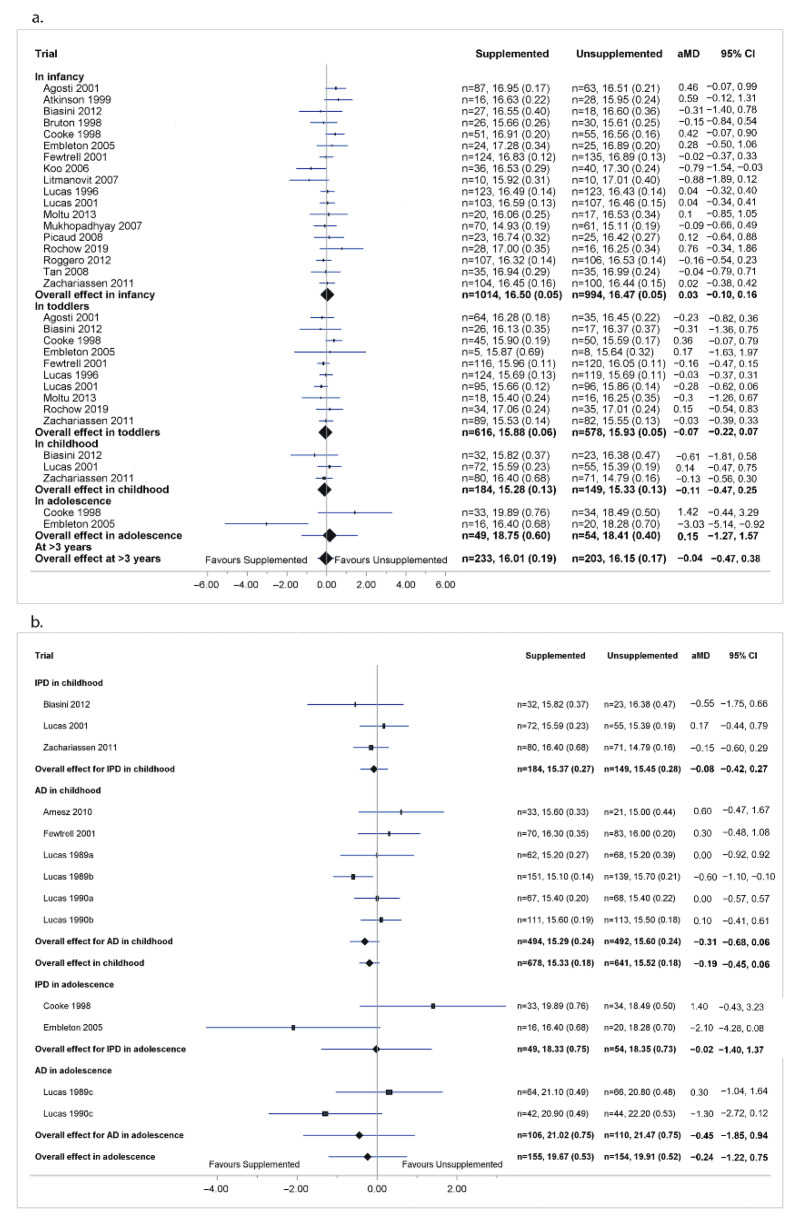
Effect of macronutrient supplements on BMI. (**a**) IPD analysis of BMI. (**b**) Combined IPD and AD analysis of BMI in childhood and adolescence. The box size of point estimate is proportional to inverse variance data. For IPD analysis, *p*-value for heterogeneity in infancy = 0.43, in toddlers = 0.78, childhood = 0.39, in adolescence = 0.01, at >3 years = 0.006. Numbers in bold are overall effects.

In childhood, there was no significant difference in BMI between supplemented and unsupplemented groups in the analysis of IPD (kg/m^2^: aMD −0.11[95% CI −0.47, 0.25], *p* = 0.54; 3 trials, 333 children) or of combined IPD and AD (kg/m^2^: aMD −0.19[−0.45, 0.06], *p* = 0.14; 9 trials, 1319 children) (Figure 2).

### 3.3. Secondary Outcomes

There was no significant difference between supplemented and unsupplemented groups in BMI in the analysis of IPD or of combined IPD and AD (Figure 2 and Figure S1), or in BMI z-scores in the analysis of IPD alone, in infancy, toddlers, childhood, adolescence, or at >3 years (Figure S2).

There was no significant difference between supplemented and unsupplemented groups in BMI in the analysis of IPD or of combined IPD and AD (Figure 2 and Figure S1), or in BMI z-scores in the analysis of IPD alone, in infancy, toddlers, childhood, adolescence, or at >3 years (Figure S2).

In infancy, there was no significant difference in weight between supplemented and unsupplemented groups in the analysis of IPD (Figure S3). However, supplementation increased weight in infancy in the analysis of combined IPD and AD (Figure S4). There was no significant effect of supplementation on weight in the analysis of IPD or combined IPD and AD in toddlers, childhood, adolescence, or at >3 years (Figures S3 and S4), or on weight z-scores in infancy, toddlers, or childhood (Figure S5). Weight-for-age reference data are not available beyond age 10.

In infancy, supplementation increased length in the analysis of IPD (Figure S6) and of combined IPD and AD (Figure S7a). However, supplementation had no significant effect on height in the analysis of IPD or combined IPD and AD in toddlers, childhood, adolescence, or at >3 years (Figures S6 and S7), or on height z-scores in any age group (Figure S8). There was also no difference in weight for length z-scores in the analysis of IPD in infancy or toddlers (Figure S9).

In infancy, there was no significant difference in head circumference between supplemented and unsupplemented groups in the analysis of IPD (Figure S10), but in the combined IPD and AD analysis, supplementation increased head circumference (Figure S11). In the analysis of IPD and of combined IPD and AD, supplementation did not alter head circumference in toddlers or childhood (Figures S10 and S11) or head circumference z-scores in infancy or toddlers (Figure S12).

There was no significant difference between supplemented and unsupplemented groups in fat mass or fat mass index in the analysis of IPD or of combined IPD and AD in infancy, childhood, adolescence, and at >3 years (Figures S13 and S14), or in percent fat mass in the analysis of IPD in infancy, childhood, adolescence, and at >3 years or of combined IPD and AD in infancy (Figure S15). Similarly, there was no effect on lean mass or lean mass index in the analysis of IPD or of combined IPD and AD in infancy, childhood, adolescence, or at >3 years (Figures S16 and S17).

In infancy, supplementation increased bone mineral content (BMC) in the analysis of IPD and of combined IPD and AD. In childhood, there was no IPD for BMC, but no significant effect of supplementation on BMC in the analysis of AD. There was also no significant effect of supplementation on BMC in the analysis of IPD or combined IPD and AD in adolescence or at >3 years (Figure S18).

In infancy, there was no significant effect of supplementation on BMD (bone mineral density) in the analysis of IPD, but supplementation increased BMD in the analysis of combined IPD and AD. In childhood, there was no IPD for BMD, and no significant effect of supplementation on BMD in the analysis of AD. There was also no significant effect of supplementation on BMD in the analysis of IPD or of combined IPD and AD in adolescence or at >3 years (Figure S19).

There were no data for any outcomes >18 years.

**Figure 3:**
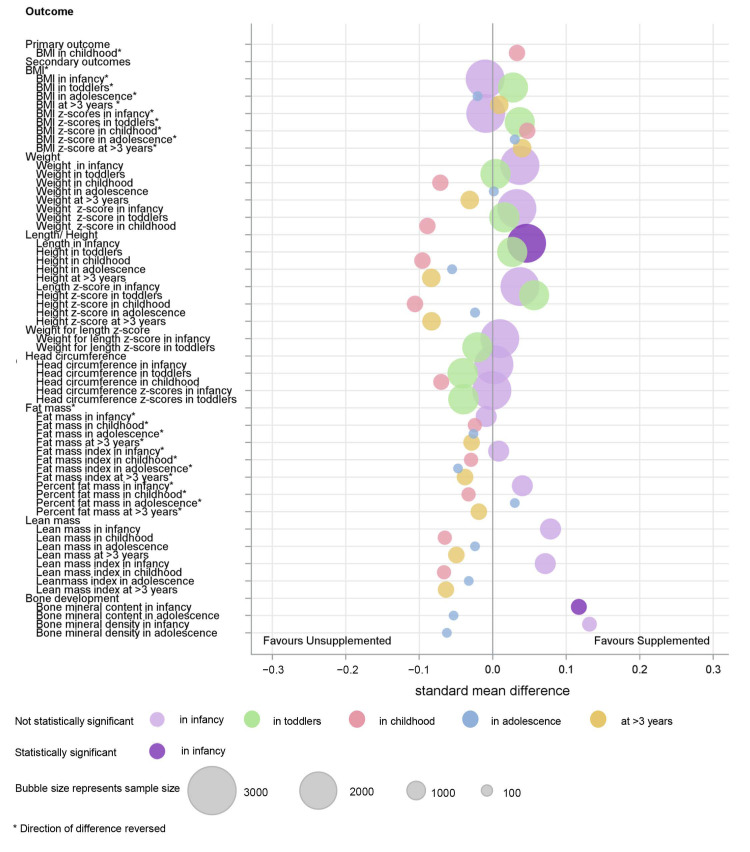
Summary of IPD analysis of macronutrient supplements on growth outcomes.

**Figure 4:**
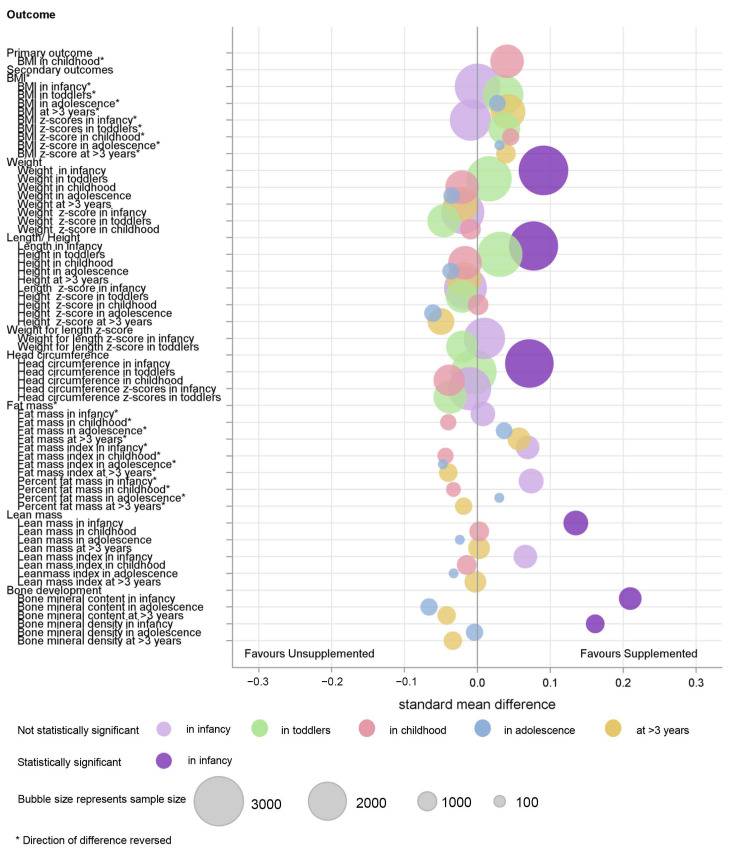
Summary of combined IPD and AD analysis of macronutrient supplements on growth outcomes.

An overview of the main findings of the meta-analyses are provided graphically (Figure 3 and Figure 4).

### 3.4. Subgroup Analysis

#### 3.4.1. Sex of Infant

**Figure 5:**
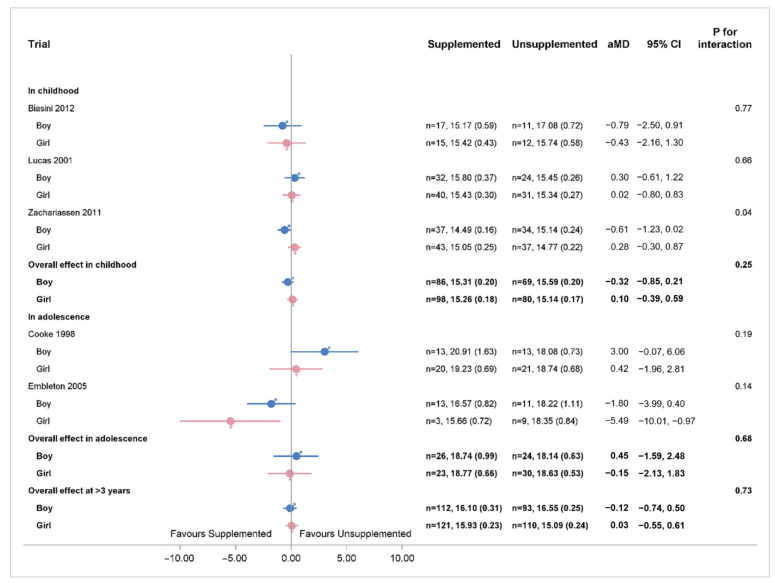
IPD analysis of BMI separated for boys and girls. *p*-value for heterogeneity for boys in childhood = 0.17. *p*-value for heterogeneity for girls in childhood = 0.74. Numbers in bold are overall effects.

In childhood, there was no significant difference in BMI between supplemented and unsupplemented groups for boys or girls (Figure 5).

In toddlers, supplemented boys had higher height z-scores than unsupplemented boys, but there were no effects of supplementation for girls and no significant interaction between effects (Table S2).

#### 3.4.2. Size for Gestation of the Infant

The effects of supplementation on BMI in childhood were not different in SGA and AGA subgroups (Table S3). However, there was a significant interaction between size for gestation and the effects of supplements on height z-scores in childhood, with supplementation decreasing height z-scores in SGA but not AGA children. Supplementation also decreased weight z-scores in childhood and height z-scores at >3 years for children born SGA, but increased height z-scores in toddlers and increased lean mass, lean mass index, and BMC in infancy for children born AGA (no significant interaction).

#### 3.4.3. Size of Infant at Birth

The effects of supplements on BMI in childhood were not different in those with birthweight ≤1 kg and >1 kg (Table S4). Supplemented children with birthweight >1 kg had greater height z-scores as toddlers than unsupplemented children, whereas supplemented children with birthweight ≤1 kg were shorter in childhood and presented lower height z-scores at >3 years, but none of the interactions were significant.

#### 3.4.4. Gestational Age of Infant at Birth

There were significant interactions between supplementation and different gestational age subgroups for BMI and BMI z-scores in childhood and in toddlers and for weight for length z-scores in toddlers (Table S5). Supplemented children born very preterm (29 to 32 weeks) had lower BMI and BMI z-scores as toddlers and in childhood than unsupplemented children, but those born extremely or moderate to late preterm did not. Supplementation decreased weight z-scores and height z-scores in childhood for children moderate to late preterm and decreased weight z-scores in childhood and weight for length z-scores in toddlers for children born very preterm, but had no effect on weight and height z-scores for children born extremely preterm (no significant interaction terms).

#### 3.4.5. Timing of Supplement

Supplementation did not alter BMI in childhood in the subgroups who received supplements in hospital or post-discharge (Table S6). However, children who received supplements in hospital, but not those who received supplements post-discharge, had lower BMI, BMI z-scores in adolescence and at >3 years, and lower weight, fat mass, fat mass index, lean mass index at >3 years, and percent fat mass in adolescence. Children who had received supplements post-discharge, but not those who received supplements in-hospital, had increased weight and length in infancy and height z-scores in toddlers (all interaction terms *p* < 0.05).

#### 3.4.6. Type of Supplement

Supplementation did not alter BMI in childhood in the subgroups who received protein and multicomponent fortification (Table S7). However, supplemented children who received additional protein, but not those who received multicomponent fortification, had lower BMI, BMI z-scores in adolescence and at >3 years, and lower weight, fat mass, fat mass index, lean mass index at >3 years, and percent fat mass in adolescence (all interaction terms *p* < 0.05). Supplemented children who received multicomponent fortification, but not those who received protein fortification, had increased length in infancy and height z-scores in toddlers, but there were no significant interactions.

#### 3.4.7. Primary Feed

There were no differences in BMI in childhood between supplemented and unsupplemented groups for children whose primary feed was breastmilk, formula, or parenteral and enteral feeds (Table S8). However, there were significant interactions between supplements and different primary feed for height and height z-scores in toddlers. Supplementation increased height and height z-scores only for toddlers who received formula as primary feed, but not for those who received breast milk or parenteral and enteral as the primary feed (interaction terms *p* < 0.05). For children receiving formula as primary feed, supplementation also increased length and bone mineral content in infancy; for children receiving breast milk as primary feed, supplementation decreased head circumference and head circumference z-scores in toddlers, but these interaction terms were not significant. These effects were not due to differences between the primary feed groups in baseline macronutrient intakes or the quantity of the supplements (Text S2).

#### 3.4.8. Different Trial Timing

There were no differences in BMI in childhood between supplemented and unsupplemented groups in the subgroups of trials conducted before or after 2000 (Table S9). In trials conducted after 2000, but not those conducted before, supplemented children had lower BMI and BMI z-scores than unsupplemented children in adolescence and at >3 years (all interaction terms *p* < 0.05). In trials conducted before 2000, supplementation increased weight, weight z-scores, length, head circumference, fat mass in infancy, fat mass index at >3 years, and BMC in infancy (all interaction terms *p* < 0.05); supplemented children also had increased lean mass and BMD in infancy, but these interaction terms were not significant. These effects were not due to changes over time in baseline macronutrient intake or quantity of the supplements (Text S2).

## 4. Discussion

In this meta-analysis, we found no evidence that early macronutrient supplements had any effect on BMI in childhood. Early macronutrient supplementation did slightly increase growth in infancy, but none of these effects persisted into later life, and there were no significant sex differences.

Observational studies have reported that faster weight gain in early life is associated with higher fat mass in later life [25,26,27]. We used BMI in childhood as the primary outcome for this analysis, as it is the most widely available measure of adiposity and a predictor for overweight and obesity in adulthood [28,29]. We found that early supplements did not alter BMI or fat mass from infancy through to adolescence. Large numbers of infants were included in these analyses, and confidence intervals were narrow for infancy, although wider for >3 years. This is consistent with our previous systematic review [17] but contrasts with observational studies. Our findings suggest that early macronutrient supplements are very unlikely to increase later adiposity or risk of obesity.

In the IPD-MA, supplementation slightly increased length in infancy despite significant heterogeneity but had no effect on weight, head circumference, or lean mass in infancy. In the sensitivity analysis, which combined IPD and AD from around 800 additional children, supplementation increased weight, length, head circumference, and lean mass in infancy, but the size of these effects was small. Furthermore, in the analyses of both IPD and combined IPD and AD, weight, length, and head circumference z-scores were not altered by supplementation. The discrepancy between findings using absolute values and those using z-scores may be because of the different numbers of trials; 13–15 trials with AD reported absolute values, while only 2 reported z-scores. Anthropometric measurements may have different distributions for different populations, so z-scores are likely to provide a more reliable effect estimate. However, the findings of the effects of supplements on absolute measurements were based on large numbers of children and had narrow confidence intervals. We, therefore, conclude that supplementation does increase growth in infancy, but effects are small and do not persist after the first year. This contrasts with the findings of the systematic review of published randomized trials [17] that showed supplementation increased growth in toddlers. However, in this IPD-MA, we were able to separate infancy (≤1 year) and toddlers (1 to 3 years), whereas these age groups were combined in the previous review.

In the analysis of IPD and combined IPD and AD, we found that supplementation increased bone mineralisation in infants, suggesting that early macronutrient supplements for preterm and SGA infants can help compensate for the limited opportunity for accretion of bone minerals during the last trimester of pregnancy [6,7,8]. A deficit in mineralisation can increase the risk of childhood fractures and reduce peak bone mass, and potentially increase the risk of later osteoporosis [30,31]. Thus, nutritional supplements to achieve adequate early bone accretion may be of initial clinical benefit for infants born preterm or SGA, although these effects did not persist into later life.

Different growth patterns between girls and boys before birth may potentially determine sex-specific responses to early environmental perturbations, including nutrition [15]. As is well known, preterm boys are at greater risk of adverse health outcomes than preterm girls [32]. Sex-specific effects have also been reported in animal studies. For example, in sheep, prenatal testosterone treatment reduced the body weight and height of newborn sheep from both sexes, but only females exhibited catch-up growth during 2–4 months of postnatal life [33]. Therefore, we hypothesised that the effect of early macronutrient supplements might differ between girls and boys. However, we did not find any sex-specific effects of supplements on BMI in childhood. Supplementation did increase height z-scores in toddler boys but not girls, but the interaction terms were not statistically significant. Thus there is no evidence of widespread sex-specific effects of nutritional supplements in preterm and SGA infants, and therefore no evidence that neonatal nutritional supplements should differ for girls and boys.

In the subgroup analysis of children born SGA versus AGA, supplemented children born AGA had increased lean mass and lean mass index in infancy, although this did not persist in later life. However, supplemented children born SGA had lower weight z-scores, height z-scores in childhood, and height at >3 years. After birth, the generally accepted goal is to provide enough nutrients for children born SGA to achieve adequate postnatal growth [34]. However, our limited data suggest that early macronutrient supplementation may only benefit children born AGA, and may even result in impaired growth for children born SGA.

We also found that supplementation increased height z-scores for toddlers born >1 kg and decreased height in childhood and height z-scores at >3 years for children born ≤1 kg, although the interaction terms were not significant. Infants born preterm usually have low birthweight, and optimal nutrition plays a crucial role in supporting their growth to reduce morbidity in later life [8]. Furthermore, children with birth weight ≤1 kg usually have poorer growth than children with birth weight >1 kg [35,36]. We, therefore, hypothesised that children with birth weight ≤1 kg might be more likely to benefit from early nutritional supplements. However, our findings did not suggest this was the case.

We also hypothesised that growth outcomes might differ for children born at different gestational ages, with a greater effect of supplements in infants born at earlier gestations. We found that supplemented children born very preterm had lower BMI and BMI z-scores during toddlerhood and childhood and lower weight-for-length scores in toddlers than unsupplemented children, but these effects were not seen in children born extremely or moderate to late preterm, although the numbers of included children and trials are smallest in the moderate to late preterm group. The reasons for differences between gestational age at birth groups are not clear but might include greater neonatal illness in the smallest infants, thereby restricting growth in this group, and that weight gain in preterm infants is disproportionately fat [37].

Children who had received supplements post-discharge, but not those who received supplements in hospital, had greater weight, length in infancy, and height z-scores in toddlers, consistent with previous findings of a systematic review [17]. Children who had received supplements in hospital with additional protein also had lower BMI and measures of adiposity into adolescence. However, the separate effects of the timing and composition of the supplements could not be distinguished in our analysis, as some trials studied in-hospital supplements with protein as the primary supplement, whereas other trials studied post-discharge supplementation with multicomponent supplements. Nevertheless, this again indicates that early supplements do not increase later risk of becoming overweight/obesity, and in the specific situation of in-hospital supplements with protein, may actually reduce the risk.

We found supplements increased height in toddlers only if the primary feed was formula, but not if the primary feed was breastmilk or if supplements were provided as both parenteral and enteral feeds. Supplemented infants whose primary feed was formula also had greater length and BMC in infancy but lower head circumference and head circumference z-scores in toddlers, although none of these interaction terms were significant. We had hypothesised that infants whose primary feed was breastmilk would receive less baseline (unsupplemented) nutritional intake and may therefore show greater effects of supplementation. Consistent with this, estimated protein intake was lower if breastmilk was the primary feed, and supplements provided a much greater increase in protein, carbohydrate, and energy intakes than for infants who received formula as primary feed. Therefore, comparison of nutrition intakes cannot explain the effect of supplements in the formula group. Another possible explanation may be the effect of growth-regulating hormones and growth factors in breastmilk. One study found a positive correlation between insulin-like growth factor (IGF)-I and weight z-scores in healthy infants [38]; infants fed formula milk had higher IGF-I levels than those fed breastmilk [39]. Leptin, adiponectin, and cortisol in breast milk could also play roles in the short-term control of food intake and have long-term effects on energy balance and body weight regulation [40,41].

In trials conducted before or in 2000, supplementation increased growth in infancy, but in trials conducted after 2000, supplementation decreased BMI, BMI z-scores, and weight in adolescence and at >3 years. We had expected that nutrition intakes might differ over time, with later studies potentially reporting higher macronutrient intakes in the unsupplemented groups, and hence smaller effects of additional supplements. However, we found no significant differences between the two time epochs in estimated mean baseline intakes nor in the additional macronutrients provided by supplements, although the mean protein intake was slightly higher and had greater variability in the later epoch. The populations of infants included in trials did also not appear to have changed, with similar inclusion criteria used in trials conducted before and after 2000. Another possible reason for the different effects in different epochs may be that the outcomes may be changing in the overall population over this period. For example, average height [42] and the incidence of overweight/obesity [43] have both increased in many populations over the period during which these trials were conducted, although it is not clear how this might alter the effect of early nutritional supplements on these outcomes.

Previously published Cochrane reviews [10,44,45] have investigated the effects of macronutrient supplements for preterm infants only and reported growth outcomes up to toddler age. One systematic review of published studies [17] explored the long-term effects on growth but was limited by high heterogeneity, possibly because of the wide range of ages studied (3–24 months). Therefore, we reported early growth outcomes separately for infancy (≤12 months) and in toddlers (1–3 years) in this IPD-MA. Further, by using participant-level rather than trial-level data in meta-analyses, we were able to explore the heterogeneity by conducting several different subgroup analyses.

Our IPD-MA has some limitations. We identified 42 eligible trials with published post-discharge growth outcomes. However, due to local ethics restrictions, missing datasets or investigators declining to share data, 25 of these trials could not be included. To address this issue, we conducted sensitivity analyses combining both IPD and AD. Most of these analyses gave results consistent with the analysis of IPD only, except for the growth outcomes in infancy, where supplementation appeared to improve early growth in infancy only in the analyses of combined IPD and AD. Furthermore, a post-hoc calculation showed that the analysis of IPD alone (338 children) had sufficient power to detect a 0.55 difference in BMI in childhood with 80% power and alpha of 0.05, 5% type I error. Therefore, it is unlikely that clinically important effects of supplementation were missed. Nevertheless, not all trials were able to provide data for all pre-specified subgroups, which limited the power to be able to detect overall or subgroup differences for some important outcomes. Additionally, a large number of subgroups, multiple outcomes, and time points were analysed, which increases the chance of type 1 error [46], and the subgroup findings should be interpreted with caution as hypothesis-generating. In addition, the included studies were of different types of macronutrient supplements given at different times for different durations. Although we followed the strategies suggested by the Cochrane handbook to address heterogeneity [20], significant unexplained heterogeneity remained for some outcomes. The duration of supplements and actual nutrient intakes for individual participants were not available for most trials, so the subgroup analyses of the duration, timing, and type of supplement were based only on the trial-level information and are therefore imprecise.

We also noticed significant heterogeneity when combining individual results for some outcomes, and this was not all explained in the subgroup analyses. As we were not able to calculate I-squared values, it is hard to quantify the inconsistency across studies. However, statistical heterogeneity is inevitable in a meta-analysis [47], especially when there are many trials included, as the test for heterogeneity has high power to detect a small amount of inconsistency that may be clinically unimportant [20].

Previous observational studies [25,26,27] have reported that early rapid growth is associated with later obesity. However, this IPD-MA found that early macronutrient supplements slightly improve growth in infancy but do not affect childhood adiposity. This apparent discrepancy may be because this IPD-MA focused on the effects of nutrition rather than growth, and the effects of supplementation on early growth may be too early or too small to cause later adverse effects. Alternatively, previous observations may reflect the relationship between other regulators of growth, including genetic and environmental influences, and later adiposity, rather than the effects of nutrition itself.

We conclude that early macronutrient supplements do not alter BMI in childhood for infants born small. Supplements improve growth in infancy, including bone mineralisation, but the effects are small and do not persist beyond the first year. Hypothesis-generating subgroup analyses suggest that in-hospital supplementation with protein may actually reduce later BMI, and that children born SGA are least likely to benefit from macronutrient supplements. There is little evidence of sex-specific effects. The current widespread practice of macronutrient supplementation for preterm and SGA infants is likely to have small effects on short-term growth but is unlikely to increase adiposity in childhood.

## Figures and Tables

**Table 1:** Included trials and their characteristics.

Author/Year	Country	Participants	Participants: *n*	Intervention	Control	Duration	Outcomes
**Studies with IPD Available**							
Agosti 2003	Italy	Inclusion criteria: preterm BW < 1500 g and previously fed with a preterm formula. Exclusion criteria: malformations, intraventricular haemorrhage, periventricular leukomalacia, chronic lung disease, necrotising enterocolitis grade >1, total parenteral nutrition >2 weeks, sepsis, retinopathy of prematurity grade >1.	Intervention: 89 Control: 67	Preterm formula (protein 2.4 g/100 mL, energy 80 kcal/100 mL)	Standard term formula (protein 1.7 g/100 mL, energy 70 kcal/100 mL)	Started from 40 weeks PMA, stopped at 55 weeks PMA	Weight (raw and z-scores), length (raw and z-scores), HC (raw and z-scores), BMI (raw and z-scores), weight-for-length z-scores in infancy and in toddlers.
Atkinson 1999	Canada	Inclusion criteria: BW < 2500 g; GA < 42 weeks. birthweight <5th percentile and fed only formula at entry into the study.	Intervention: 22 Control: 28	Ross Discharge formula (protein: 1.8 g/100 mL, energy 74 kcal/100 mL)	Similac with Iron formula (68 kcal/100 mL)	Started from discharge, stopped at 1 year CA	Weight (raw and z-scores), length (raw and z-scores), HC (raw and z-scores), BMI (raw and z-scores), weight-for-length z-scores, lean mass, lean mass index, fat mass, fat mass index, percent fat mass, BMC, and BMD in infancy.
Biasini 2012	Italy	Inclusion criteria: preterm BW 580 to 1250 g and GA < 32 weeks.	Intervention: 34 Control: 27	Protein supplemented group (protein 4.8 g/kg/day, energy 141 kcal/day)	Control group (protein 3.5 g/kg/day, energy 135 kcal/day)	Started from the first day of full enteral feeding, stopped at discharge.	Weight (raw and z-scores), length (raw and z-scores), HC (raw and z-scores), BMI (raw and z-scores), weight-for-length z-scores in infancy and in toddlers. Weight (raw and z-scores), length (raw and z-scores), BMI (raw and z-scores) in childhood.
Brunton 1998	Canada	Inclusion criteria: preterm BW < 1500 g and appropriate for gestational age, had BPD, were formula-fed by parental choice, and had not undergone gastrointestinal surgery. Exclusion criterion: major congenital anomalies.	Intervention: 26 Control: 30	Enriched formula (protein 2.3 g/100 mL, energy 90 kcal/100 mL)	Standard isoenergetic formula (protein 1.5 g/100 mL, energy 90 kcal/100 mL)	Started at 37 weeks PMA, stopped at 3 months’ CA.	Weight (raw and z-scores), length (raw and z-scores), HC (raw and z-scores), BMI (raw and z-scores), weight-for-length z-scores, BMC and BMD in infancy.
Cooke 1998	UK	Inclusion criteria: GA ≤ 34 weeks and BW ≤ 1750 g, and growing normally at the time of hospital discharge, i.e., ≥25 g/d. Exclusion criterion: systemic disease or require medication.	Intervention: 56 Control: 57	Preterm formula (protein 2.2 g/100 mL, fat 4.4 g/100 mL, carbohydrate 8.5 g/100 mL, energy 80 kcal/100 mL)	Term formula (protein 1.4 g/100 mL, fat 3.6 g/100 mL, carbohydrate 7.5 g/100 mL, energy 66 kcal/100 mL)	Started from discharge, stopped at 6 months’ CA.	Weight (raw and z-scores), length (raw and z-scores), HC (raw and z-scores), BMI (raw and z-scores), weight-for-length z-scores in infancy and in toddlers. Weight (raw), length (raw and z-scores), BMI (raw and z-scores) in adolescence. Lean mass, lean mass index, fat mass, fat mass index, percent fat mass, BMC and BMD in infancy and in adolescence.
Embleton 2005	UK	Inclusion criteria: preterm GA ≤ 34 weeks and BW ≤ 1750 g, tolerating enteral intake ≥150 mL/kg/day for ≥48 h and current weight ≥1000 g.	Formula A: 25 Formula C: 26	Formula A (protein 2.6 g/100 mL, fat 4.3 g/100 mL, carbohydrate 7.9 g/100 mL, energy 80 kcal/100 mL);	Formula C (protein 2.2 g/100 mL, fat 4.5 g/100 mL, carbohydrate 7.9 g/100 mL, energy 80 kcal/100 mL)	Started when full enteral feeding 150 mL/kg/day, stopped at 12 weeks.	Weight (raw and z-scores), length (raw and z-scores), HC (raw and z-scores), BMI (raw and z-scores), weight-for-length z-scores in infancy and in toddlers. Weight (raw), length (raw and z-scores), BMI (raw and z-scores) in adolescence. Lean mass, lean mass index, fat mass, fat mass index, percent fat mass, BMC and BMD in infancy and in adolescence.
Fewtrell 2001	UK	Inclusion criteria: term GA ≥ 37 weeks and BW < 10th centile for gestation and sex (UK growth charts).	Intervention: 152 Control: 147	Enriched formula (protein 1.9 g/100 mL, fat 4.0 g/100 mL, carbohydrate 7.2 g/100 mL, energy 72 kcal/100 mL)	Term formula (protein 1.5 g/100 mL, fat 3.9 g/100 mL, carbohydrate 7.0 g/100 mL, energy 68 kcal/100 mL)	Started within the first week, stopped at 9 months’ CA.	Weight (raw and z-scores), length (raw and z-scores), HC (raw and z-scores), BMI (raw and z-scores), weight-for-length z-scores in infancy and in toddlers.
Koo 2006	USA	Inclusion criteria: preterm GA ≤ 34 weeks and BW 630 to 1620 g, intact gastrointestinal tract, tolerated full enteral feeding, expected soon to be ready for hospital discharge.	Intervention: 44 Control: 45	Nutrient-enriched formula (protein 1.9 g/100 mL, fat 4.1 g/100 mL, carbohydrate 7.6 g/100 mL, energy 74 kcal/100 mL)	Term formula (protein 1.4 g/100 mL, fat 3.6 g/100 mL, carbohydrate 7.2 g/100 mL, and energy 67 kcal/100 mL)	Started at discharge, stopped at 12 months after discharge.	Weight (raw and z-scores), length (raw and z-scores), HC (raw and z-scores), BMI (raw and z-scores), weight-for-length z-scores, lean mass, lean mass index, fat mass, fat mass index, percent fat mass, BMC and BMD in infancy.
Litmanovitz 2007	Israel	Inclusion criteria: preterm BW < 1500 g and appropriate for gestational age, formula-fed. Exclusion criteria: severe central nervous system disorder, major congenital anomalies, chronic lung disease, prior diagnosis of necrotising enterocolitis.	Intervention: 10 Control: 10	Enriched post-discharge formula (protein 1.9 g/100 mL, energy 74 kcal/100 mL)	Term formula (protein 1.5 g/100 mL, energy 67 kcal/100 mL)	Started at term (discharge), stopped at 6 months’ CA.	Weight (raw and z-scores), length (raw and z-scores), HC (raw and z-scores), BMI (raw and z-scores), weight-for-length z-scores in infancy.
Lucas 1996	UK	Inclusion criteria: preterm BW < 1850 g and GA < 37 weeks, survived to be assigned to a study group between 48 and 72 h of age. Exclusion criterion: major congenital anomalies.	Intervention: 137 Control: 138	Fortified human breast milk (fortifier contained protein 0.7 g/100 mL, fat 0.05 g/100 mL, carbohydrate 2.73 g/100 mL, energy 14 kcal/100 mL)	Human breast milk	Started within 48 h, stopped at discharge or reached 2000 g.	Weight (raw and z-scores), length (raw and z-scores), HC (raw and z-scores), BMI (raw and z-scores), weight-for-length z-scores in infancy and in toddlers.
Lucas 2001	UK	Inclusion criteria: preterm GA< 37 weeks and BW < 1750 g. Exclusion criteria: congenital malformations or conditions known to affect growth or development.	Intervention: 113 Control: 116	Post-discharge formula (protein 1.9 g/100 mL, fat 4.0 g/100 mL, carbohydrate 7.2 g/100 mL, energy 72 kcal/100 mL)	Term formula (protein 1.5 g/100 mL, fat 3.8 g/100 mL, carbohydrate 7.0 g/100 mL, energy 68 kcal/100 mL)	Started one week before discharge, stopped at 9 months CA.	Weight (raw and z-scores), length (raw and z-scores), HC (raw and z-scores), BMI (raw and z-scores), weight-for-length z-scores in infancy and in toddlers.
Moltu 2013	Norway	Inclusion criteria: preterm GA < 37 weeks and BW < 1500 g. Exclusion criteria: congenital malformations, chromosomal abnormalities, critical illness with short life expectancy, clinical syndromes known to affect growth and development.	Intervention: 24 Control: 24	Enhanced nutrient: Parenteral nutrition: started with 3.5 g/kg/day AA. Full enteral feeding: intervention group received 10% higher energy and 20% higher protein than the control group.	Standard nutrient: Parenteral nutrition: started with 2.0 g/kg/day AA.	Started within 24 h after birth, stopped at 52 weeks PMA or when reached 5.5 kg.	Weight (raw and z-scores), length (raw and z-scores), HC (raw and z-scores), BMI (raw and z-scores), weight-for-length z-scores in infancy and in toddlers.
Morgan 2014	UK	Inclusion criteria: GA 24–28 weeks and BW < 1200 g. Exclusion criteria: unlikely to survive the first week after birth; diagnosed with major congenital or chromosomal abnormalities known to affect gastrointestinal function or head growth, including definite parenchymal lesions on cranial ultrasound scan in first 48 h.	Intervention: 74 Control: 76	Higher macronutrient content (parenteral intake with protein 3.8 g/kg/day, fat 3.8 g/kg/day, carbohydrate 15.6 g/kg/day, energy 103 kcal/kg/day)	Standard macronutrient content (parenteral intake with protein 2.8 g/kg/day, fat 2.8 g/kg/day, carbohydrate 13.5 g/kg/day, energy 85 kcal/kg/day)	Started within 120 h of birth, stopped at 28 days.	HC (raw and z-scores) in toddlers.
Mukhopadhyay 2007	India	Inclusion criteria: preterm GA ≤ 34 weeks and BW ≤ 1500 g reached feed volume of 150 mL/kg/day, feed constituted at least 80% breast milk. Exclusion criteria: major congenital malformation, gastrointestinal abnormalities.	Intervention: 85 Control: 81	Fortified human milk: (fortifier contained protein 0.4 g/100 mL; fat 0.2 g/100 mL; carbohydrate 2.4 g/100 mL; energy 13 kcal/100 mL)	Exclusive human milk	Started when feed volume reached 150 mL/kg/day, stopped when it reached 2 kg or full breastfeeds.	Weight (raw and z-scores), length (raw and z-scores), HC (raw and z-scores), BMI (raw and z-scores), weight-for-length z-scores in infancy.
Picaud 2008	France	Inclusion criteria: GA ≤ 33 weeks, BW < 1750 g. Exclusion criterion: major congenital malformations.	Intervention: 23 Control: 26	Preterm formula (protein 2.3 g/100 mL, fat 4.2 g/100 mL, carbohydrate 8.5 g/100 mL, energy 81 kcal/100 mL)	Term formula (protein 1.7 g/100 mL, fat 3.2 g/100 mL, carbohydrate 7.85 g/100 mL, energy 67 kcal/100 mL)	Started after discharge, stopped 2 months after discharge.	Weight (raw and z-scores), length (raw and z-scores), HC (raw and z-scores), BMI (raw and z-scores), weight-for-length z-scores, lean mass, lean mass index, fat mass, fat mass index, percent fat mass, BMC and BMD in infancy.
Rochow 2019	Canada	Inclusion criteria: GA < 30 weeks, length of stay > 21 days, and receiving fortified BM. Exclusion criteria: gastrointestinal malformation, major congenital anomalies, necrotising enterocolitis, abdominal surgery, and gram-negative sepsis.	Intervention: 52 Control: 51	Target fortified human milk:(protein 3.0 g/100 mL, fat 4.4 g/100 mL, carbohydrates 8.5 g/100 mL)	Standard fortified human milk	Started when enteral intake was ≥100 mL/kg/day, stopped at 36 weeks’ PMA.	Weight (raw and z-scores), length (raw and z-scores), HC (raw and z-scores), BMI (raw and z-scores), weight-for-length z-scores in infancy and in toddlers. Lean mass, lean mass index, fat mass, fat mass index, and percent fat mass in infancy.
Roggero 2012	Italy	Inclusion criteria: GA ≤ 32 weeks, BW ≤ 1500 g, being fed human milk for <20% of total milk intake. Exclusion criteria: congenital malformations, conditions that interfere with growth or body composition.	Intervention: 110 Control: 107	Nutrient-enriched formula (protein 2.0 g/100 mL, fat 4.1 g/100 mL, carbohydrate 7.5 g/100 mL, energy 75 kcal/100 mL)	Term formula (protein 1.4 g/100 mL, fat 3.7 g/100 mL, carbohydrate 7.4 g/100 mL, energy 68 kcal/100 mL)	Started from term, stopped at 6 months.	Weight (raw and z-scores), length (raw and z-scores), HC (raw and z-scores), BMI (raw and z-scores), weight-for-length z-scores, lean mass, lean mass index, fat mass, fat mass index, percent fat mass in infancy.
Tan 2008	UK	Inclusion criteria: GA < 29 weeks. Exclusion criteria: Triplets and higher multiplicity, admitted after 7 days of age, major congenital abnormalities.	Intervention: 68 Control: 74	Parenteral intake with protein 4 g/kg/day, fat 4 g/kg/day, carbohydrate 16.3 g/kg/day, energy 117 kcal/kg/day; enteral intake breast milk or formula with target protein 4 g/kg/day, energy 133–150 kcal/kg/day	Parenteral intake with protein 3 g/kg/day, fat 3 g/kg/day, carbohydrate 13.5 g/kg/day, energy 93 kcal/kg/day; enteral intake breast milk or formula with target protein 3.3 g/kg/day, energy 133 kcal/kg/day	Started when infants received parenteral and enteral nutrition from the first week, stopped at 34 weeks’ PMA.	Weight (raw and z-scores), length (raw and z-scores), HC (raw and z-scores), BMI (raw and z-scores), weight-for-length z-scores in infancy.
Zachariassen 2001	Denmark	Inclusion criteria: preterm GA ≤ 32 weeks, breastfeeding. Exclusion criteria: severe diseases, circumstances influencing eating and feeding ability at discharge.	Intervention: 105 Control: 102	Fortified mother’s milk. Component of fortifier: (protein 1.4 g/day, energy 17.5 kcal/day)	Unfortified mother’s milk	Started shortly before discharge, stopped at 4 months’ CA.	Weight (raw and z-scores), length (raw and z-scores), HC (raw and z-scores), BMI (raw and z-scores), weight-for-length z-scores in infancy, in toddlers and in childhood. Lean mass, lean mass index, fat mass, fat mass index, and percent fat mass in childhood.
**Studies with AD Available**							
Amesz 2010	The Netherlands	Inclusion criteria: preterm GA ≤ 32 weeks or BW ≤ 1500 g. Exclusion criteria: congenital malformations or conditions known to affect growth or body composition (e.g., severe BPD, inborn error of metabolism, cardiac or renal disease, necrotising enterocolitis with substantial gut loss, grade 4 intraventricular haemorrhage).	Intervention: 52 Control:50	Post-discharge formula (protein 1.7 g/100 mL, fat 3.5 g/100 mL, carbohydrate 7.0 g/100 mL, energy 67 kcal/100 mL)	Term formula (protein 1.47 g/100 mL, fat 3.5 g/100 mL, carbohydrate 7.2 g/100 mL, energy 70 kcal/100 mL)	Started from term, stopped at 6 months’ CA.	Weight, length, BMI, fat mass, lean mass and BMC, BMD in infancy and in childhood
Bellagamba 2016	Italy	Inclusion criteria: preterm BW 500 to 1249 g.	Intervention: 82 Control: 82	High protein intake group (protein supplementation started at 1.5 g/kg/day and increased by 0.5 g/kg/day to a maximum of 3.5 g/kg/day on the fifth day after birth)	Standard protein intake group (protein supplementation started at 1.5 g/kg/day and increased by 0.5 g/kg/day to a maximum of 2.5 g/kg/day on the third day after birth)	Started from birth, stopped at discharge.	Weight, length and HC in toddlers.
Brooke 1985	UK	Inclusion criteria: term SGA infants with their parents’ consent. Exclusion criterion: infants underweight because of wasting.	Intervention: 10 Control: 7	High energy formula (protein 2.3 g/100 mL, fat 4.2 g/100 mL, carbohydrate 10.7 g/100 mL, energy 87 kcal/100 mL)	Standard energy formula (protein 1.5 g/100 mL, fat 3.6 g/100 mL, carbohydrate 7.2 g/100 mL, energy 65 kcal/100 mL)	Started from the second week after birth, stopped at 3 months postnatal age.	Weight, length, and HC in infancy.
Carver 2001	USA	Inclusion criteria: preterm BW < 1800 g or GA < 37 weeks, previous parental decision not to provide breast milk. Exclusion criteria: severe BPD, cardiac, respiratory, gastrointestinal, or other systemic diseases.	Intervention: 67 Control: 56	Post-discharge formula (protein 1.9 g/100 mL, fat 4.0 g/100 mL, carbohydrate 7.6 g/100 mL, energy 74 kcal/100 mL)	Term formula (protein 1.4 g/100 mL, fat 3.6 g/100 mL, carbohydrate 7.2 g/100 mL energy 67 kcal/100 mL)	Started 2 to 4 days before discharge, stopped at 12 months’ CA.	Weight, length, and HC in infancy.
Chan 1994	USA	Inclusion criteria: preterm BW < 1650 g and weight at hospital discharge ≥1800 gExclusion criteria: necrotising enterocolitis, congenital anomalies, hepatic disease, cardiac disease, BPD.	Preterm formula: 14 Low birthweight formula: 14 Standard formula: 15	Preterm formula (protein 1.85–1.94 g/100 mL, fat 3.75–3.83 g/100 mL, carbohydrate 7.1–7.2 g/100 mL, energy 69.4–71.0 kcal/100 mL); Low birthweight formula (protein 1.71–1.78 g/100 mL, fat 3.62–3.81 g/100 mL, carbohydrate 6.8–7.1 g/100 mL, energy 66.7–66.9 kcal/100 mL)	Standard formula (protein 1.52–1.54 g/100 mL, fat 3.64–3.74 g/100 mL, carbohydrate 7.1–7.3 g/100 mL, energy 67.4–689 kcal/100 mL)	Started from discharge, stopped 8 weeks after discharge.	Weight and BMC at in infancy.
Cooper 1985	South Africa	Inclusion criteria: preterm BW 1200–1500 g and GA < 36 weeks. Exclusion criteria: needing ventilator support.	Intervention: 4 Control: 4	Preterm formula (protein 1.94 g/100 mL, fat 3.4 g/100 mL, carbohydrate 7.3 g/100 mL, energy 67 kcal/100 mL)	Standard formula (protein 1.5 g/100 mL, fat 1.6 g/100 mL, carbohydrate 7.2 g/100 mL, energy 67 kcal/100 mL)	Started when reached a weight of 2000 g, stopped after 8 weeks.	Weight, length, and HC in infancy.
De Curtis 2002	Italy	Inclusion criteria: preterm BW < 1750 g and GA< 35 weeks. Exclusion criterion: infants with clinical problems.	Intervention: 16 Control: 17	Post-discharge formula (protein 1.8 g/100 mL, fat 4.1 g/100 mL, carbohydrate 7.5 g/100 mL, energy 74 kcal/100 mL)	Term formula (protein 1.4 g/100 mL, fat 3.6 g/100 mL, carbohydrate 7.1 g/100 mL, energy 66 kcal/100 mL)	Started from start of enteral feeds, stopped after 2 months.	Weight, length, HC, fat mass, lean mass, and BMC in infancy.
Dogra 2017	India	Inclusion criteria: preterm BW < 1500 g or GA < 32 weeks and reached a feed volume of 100 mL/kg/day. Exclusion criterion: lethal congenital malformations.	Intervention: 59 Control: 56	Fortified breast milk with higher protein intake (fortifier contained protein 1.0 g/100 mL, fat 0.01 g/100 mL, carbohydrate 3.6 g/100 mL, energy 17.2 kcal/100 mL)	Fortified breast milk with standard protein intake (fortifier contained protein 0.4 g/100 mL, fat 0.2 g/100 mL, carbohydrate 2.4 g/100 mL; energy 13 kcal/100 mL)	Started when reached a feed volume of 100 mL/kg/day, stopped at discharge or when totally directly breast-fed, whichever was earlier.	Weight, length, and HC in toddlers.
Jeon 2011	Korea	Inclusion criteria: preterm GA < 33 weeks and BW < 1500 g, formula as the primary food source. Exclusion criteria: chromosomal disorders, serious congenital malformations at discharge that would affect growth and development.	Intervention: 35 Control: 34	Preterm formula (protein 2.3 g/100 mL, fat 4.1 g/100 mL, carbohydrate 8.5 g/100 mL, energy 80 kcal/100 mL)	Term formula (protein 1.6 g/100 mL, fat 3.5 g/100 mL, carbohydrate 7.2 g/100 mL, energy 67 kcal/100 mL)	Started at term, stopped at 6 months’ CA.	Weight (raw and z-scores), length, (raw and z-scores), and HC (raw and z-scores) in infancy and in toddlers.
Lin 2004	China	Inclusion criteria: term GA ≥ 37 weeks and birthweight < 10 th centile. Exclusion criteria: severe medical problems or breast-fed.	Intervention: 20 Control: 20	Nutrient-enriched formula (protein 1.9 g/100 mL, fat 4.1 g/100 mL, carbohydrate 7.7 g/100 mL, energy 74 kcal/100 mL)	Term formula (protein 1.4 g/100 mL, fat 3.7 g/100 mL, carbohydrate 7.1 g/100 mL, energy 68 kcal/100 mL)	Started at discharge, stopped at 3 months after discharge.	Weight, length, and HC in infancy.
Lucas 1989	UK	Inclusion criteria: preterm GA < 37 weeks and BW < 1850 g. Exclusion criteria: major congenital abnormality known to impair growth or development, died before randomisation within the first 48 h	(1) Lucas 1989a Intervention: 76 Control: 83	(1) Lucas 1989a: Preterm formula as sole diet (protein 2.0 g/100 mL, fat 4.9 g/100 mL, carbohydrate 7.0 g/100 mL, energy 80 kcal/100 mL)	(1) Lucas 1989a: Banked breast milk as sole diet (protein 1.1 g/100 mL, fat 1.7 g/100 mL, carbohydrate 7.1 g/100 mL, energy 46 kcal/100 mL)	Started within 48 h, stopped at discharge or reached 2000 g.	Weight, length and HC in infancy, in childhood, and in adolescence. BMD and BMC in adolescence.
			(2) Lucas 1989b: Intervention: 173 Control: 170	(2) Lucas 1989b Preterm formula as a supplement	(2) Lucas 1989 b: banked breast milk as a supplement;		
			(3) Lucas 1989c: combined Lucas 1989a and Lucas 1989b	(3) Lucas 1989c: combined Lucas 1989a and Lucas 1989b	(3) Lucas 1989c: combined Lucas 1989a and Lucas 1989b		
Lucas 1990	UK	Inclusion criteria: preterm GA < 37 weeks and BW < 1850g. Exclusion criteria: major congenital abnormality known to impair growth or development, died before randomisation within the first 48 h.	(1) Lucas 1990a:Intervention: 81 Control: 79	(1) Lucas 1990a: Preterm formula as sole diet (protein 2.0 g/100 mL, fat 4.9 g/100 mL, carbohydrate 7.0 g/100 mL, energy 80 kcal/100 mL)	(1) Lucas 1990a: Term formula as sole diet (protein 1.5 g/100 mL, fat 3.8 g/100 mL, carbohydrate 7.0 g/100 mL, energy 68 kcal/100 mL)	Started within 48 h, stopped at discharge or reached 2000 g	Weight, length and HC in infancy, in childhood, and in adolescence. BMD and BMC in adolescence.
			(2) Lucas 1990b: Intervention: 132 Control: 132	(2) Lucas 1990b Preterm formula as supplement	(2) Lucas 1990b: Term formula as supplement		
			(3) Lucas 1990c: Combined Lucas 1990a and Lucas 1990b Intervention: 213 Control: 211	(3) Lucas 1990c: Combined Lucas 1990a and Lucas 1990b	(3) Lucas 1990c: Combined Lucas 1990a and Lucas 1990b		
Lucas 1992	UK	Inclusion criteria: preterm BW < 1850 g and GA < 37 weeks, formula-fed during hospital stay. Exclusion criteria: congenital malformations and disease likely to influence growth and neurodevelopment.	Intervention: 16 Control: 15	Post-discharge formula (protein 1.9 g/100 mL, fat 4.0 g/100 mL, carbohydrate 7.3 g/100 mL, energy 72 kcal/100 mL)	Term formula (protein 1.5 g/100 mL, energy 67 kcal/100 mL, fat and carbohydrate not specified)	Started before discharge, stopped at 9 months’ CA.	Weight, length, HC and BMC in infancy.
O’Connor 2008	Canada	Inclusion criteria: preterm GA < 33 weeks, BW 750 to 1800 g, received ≥80% of their total feedings as human milk 3 days before hospital discharge. Exclusion criteria: serious congenital or chromosomal anomalies that could affect growth, grade 3 or 4 periventricular or intraventricular haemorrhage, oral steroids within 14 days of randomisation, severe asphyxia, known maternal alcohol or drug abuse.	Intervention: 19 Control: 20	Human milk with multi-nutrient fortifier (protein 2.0 g/100 mL, fat 4.2 g/100 mL, carbohydrate 8.8 g/100 mL, energy 81 kcal/100 mL)	Unfortified human milk (protein 1.3 g/100 mL, fat 3.9 g/100 mL, carbohydrate 7.2 g/100 mL, energy 68 kcal/100 mL)	Started from discharge, stopped at 12 weeks after discharge.	Weight, length, HC, fat mass, lean mas BMC and BMD in infancy.
Svenningsen 1982	Sweden	Inclusion criteria: very low birthweight preterm infants with mean ± SD: BW 1385 ± 343 g and GA 30.8 ± 2.9 weeks.	Intervention: 16 Control: 14	Nutrition enriched formula (protein 2.1 g/100 mL, energy 69.5 kcal/100 mL)	Standard formula (protein 1.6 g/100 mL, energy 68.5 kcal/100 mL)	Started from the third week after birth, stopped at the seventh week after birth.	Weight, length, and HC in infancy and in toddlers.
Wauben 1998	Canada	Inclusion criteria: BW < 1800 g, appropriate weight-for-gestational-age, postnatal age >1 week, full oral foods (no parenteral nutrition and >160 mL/kg/day enterally) tolerated for ≥5 days, weight gain >10 g/kg/day. Exclusion criteria: severe congenital malformation, chromosomal abnormalities, gastrointestinal disease.	Intervention: 12 Control: 13	Mother’s milk with multi-nutrient fortifier (fortifier protein 0.4 g/100 mL, fat 34.7 g/100 mL)	Mother’s milk supplemented with calcium and phosphorus	Started when expressed mother’s milk >80% of total enteral intake, stopped when discharged if PMA > 38 weeks.	Weight, length, HC, percent fat mass, BMC, BMD in infancy.
Wheeler 1996	USA	Inclusion criteria: preterm GA < 35 weeks and BW < 1800 g; normally grown in utero, free of medical conditions that affect nutrition and growth. Exclusion criteria: necrotising enterocolitis, chronic lung disease (oxygen need after hospital discharge), central nervous system disease, other conditions affecting nutrient intake or anthropometrics.	Intervention: 23 Control: 20	Whey predominant premature infant formula (protein 1.8 g/100 mL, fat 3.7 g/100 mL, carbohydrate 7.2 g/100 mL, energy 68 kcal/100 mL)	Standard formula (protein 1.5 g/100 mL, fat 3.6 g/100 mL, carbohydrate 7.2 g/100 mL, energy 68 kcal/100 mL)	Started 1 week before hospital discharge, stopped 8 weeks after discharge.	Weight, length, and HC in infancy.

Abbreviations: IPD: individual participant data; AD: aggregated data; GA: gestational age; PMA: postmenstrual age; BW: birthweight; CA: corrected age; SGA: small-for-gestational-age; SD: standard deviation; HC: head circumference; BMI: body mass index; BMC: bone mineral content; BMD: bone mineral density; BPD: bronchopulmonary dysplasia; The references of the studies are presented in Table S3.

## Data Availability

The de-identified participant data analysed for the ESSENCE IPD-MA project remain the property of the ESSENCE-IPD Trialist Group. Researchers should contact the original trial investigator directly for access to these data. The data dictionary, statistical analysis plan and metadata for this IPD-MA are available at doi:10.17608/k6.auckland.18729068.v1, accessed on 30 January 2021. Researchers are able to use this information and the provided contact address (researchhub@auckland.ac.nz) to request further information through the Data Access Committee of the Liggins Institute.

## Supplementary Materials

The following supporting information can be downloaded at: https://www.mdpi.com/article/10.3390/nu14020392/s1, Text S1. PRISMA-IPD Checklist of items to include when reporting a systematic review and meta-analysis of individual participant data (IPD); Text S2. Comparison of macronutrient intakes; Text S3. References of Table 1; Figure S1. Combined IPD and AD analysis of BMI; table Table S2. IPD analysis of BMI z-scores; Figure S3. IPD analysis of weight; Figure S4. Combined IPD and AD analysis of weight; Figure S5. Forest plot of the effect of macronutrient supplementation on weight z-scores; Figure S6. IPD analysis of length/height; Figure S7. Combined IPD and AD analysis of length/height; Figure S8. Forest plot of the effect of macronutrient supplementation on length/height z-scores; Figure S9. IPD analysis of weight for length z-scores; Figure S10. IPD analysis of head circumference; Figure S11. Combined IPD and AD analysis of head circumference; Figure S12. Forest plot of the effect of macronutrient supplementation on head circumference z-scores; Figure S13. Forest plot of the effect of macronutrient supplementation on fat mass; Figure S14. Forest plot of the effect of macronutrient supplementation on fat mass index; Figure S15. Forest plot of the effect of macronutrient supplementation on percent fat mass; Figure S16. Forest plot of the effect of macronutrient supplementation on lean mass; Figure S17. Forest plot of the effect of macronutrient supplementation on lean mass index; Figure S18. Forest plot of the effect of macronutrient supplementation on bone mineral content; Figure S19. Forest plot of the effect of macronutrient supplementation on bone mineral density; Table S1. Risk of bias within studies; Table S2. Subgroup analyses of infant sex; Table S3. Subgroup analyses of size for the gestation of the infant; Table S4. Subgroup analyses of the size of an infant at birth; Table S5. Subgroup analyses of the gestational age of the infant at birth; Table S6. Subgroup analyses of the timing of supplements; Table S7. Subgroup analyses of type of supplement; Table S8. Subgroup analyses of primary milk feed; Table S9. Subgroup analyses of different epochs; Table S10. Search strategies.
