# Supplementary material for: Sex-Specific Effects of Nutritional Supplements for Infants Born Early or Small: An Individual Participant Data Meta-Analysis (ESSENCE IPD-MA) II: Growth
Source: Nutrients. 2022 Jan 17;14(2):392. doi: 10.3390/nu14020392 (PMC8781781; doi:10.3390/nu14020392)
Supplement: Supplementary file 1 [file nutrients-14-00392-s001.zip › nutrients-1500556-supplementary-done.pdf]

nutrients

Supplementary

Text S1. PRISMA-IPD Checklist of items to include when reporting a systematic review and meta-analysis of individual participant data (IPD)
Text S2. Comparison of macronutrient intakes
Text S3. References of Table 1
Figure S1. Combined IPD and AD analysis of BMI
Figure S2. IPD analysis of BMI z-scores
Figure S3. IPD analysis of weight
Figure S4. Combined IPD and AD analysis of weight
Figure S5. Forest plot of effect of macronutrient supplementation on weight z-scores
Figure S6. IPD analysis of length/height
Figure S7. Combined IPD and AD analysis of length/height
Figure S8. Forest plot of effect of macronutrient supplementation on length/height z-scores
Figure S9. IPD analysis of weigh for length z-scores
Figure S10. IPD analysis of head circumference.
Figure S11. Combined IPD and AD analysis of head circumference
Figure S12. Forest plot of effect of macronutrient supplementation on head circumference z-scores
Figure S13. Forest plot of effect of macronutrient supplementation on fat mass
Figure S14. Forest plot of effect of macronutrient supplementation on fat mass index.
Figure S15. Forest plot of effect of macronutrient supplementation on percent fat mass
Figure S16. Forest plot of effect of macronutrient supplementation on lean mass
Figure S17. Forest plot of effect of macronutrient supplementation on lean mass index
Figure S18. Forest plot of effect of macronutrient supplementation on bone mineral content
Figure S19. Forest plot of effect of macronutrient supplementation on bone mineral density
Table S1. Risk of bias within studies
Table S2. Subgroup analyses of infant sex
Table S3. Subgroup analyses of size for gestation of the infant
Table S4. Subgroup analyses of size of infant at birth
Table S5. Subgroup analyses of gestational age of infant at birth
Table S6. Subgroup analyses of timing of supplements
Table S7. Subgroup analyses of type of supplement
Table S8. Subgroup analyses of primary milk feed
Table S9. Subgroup analyse of different epochs
Table S10. Search strategies
Table S11. List of excluded studies

Text S1. PRISMA-IPD Checklist of items to include when reporting a systematic review and meta-analysis of individual participant data (IPD)

PRISMA-IPD Section/topic	Item No	Checklist item	Reported on page
**Title**			
Title	1	Identify the report as a systematic review and meta-analysis of individual participant data.	Title page
**Abstract**			
Structured summary	2	Provide a structured summary including as applicable:	Abstract, Title page
		**Background**: state research question and main objectives, with information on participants, interventions, comparators and outcomes.	
		**Methods**: report eligibility criteria; data sources including dates of last bibliographic search or elicitation, noting that IPD were sought; methods of assessing risk of bias.	
		**Results**: provide number and type of studies and participants identified and number (%) obtained; summary effect estimates for main outcomes (benefits and harms) with confidence intervals and measures of statistical heterogeneity. Describe the direction and size of summary effects in terms meaningful to those who would put findings into practice.	
		**Discussion:** state main strengths and limitations of the evidence, general interpretation of the results and any important implications.	
		**Other:** report primary funding source, registration number and registry name for the systematic review and IPD meta-analysis.	
Introduction			
Rationale	3	Describe the rationale for the review in the context of what is already known.	Introduction: paragraph 1-2
Objectives	4	Provide an explicit statement of the questions being addressed with reference, as applicable, to participants, interventions, comparisons, outcomes and study design (PICOS). Include any hypotheses that relate to particular types of participant-level subgroups.	Introduction: paragraph 3
Methods			
Protocol and registration	5	Indicate if a protocol exists and where it can be accessed. If available, provide registration information including registration number and registry name. Provide publication details, if applicable.	Methods: paragraph 1
Eligibility criteria	6	Specify inclusion and exclusion criteria including those relating to participants, interventions, comparisons, outcomes, study design and characteristics (e.g. years when conducted, required minimum follow-up). Note whether these were applied at the study or individual level i.e. whether eligible participants were included (and ineligible participants excluded) from a study that included a wider population than specified by the review inclusion criteria. The rationale for criteria should be stated.	Methods: 2.2
Identifying studies - information sources	7	Describe all methods of identifying published and unpublished studies including, as applicable: which bibliographic databases were searched with dates of coverage; details of any hand searching including of conference proceedings; use of study registers and agency or company databases; contact with the original research team and experts in the field; open adverts and surveys. Give the date of last search or elicitation.	Methods: 2.1

Identifying studies - search	8	Present the full electronic search strategy for at least one database, including any limits used, such that it could be repeated.	Table S10
Study selection processes	9	State the process for determining which studies were eligible for inclusion.	Methods: 2.2
Data collection processes	10	Describe how IPD were requested, collected and managed, including any processes for querying and confirming data with investigators. If IPD were not sought from any eligible study, the reason for this should be stated (for each such study).	Methods: 2.2, 2.4, 2.5
		If applicable, describe how any studies for which IPD were not available were dealt with. This should include whether, how and what aggregate data were sought or extracted from study reports and publications (such as extracting data independently in duplicate) and any processes for obtaining and confirming these data with investigators.	
Data items	11	Describe how the information and variables to be collected were chosen. List and define all study level and participant level data that were sought, including baseline and follow-up information. If applicable, describe methods of standardising or translating variables within the IPD datasets to ensure common scales or measurements across studies.	Methods: 2.2, 2.4, 2.5
IPD integrity	A1	Describe what aspects of IPD were subject to data checking (such as sequence generation, data consistency and completeness, baseline imbalance) and how this was done.	Methods: 2.3
Risk of bias assessment in individual studies.	12	Describe methods used to assess risk of bias in the individual studies and whether this was applied separately for each outcome. If applicable, describe how findings of IPD checking were used to inform the assessment. Report if and how risk of bias assessment was used in any data synthesis.	Methods: 2.3
Specification of outcomes and effect measures	13	State all treatment comparisons of interests. State all outcomes addressed and define them in detail. State whether they were pre-specified for the review and, if applicable, whether they were primary/main or secondary/additional outcomes. Give the principal measures of effect (such as risk ratio, hazard ratio, difference in means) used for each outcome.	Methods: 2.2, 2.4, 2.5
Synthesis methods	14	Describe the meta-analysis methods used to synthesise IPD. Specify any statistical methods and models used. Issues should include (but are not restricted to):<br>• Use of a one-stage or two-stage approach.<br>• How effect estimates were generated separately within each study and combined across studies (where applicable).<br>• Specification of one-stage models (where applicable) including how clustering of patients within studies was accounted for.<br>• Use of fixed or random effects models and any other model assumptions, such as proportional hazards.<br>• How (summary) survival curves were generated (where applicable).<br>• Methods for quantifying statistical heterogeneity (such as I^2 and τ^2).<br>• How studies providing IPD and not providing IPD were analysed together (where applicable).<br>• How missing data within the IPD were dealt with (where applicable).	Methods: 2.4, 2.5

Exploration of variation in effects	A2	If applicable, describe any methods used to explore variation in effects by study or participant level characteristics (such as estimation of interactions between effect and covariates). State all participant-level characteristics that were analysed as potential effect modifiers, and whether these were pre-specified.	Methods: 2.4, 2.5
Risk of bias across studies	15	Specify any assessment of risk of bias relating to the accumulated body of evidence, including any pertaining to not obtaining IPD for particular studies, outcomes or other variables.	Methods: 2.3
Additional analyses	16	Describe methods of any additional analyses, including sensitivity analyses. State which of these were pre-specified.	Methods: 2.4, 2.5
**Results**			
Study selection and IPD obtained	17	Give numbers of studies screened, assessed for eligibility, and included in the systematic review with reasons for exclusions at each stage. Indicate the number of studies and participants for which IPD were sought and for which IPD were obtained. For those studies where IPD were not available, give the numbers of studies and participants for which aggregate data were available. Report reasons for non-availability of IPD. Include a flow diagram.	Results: 3.1, Figure 1
Study characteristics	18	For each study, present information on key study and participant characteristics (such as description of interventions, numbers of participants, demographic data, unavailability of outcomes, funding source, and if applicable duration of follow-up). Provide (main) citations for each study. Where applicable, also report similar study characteristics for any studies not providing IPD.	Table 1
IPD integrity	A3	Report any important issues identified in checking IPD or state that there were none.	Results: search result
Risk of bias within studies	19	Present data on risk of bias assessments. If applicable, describe whether data checking led to the up-weighting or down-weighting of these assessments. Consider how any potential bias impacts on the robustness of meta-analysis conclusions.	Results: 3.2 Table S1
Results of individual studies	20	For each comparison and for each main outcome (benefit or harm), for each individual study report the number of eligible participants for which data were obtained and show simple summary data for each intervention group (including, where applicable, the number of events), effect estimates and confidence intervals. These may be tabulated or included on a forest plot.	Results: 3.3-3.6, Figure 2, Figure 3, Figure S1-S19
Results of syntheses	21	Present summary effects for each meta-analysis undertaken, including confidence intervals and measures of statistical heterogeneity. State whether the analysis was pre-specified, and report the numbers of studies and participants and, where applicable, the number of events on which it is based.	Results: 3.3-3.7, Figure 2, Figure 3, Figure S1-S19
		When exploring variation in effects due to patient or study characteristics, present summary interaction estimates for each characteristic examined, including confidence intervals and measures of statistical heterogeneity. State whether the analysis was pre-specified. State whether any interaction is consistent across trials.	
		Provide a description of the direction and size of effect in terms meaningful to those who would put findings into practice.	
Risk of bias across studies	22	Present results of any assessment of risk of bias relating to the accumulated body of evidence, including any pertaining to the availability and representativeness of available studies, outcomes or other variables.	Results: 3.2 Table S1

Additional analyses	23	Give results of any additional analyses (e.g. sensitivity analyses). If applicable, this should also include any analyses that incorporate aggregate data for studies that do not have IPD. If applicable, summarise the main meta-analysis results following the inclusion or exclusion of studies for which IPD were not available.	Results: 3.3-3.7, Figure 2, Figure 3, Figure S1-S19, Table S2-S9
**Discussion**			
Summary of evidence	24	Summarise the main findings, including the strength of evidence for each main outcome.	Discussion: paragraph 1-2, Fig 4, Fig 5
Strengths and limitations	25	Discuss any important strengths and limitations of the evidence including the benefits of access to IPD and any limitations arising from IPD that were not available.	Discussion: paragraph 12-13
Conclusions	26	Provide a general interpretation of the findings in the context of other evidence.	Discussion: paragraph 14
Implications	A4	Consider relevance to key groups (such as policy makers, service providers and service users). Consider implications for future research.	Discussion: paragraph 14
**Funding**			
Funding	27	Describe sources of funding and other support (such as supply of IPD), and the role in the systematic review of those providing such support.	Funding, acknowledge, author contribution

A1 – A3 denote new items that are additional to standard PRISMA items. A4 has been created as a result of re-arranging content of the standard PRISMA statement to suit the way that systematic review IPD meta-analyses are reported.

© Reproduced with permission of the PRISMA IPD Group, which encourages sharing and reuse for non-commercial purpose

Text S2. Comparison of macronutrient intake

a. Macronutrient intakes between trials using formula as primary feed and breast milk as primary feed.

To explore whether the differences in effects of supplements between infants receiving breast milk or formula as their primary feed were due to different baseline macronutrient intakes or quantity of supplements, we compared the mean macronutrient intakes in the unsupplemented groups receiving breast milk or formula as their primary feed, and the mean difference in intakes between supplemented and unsupplemented groups. Infants in the unsupplemented group who received formula as their primary feed had higher protein intake, but similar fat, carbohydrate and energy intakes to those whose primary feed was breastmilk. However, amongst infants who received breastmilk as their primary feed, those in the supplemented group received more protein, energy and carbohydrate than those in unsupplemented group, whereas amongst infants who received formula as their primary feed, the supplemented formula group received much smaller increases in protein, energy and carbohydrate than the unsupplemented group.

	Breast milk		Formula		P Value
	Mean	SD	Mean	SD	
**Mean intakes in the unsupplemented groups**					
Protein (g/100 ml)	1.43	0.24	1.64	0.33	0.26
Fat (g/100 ml)	4	0.49	3.94	0.39	0.84
Carbohydrate (g/100 ml)	6.53	2.33	7.26	0.45	0.49
Energy (g/100 ml)	68	5.29	70.17	4.92	0.48
**Mean differences intakes between supplemented and unsupplemented groups**					
Protein (g/100 ml)	0.92	0.49	0.46	0.15	0.07
Fat (g/100 ml)	0.06	0.73	0.14	0.21	0.84
Carbohydrate (g/100 ml)	2.15	0.46	0.24	0.17	0.0001
Energy (g/100 ml)	11.5	6.89	5.17	3.37	0.07
The composition information for formulae were from IPD or extracted from the publications, and the composition of breastmilk was from IPD or estimated according to the recent guideline[1].					

b. Macronutrient intakes between trials conducted up to 2000 and those conducted after 2000.

To explore whether the differences in effects of supplements between trials conducted before or after 2000 were due to gradual increases in baseline macronutrient intakes over time, we compared the mean macronutrient intakes in the unsupplemented groups in trials conducted before or after 2000, and the mean differences in intakes between supplemented and unsupplemented groups. This showed that there were no significant differences between the two epochs in mean baseline intakes or in mean differences in intake between supplemented and unsupplemented groups for protein, fat, carbohydrate or energy.

	Before and during 2000		After 2000		P Value
	Mean	SD	Mean	SD	
**Mean intakes in the unsupplemented groups**					
Protein (g/100 ml)	1.46	0.15	1.58	0.38	0.52
Fat (g/100 ml)	3.87	0.25	4.05	0.53	0.54
Carbohydrate (g/100 ml)	7.03	0.12	6.68	2.39	0.78
Energy (g/100 ml)	68	1.1	70.17	7.14	0.48
**Mean differences intakes between supplemented and unsupplemented groups**					
Protein (g/100 ml)	0.54	0.15	0.86	0.55	0.23

Fat (g/100 ml)	0.18	0.15	0.03	0.73	0.69
Carbohydrate (g/100 ml)	0.92	1.21	1.6	0.95	0.39
Energy (g/100 ml)	7.5	3.89	9.17	8.13	0.66
The composition information for formulae were from IPD or extracted from the publications, and the composition of breastmilk was from IPD or estimated according to the recent guideline[1].					

References

1. National Health & Medical Research Council (NHMRC). Dietary guidelines for children and adolescents in Australia - incorporating the infant feeding guidelines for health workers. Australia: The National Health and Medical Research Council; 2003 [updated 10 April 2003; cited 2019 17 June]. Available from: http://childaustralia.mrooms.net/pluginfile.php/4134/mod_page/content/38/diet-guidelines.pdf

Text S3. References of Table 1

Agosti 2003

Agosti M, Vegni C, Calciolari G, Marini A, Group GS. Post-discharge nutrition of the very low-birthweight infant: interim results of the multicentric GAMMA study. Acta Paediatr Suppl. 2003;91(441):39-43.

Atkinson 1999

Atkinson SA, Randall-Simpson J, Chang M, Paes B. Randomized trial of feeding nutrient-enriched vs standard formula to premature infants during the first year of life. Pediatr Res. 1999;45:276.

Biasini 2012

Biasini A, Marvulli L, Neri E, China M, Stella M, Monti F. Growth and neurological outcome in ELBW preterms fed with human milk and extra-protein supplementation as routine practice: do we need further evidence? J Matern Fetal Neonatal Med. 2012;25 Suppl 4:72-4.

Brunton 1998

Brunton JA, Saigal S, Atkinson SA. Growth and body composition in infants with bronchopulmonary dysplasia up to 3 months corrected age: a randomized trial of a high-energy nutrient-enriched formula fed after hospital discharge. J Pediat. 1998;133(3):340-5.

Cooke 1998

Cooke RJ, Embleton ND, Griffin IJ, Wells JC, McCormick KP. Feeding preterm infants after hospital discharge: growth and development at 18 months of age. Pediatr Res. 2001;49(5):719-22.

Embleton 2005

Embleton ND, Cooke RJ. Protein requirements in preterm infants: effect of different levels of protein intake on growth and body composition. Pediatr Res. 2005;58(5):855-60.

Fewtrell 2001

Fewtrell MS, Morley R, Abbott RA, Singhal A, Stephenson T, MacFadyen UM, et al. Catch-up growth in small-for-gestational-age term infants: a randomized trial. Am J Clin Nutr. 2001;74(4):516-23.

Koo 2006

Koo WW, Hockman EM. Posthospital discharge feeding for preterm infants: effects of standard compared with enriched milk formula on growth, bone mass, and body composition. Am J Clin Nutr. 2006;84(6):1357-64。

Litmanovitz 2007

Litmanovitz I, Eliakim A, Arnon S, Regev R, Bauer S, Shainkin-Kestenbaum R, et al. Enriched post-discharge formula versus term formula for bone strength in very low birth weight infants: a longitudinal pilot study. J Perinat Med. 2007;35(5):431-5.

Lucas 1996

Lucas A, Fewtrell MS, Morley R, Lucas PJ, Baker BA, Lister G, et al. Randomized outcome trial of human milk fortification and developmental outcome in preterm infants. Am J Clin Nutr. 1996;64(2):142-51.

Lucas 2001

Lucas A, Fewtrell MS, Morley R, Singhal A, Abbott RA, Isaacs E, et al. Randomized trial of nutrient-enriched formula versus standard formula for postdischarge preterm infants. Pediatrics. 2001;108(3):703-11.

Moltu 2013

Moltu SJ, Strommen K, Blakstad EW, Almaas AN, Westerberg AC, Braekke K, et al. Enhanced feeding in very-low-birth-weight infants may cause electrolyte disturbances and septicemia--a randomized, controlled trial. Clin Nutr. 2013;32(2):207-12.

Morgan 2014

Morgan C, McGowan P, Herwitker S, Hart AE, Turner MA. Postnatal head growth in preterm infants: a randomized controlled parenteral nutrition study. Pediatrics. 2014;133(1):e120-8.

Mukhopadhyay 2007

Mukhopadhyay K, Narnag A, Mahajan R. Effect of human milk fortification in appropriate for gestation and small for gestation preterm babies: a randomized controlled trial. Indian Pediatr. 2007;44(4):286-90.

Picaud 2008

Picaud JC, Decullier E, Plan O, Pidoux O, Bin-Dorel S, van Egroo LD, et al. Growth and bone mineralization in preterm infants fed preterm formula or standard term formula after discharge. J Pediatr. 2008;153(5):616-21, 21 e1-2.

Rochow 2019

Rochow N, Fusch G, Ali A, Bhatia A, So HY, Iskander R, et al. Individualized target fortification of breast milk with protein, carbohydrates, and fat for preterm infants: a double-blind randomised controlled trial. Clin Nutr. 2020;04:031.

Roggero 2012

Roggero P, Gianni ML, Amato O, Liotto N, Morlacchi L, Orsi A, et al. Growth and fat-free mass gain in preterm infants after discharge: a randomized controlled trial. Pediatrics. 2012;130(5):e1215-21.

Tan 2008

Tan MJ, Cooke RW. Improving head growth in very preterm infants--a randomised controlled trial I: neonatal outcomes. Arch Dis Child Fetal Neonatal Ed. 2008;93(5):F337-41.

Zachariassen 2001

Zachariassen G, Faerk J, Grytter C, Esberg BH, Hjelmborg J, Mortensen S, et al. Nutrient enrichment of mother's milk and growth of very preterm infants after hospital discharge. Pediatrics. 2011;127(4):e995-e1003.

Amesz 2010

Amesz EM, Schaafsma A, Cranendonk A, Lafeber HN. Optimal growth and lower fat mass in preterm infants fed a protein-enriched postdischarge formula. J Pediatr Gastroenterol Nutr. 2010;50(2):200-7.

Bellagamba 2016

Bellagamba MP, Carmenati E, D'Ascenzo R, Malatesta M, Spagnoli C, Biagetti C, et al. One extra gram of protein to preterm infants from birth to 1800 g: a single-blinded randomized clinical trial. J Pediatr Gastroenterol Nutr. 2016;62(6):879-84.

Brooke 1985

Brooke OG, Kinsey JM. High energy feeding in small for gestation infants. Arch Dis Child. 1985;60(1):42-6.

Carver 2001

Carver JD, Wu PY, Hall RT, Ziegler EE, Sosa R, Jacobs J, et al. Growth of preterm infants fed nutrient-enriched or term formula after hospital discharge. Pediatrics. 2001;107(4):683-9.

Chan 1994

Chan GM, Borschel MW, Jacobs JR. Effects of human milk or formula feeding on the growth, behavior, and protein status of preterm infants discharged from the newborn intensive care unit. Am J Clin Nutr. 1994;60(5):710-6.

Cooper 1985

Cooper PA, Rothberg AD. Feeding of very-low-birth-weight infants with special formula--continued use beyond 2000 g and effects on growth to 1 year. S Afr Med J. 1985;67(18):716-8.

De Curtis 2002

De Curtis M, Pieltain C, Rigo J. Body composition in preterm infants fed standard term or enriched formula after hospital discharge. Eur J Nutr. 2002;41(4):177-82.

Dogra 2017

Dogra S, Thakur A, Garg P, Kler N. Effect of differential enteral protein on growth and nurodevelopment in infants <1500 g: a randomized controlled trial. J Pediatr. 2017;64(5):e126-e32.

Jeon 2011

Jeon GW, Jung YJ, Koh SY, Lee YK, Kim KA, Shin SM, et al. Preterm infants fed nutrient-enriched formula until 6 months show improved growth and development. Pediatr Int. 2011;53(5):683-8.

Lin 2004

Lin YF, Hsieh KS, Chen YY. Nutrient-enriched versus standard term formula feeding in disproportionately small for gestational age infants. Clin Neonatology. 2004;11(2):36-9.

Lucas 1989

Lucas A, Morley R, Cole TJ, Gore SM, Davis JA, Bamford MF, et al. Early diet in preterm babies and developmental status in infancy. Arch Dis Child. 1989;64(11):1570-8.

Lucas 1990

Lucas A, Morley R, Cole TJ, Gore SM, Lucas PJ, Crowle P, et al. Early diet in preterm babies and developmental status at 18 months. Lancet. 1990;335(8704):1477-81.

Lucas 1992

Lucas A, Bishop NJ, King FJ, Cole TJ. Randomised trial of nutrition for preterm infants after discharge. Arch Dis Child. 1992;67(3):324-7.

O'Connor 2008

O'Connor DL, Khan S, Weishuhn K, Vaughan J, Jefferies A, Campbell DM, et al. Growth and nutrient intakes of human milk-fed preterm infants provided with extra energy and nutrients after hospital discharge. Pediatrics. 2008;121(4):766-76.

Svenningsen 1982

Svenningsen NW, Lindroth M, Lindquist B. A comparative study of varying protein intake in low birthweight infant feeding. Acta Paediatr Suppl. 1982;296:28-31.

Wauben 1998

Wauben IP, Atkinson SA, Shah JK, Paes B. Growth and body composition of preterm infants: influence of nutrient fortification of mother's milk in hospital and breastfeeding post-hospital discharge. Acta Paediatr. 1998;87(7):780-5.

Wheeler 1996

Wheeler RE, Hall RT. Feeding of premature infant formula after hospital discharge of infants weighing less than 1800 grams at birth. J Perinatol. 1996;16(2 Pt 1):111-6.

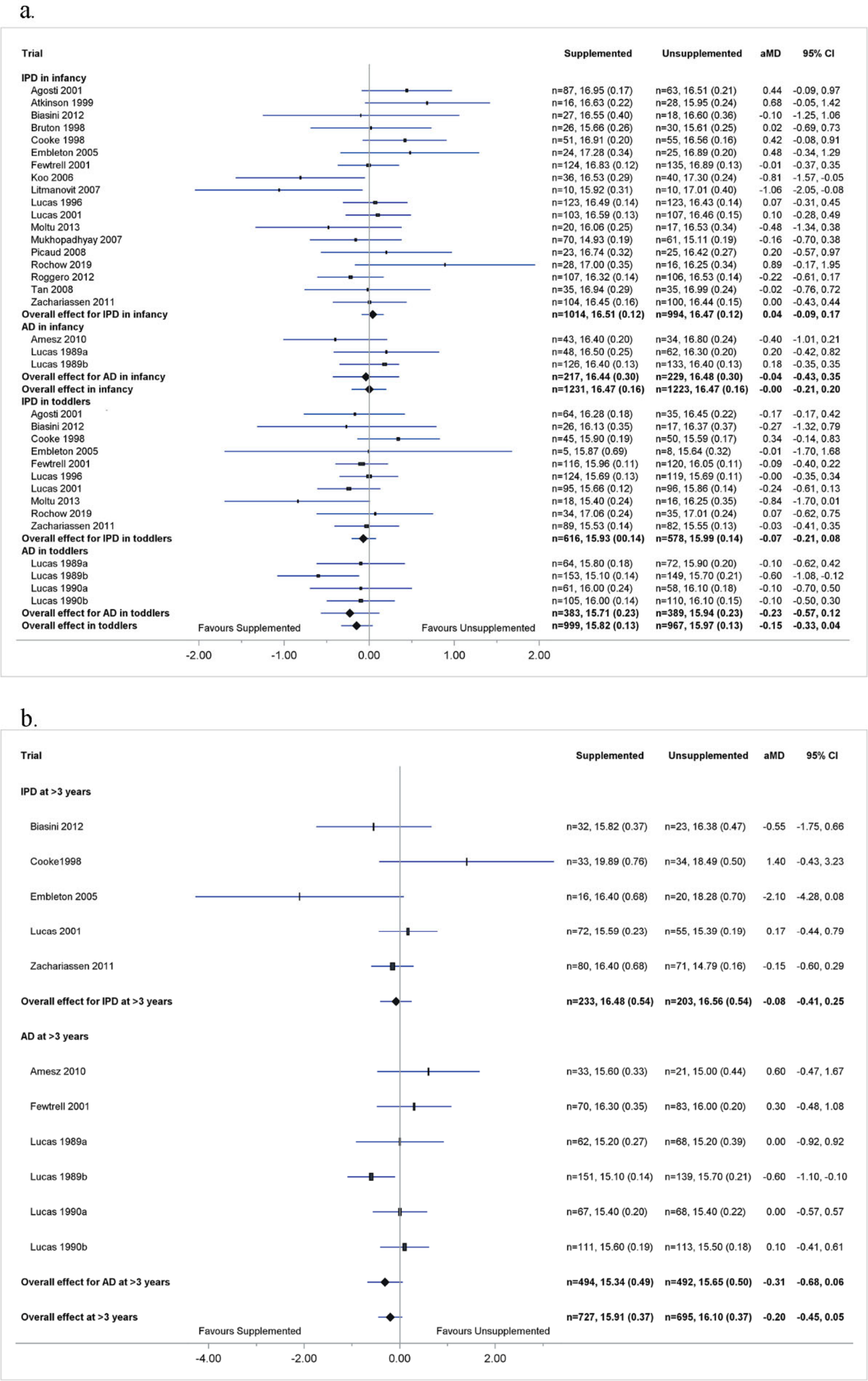

Figure S1. Combined IPD and AD analysis of BMI. a. in infancy and toddlers, b. at >3 years. Data are mean and standard error, with adjusted mean difference (aMD) for treatment effect and 95% confidence intervals (CIs) adjusting for gestational age. The box size of point estimate is proportional to inverse variance. IPD, individual participant data; AD, aggregated data.

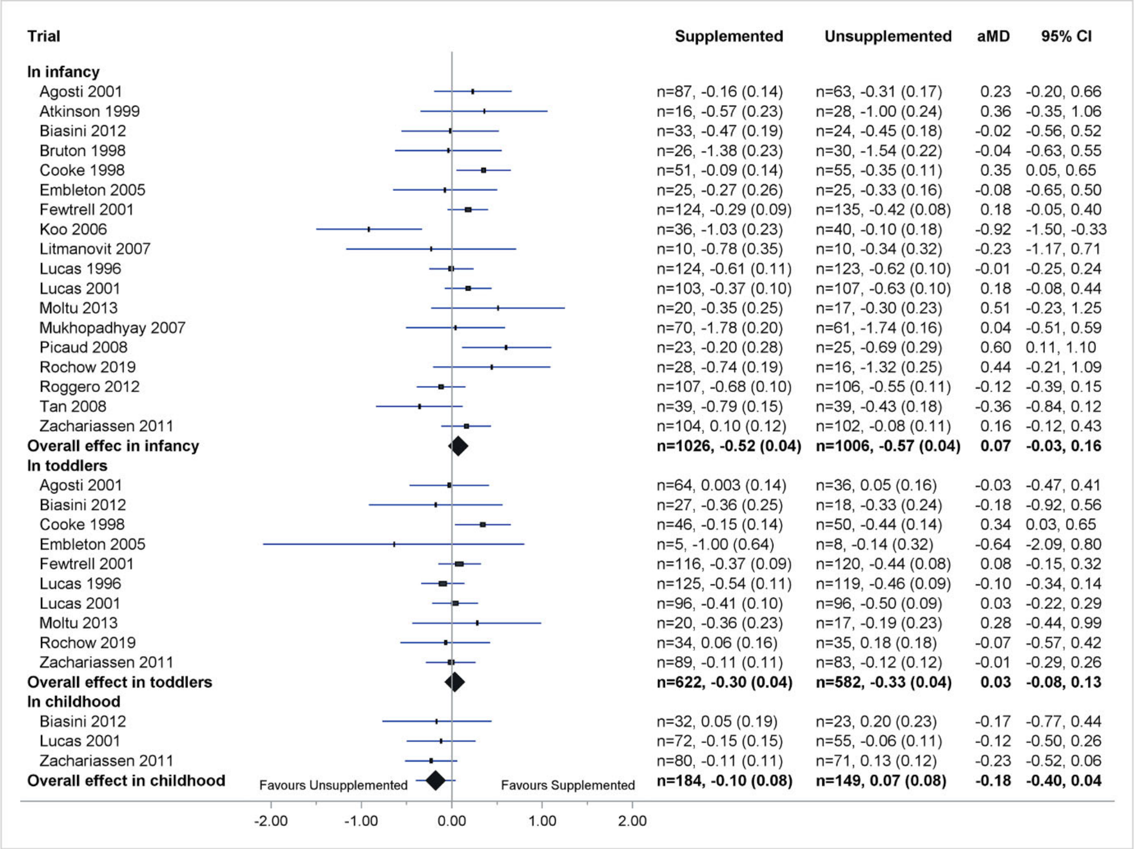

Figure S2. IPD analysis of BMI z-scores. Data are mean and standard error, with adjusted mean difference (aMD) for treatment effect and 95% confidence intervals (CIs) adjusting for sex, gestational age and birthweight z-scores. The box size of point estimate is proportional to inverse variance. P-value for heterogeneity in infancy = 0.36, in toddlers = 0.81, in childhood = 0.64, in adolescence = 0.005, at >3 years = 0.04.

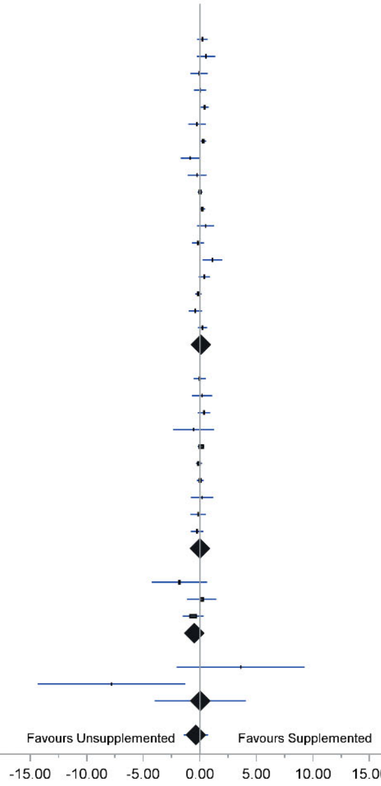

Trial	Supplemented	Unsupplemented	aMD	95% CI
**In infancy**				
Agosti 2001	n=87, 9.25 (0.15)	n=63, 9.00 (0.18)	0.22	-0.24, 0.69
Atkinson 1999	n=16, 8.84 (0.21)	n=28, 8.12 (0.31)	0.54	-0.29, 1.36
Biasini 2012	n=33, 8.49 (0.30)	n=24, 8.54 (0.26)	-0.08	-0.85, 0.69
Bruton 1998	n=26, 8.15 (0.24)	n=30, 7.91 (0.20)	0.01	-0.53, 0.54
Cooke 1998	n=51, 9.23 (0.16)	n=55, 8.83 (0.11)	0.41	0.05, 0.77
Embleton 2005	n=25, 8.47 (0.29)	n=25, 8.68 (0.26)	-0.26	-1.04, 0.52
Fewtrell 2001	n=124, 8.33 (0.09)	n=135, 8.06 (0.10)	0.29	0.04, 0.54
Koo 2006	n=36, 8.04 (0.31)	n=40, 8.92 (0.28)	-0.86	-1.69, -0.03
Litmanovit 2007	n=10, 6.95 (0.31)	n=10, 7.31 (0.22)	-0.25	-1.09, 0.59
Lucas 1996	n=124, 8.06 (0.10)	n=123, 8.01 (0.08)	0.01	-0.21, 0.23
Lucas 2001	n=103, 8.26 (0.12)	n=107, 7.95 (0.10)	0.21	-0.05, 0.48
Moltu 2013	n=20, 9.05 (0.23)	n=17, 9.23 (0.28)	0.50	-0.25, 1.25
Mukhopadhyay 2007	n=70, 7.99 (0.17)	n=61, 7.65 (0.19)	-0.17	-0.70, 0.37
Picaud 2008	n=23, 9.02 (0.41)	n=25, 7.92 (0.35)	1.09	0.22, 1.97
Rochow 2019	n=28, 5.84 (0.16)	n=16, 5.31 (0.18)	0.39	-0.11, 0.89
Roggero 2012	n=107, 8.63 (0.10)	n=106, 8.85 (0.11)	-0.14	-0.41, 0.13
Tan 2008	n=39, 7.39 (0.20)	n=39, 7.75 (0.22)	-0.41	-1.00, 0.19
Zachariassen 2011	n=104, 9.38 (0.15)	n=102, 9.15 (0.18)	0.23	-0.18, 0.65
**Overall effect in infancy**	**n=1026, 8.40 (0.05)**	**n=1006, 8.32 (0.05)**	**0.09**	**-0.02, 0.20**
**In toddlers**				
Agosti 2001	n=64, 10.72 (0.17)	n=36, 10.68 (0.21)	-0.02	-0.56, 0.52
Biasini 2012	n=27, 10.92 (0.30)	n=18, 10.66 (0.30)	0.19	-0.69, 1.08
Cooke 1998	n=46, 11.83 (0.20)	n=50, 11.50 (0.20)	0.36	-0.20, 0.92
Embleton 2005	n=5, 10.32 (0.71)	n=8, 11.21 (0.54)	-0.56	-2.38, 1.26
Fewtrell 2001	n=116, 10.24 (0.11)	n=120, 10.09 (0.11)	0.10	-0.19, 0.39
Lucas 1996	n=125, 10.04 (0.12)	n=119, 10.08 (0.10)	-0.09	-0.37, 0.19
Lucas 2001	n=96, 10.20 (0.13)	n=96, 10.10 (0.12)	0.03	-0.28, 0.33
Moltu 2013	n=20, 11.47 (0.26)	n=17, 11.87 (0.36)	0.19	-0.82, 1.20
Rochow 2019	n=34, 10.95 (0.23)	n=35, 11.13 (0.26)	-0.16	-0.84, 0.53
Zachariassen 2011	n=89, 13.40 (0.21)	n=83, 13.61 (0.22)	-0.25	-0.80, 0.30
**Overall effect in toddlers**	**n=622, 10.92 (0.07)**	**n=582, 10.90 (0.08)**	**0.01**	**-0.14, 0.16**
**In childhood**				
Biasini 2012	n=32, 24.19 (0.80)	n=23, 25.82 (0.91)	-1.81	-4.27, 0.65
Lucas 2001	n=72, 21.01 (0.49)	n=55, 22.76 (0.40)	0.15	-1.15, 1.44
Zachariassen 2011	n=80, 20.49 (0.33)	n=71, 21.08 (0.36)	-0.60	-1.52, 0.32
**Overall effect in childhood**	**n=184, 21.34 (0.29)**	**n=149, 21.69 (0.30)**	**-0.50**	**-1.25, 0.26**
**In adolescence**				
Cooke 1998	n=33, 44.61 (2.04)	n=34, 42.02 (1.74)	3.61	-2.05, 9.27
Embleton 2005	n=16, 29.47 (1.86)	n=20, 33.51 (1.78)	-7.82	-14.36, -1.27
**Overall effect in adolescence**	**n=49, 39.67 (12.65)**	**n=54, 38.87 (10.21)**	**0.03**	**-4.02, 4.07**
**At >3 years**				
**Overall effect at >3 years**	**n=233, 25.20 (10.08)**	**n=203, 26.26 (9.76)**	**-0.36**	**-1.45, 0.72**

Figure S3. IPD analysis of weight. Data are mean and standard error, with adjusted mean difference (aMD) for treatment effect and 95% confidence intervals (CIs) adjusting for sex, gestational age, and birthweight z-scores. The box size of point estimate is proportional to inverse variance. P-value for heterogeneity in infancy = 0.003, in toddlers = 0.84, in childhood = 0.28, in adolescence = 0.07, at >3 years = 0.05.

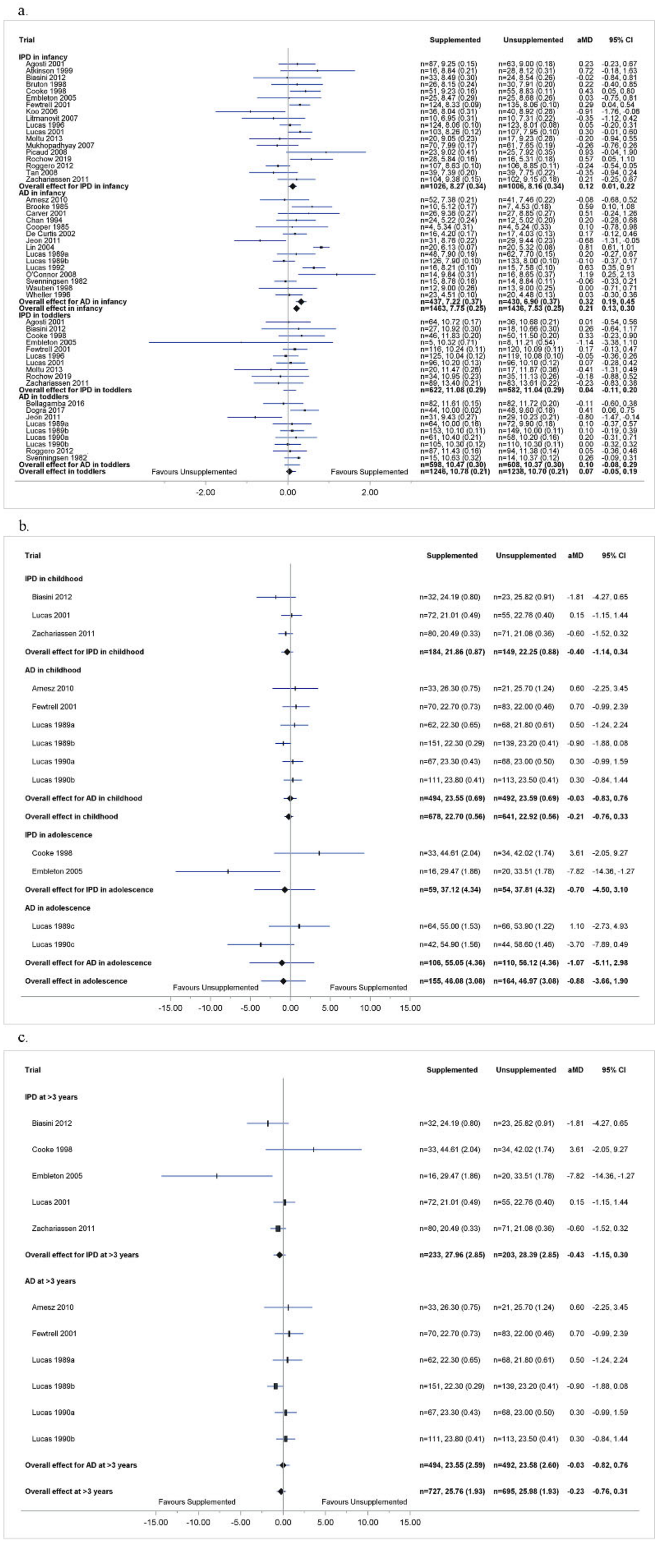

Figure S4. Combined IPD and AD analysis of weight. a. in infancy and in toddlers, b. in childhood and in adolescence, c. at >3 years. Data are mean and standard error, with adjusted mean difference (aMD) for treatment effect and 95% confidence intervals (CIs) adjusting for gestational age. The box size of point estimate is proportional to inverse variance. IPD, individual participant data; AD, aggregated data.

a.

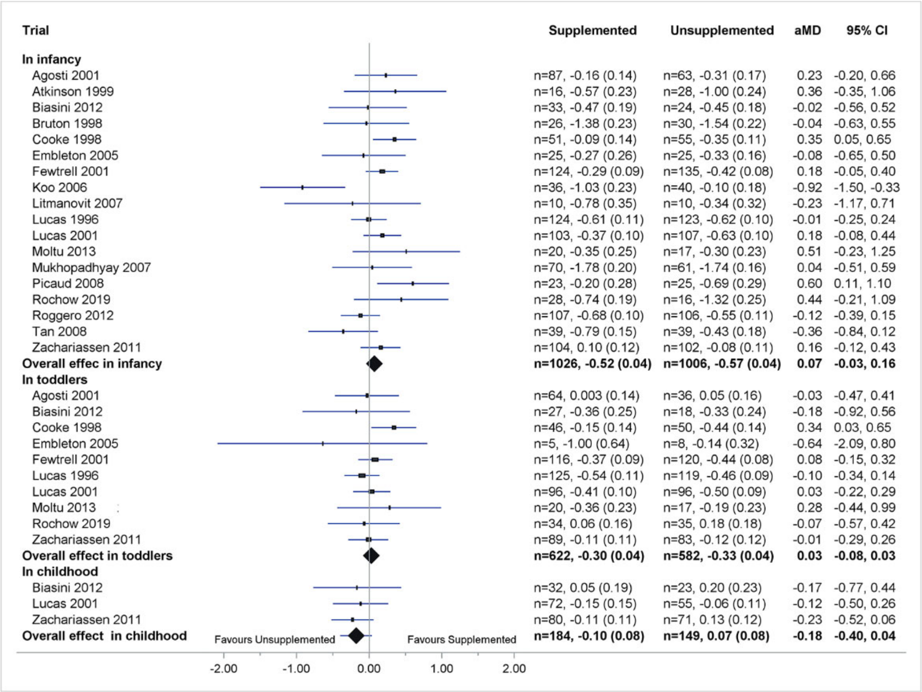

b.

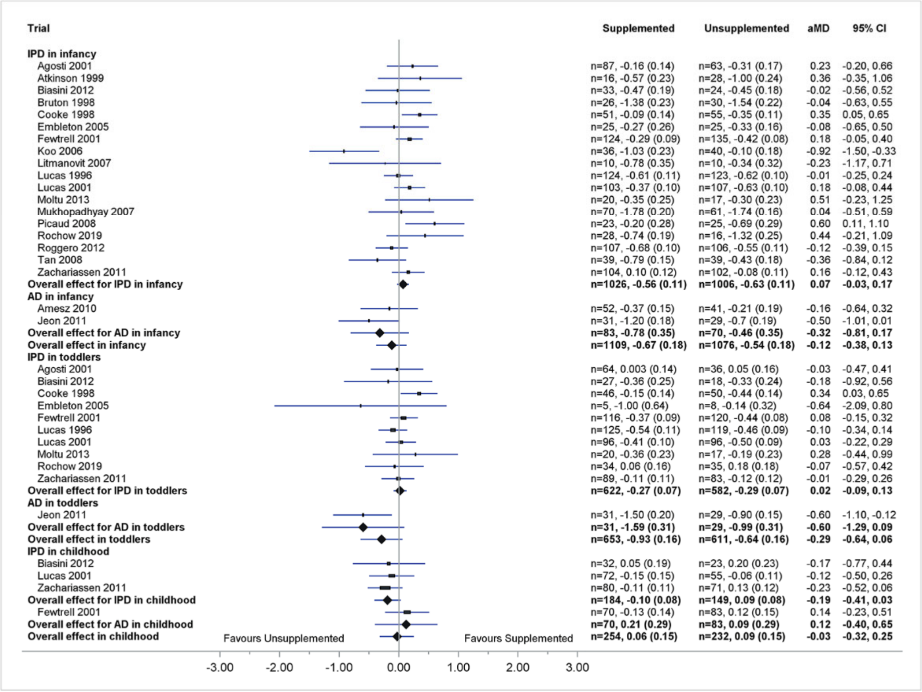

Figure S5. Forest plot of effect of macronutrient supplementation on weight z-scores. a. IPD analysis, b. Combined IPD and AD analysis. Data are mean and standard error, with adjusted mean difference (aMD) for treatment effect and 95% confidence intervals (CIs) adjusted for sex, gestational age and birthweight z-scores. The box size of point estimate is proportional to inverse variance. P-value for heterogeneity of IPD analysis in infancy = 0.01, in toddlers = 0.66, in childhood = 0.92. IPD, individual participant data; AD, aggregated data.

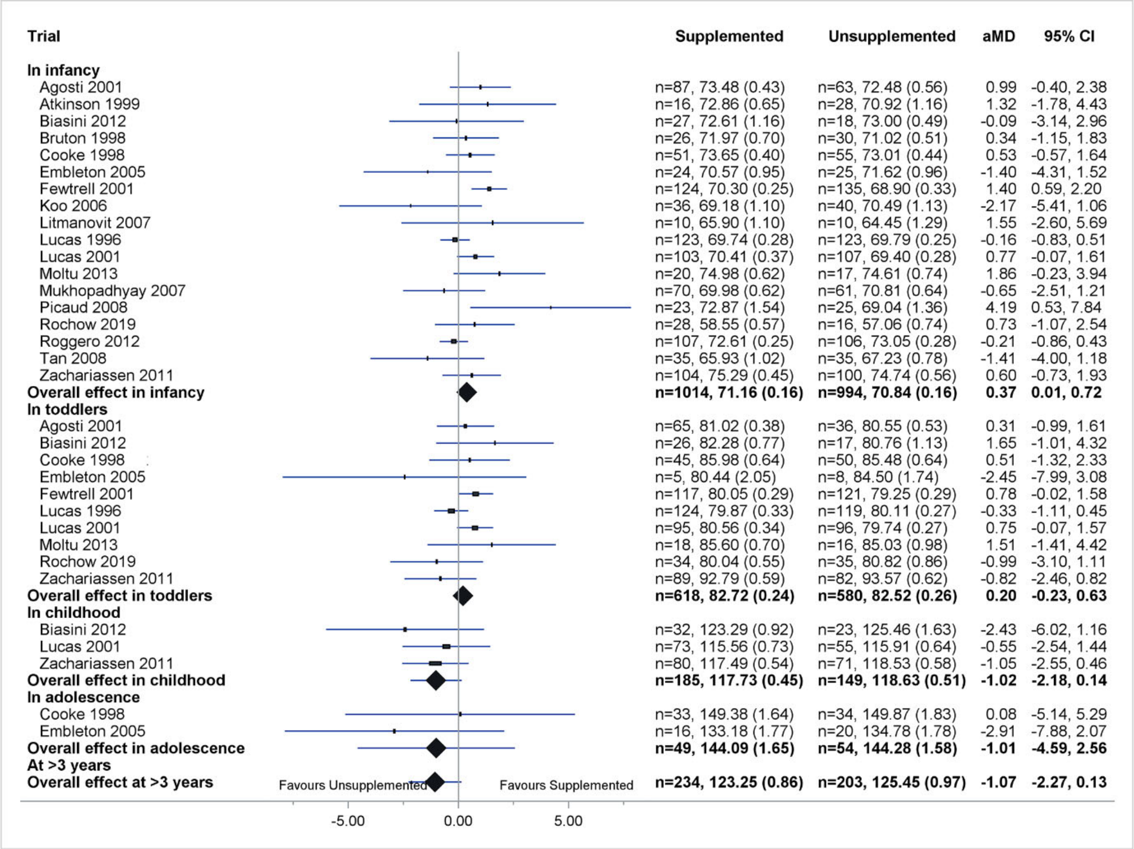

Figure S6. IPD analysis of length/height. Data are mean and standard error, with adjusted mean difference (aMD) for treatment effect and 95% confidence intervals (CIs) adjusted for sex, gestational age, and birthweight z-scores. The box size of point estimate is proportional to inverse variance. P-value for heterogeneity in infancy = 0.003, in toddlers = 0.12, in childhood = 0.58, in adolescence = 0.79, at >3 years = 0.90.

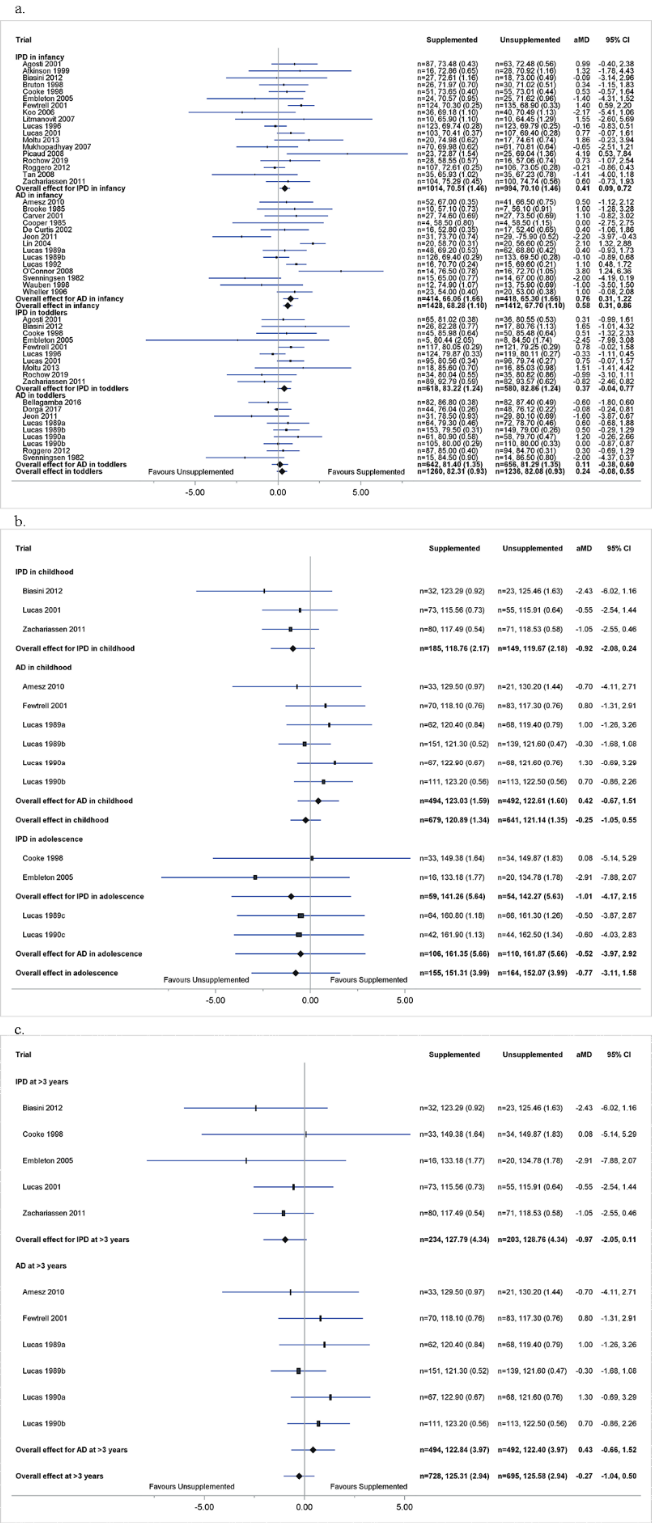

Figure S7. Combined IPD and AD analysis of length/height. a. in infancy and in toddlers, b. in childhood and in adolescence, c. at >3 years. Data are mean and standard error, with adjusted mean difference (aMD) for treatment effect and 95% confidence intervals (CIs) adjusting for gestational age. The box size of point estimate is proportional to inverse variance. IPD, individual participant data; AD, aggregated data.

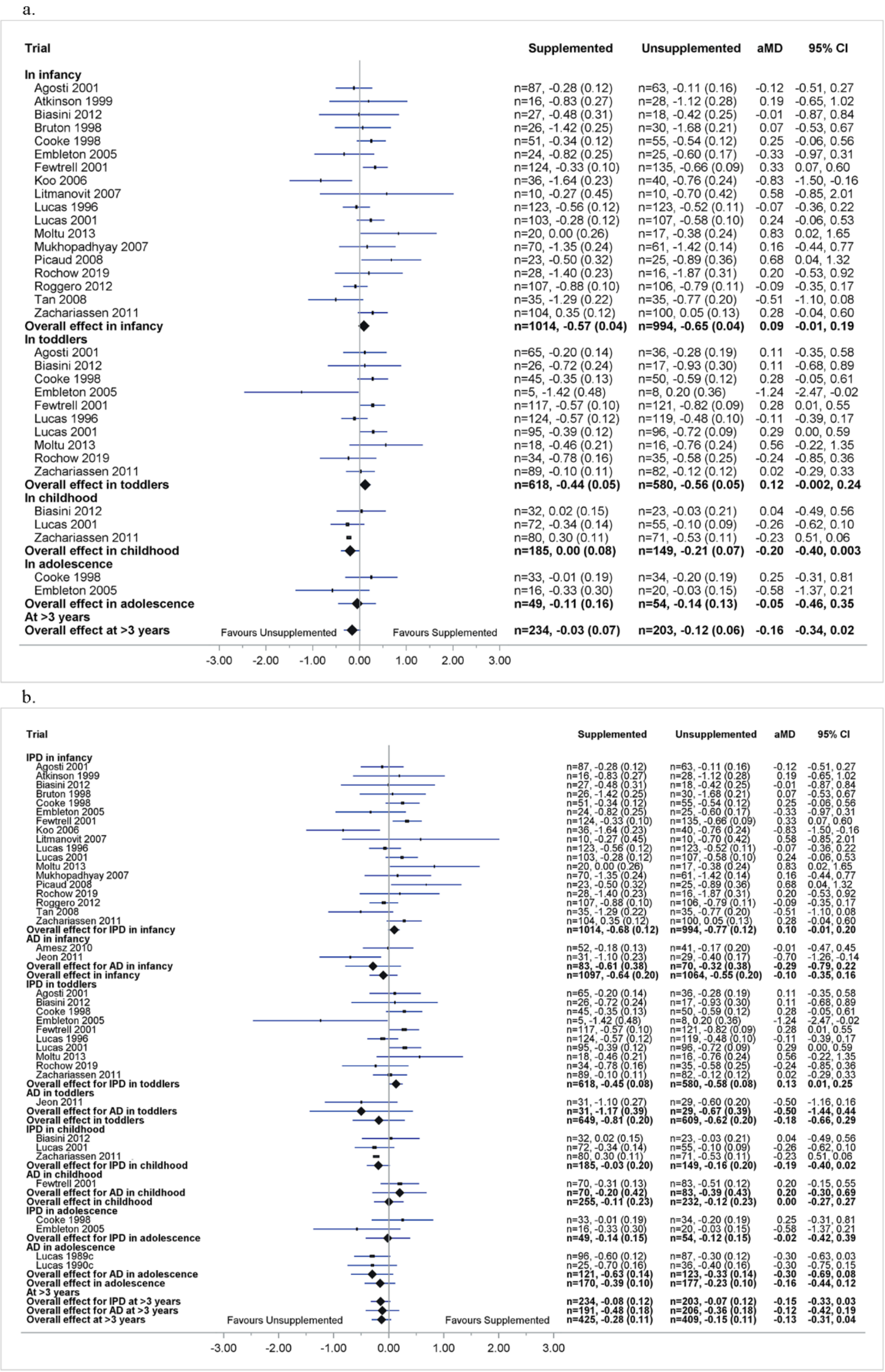

Figure S8. Forest plot of effect of macronutrient supplementation on length/height z-scores. a. IPD analysis, b. Combined IPD and AD analysis. Data are mean and standard error, with adjusted mean difference (aMD) for treatment effect and 95% confidence intervals (CIs) adjusted for sex, gestational age and birthweight z-scores. The box size of point estimate is proportional to inverse variance. P-value for heterogeneity of IPD analysis in infancy = 0.003, in toddlers = 0.04, in childhood = 0.62, in adolescence = 0.12, at >3 years = 0.50. IPD, individual participant data; AD, aggregated data.

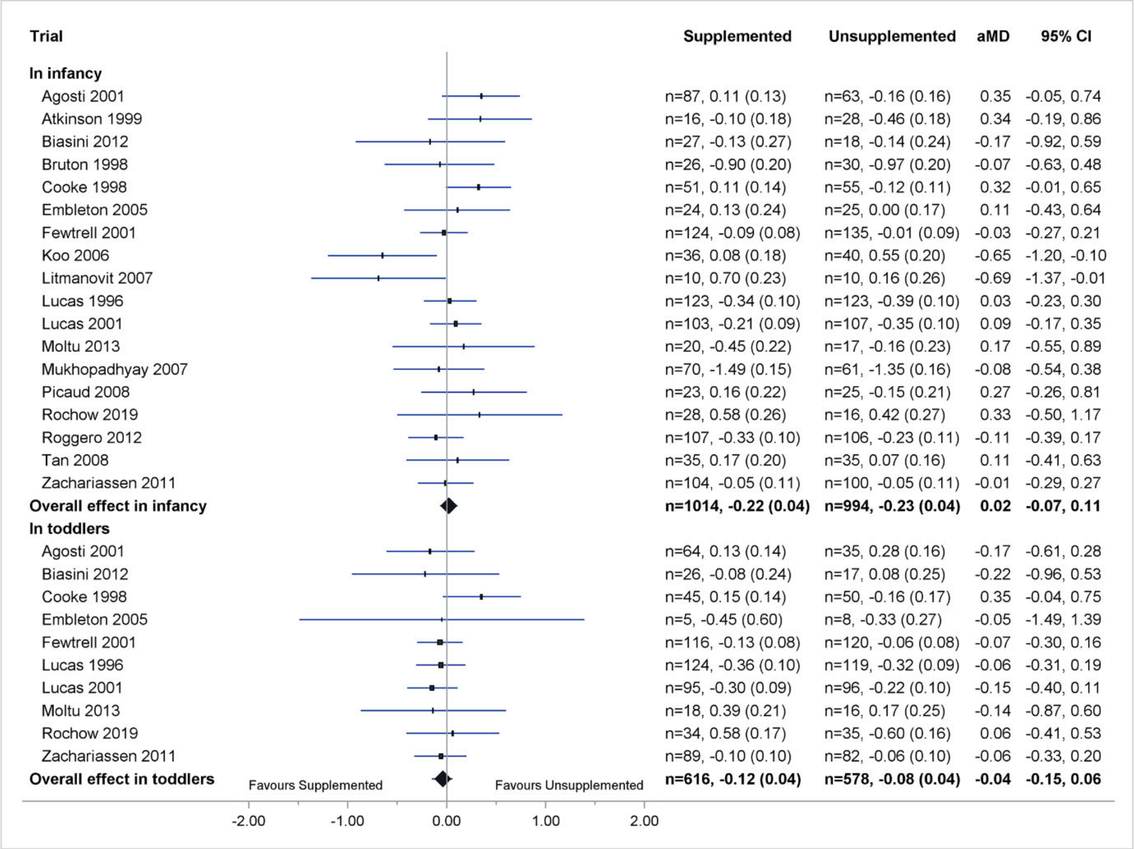

Figure S9. IPD analysis of weigh for length z-scores. Data are mean and standard error, with adjusted mean difference (aMD) for treatment effect and 95% confidence intervals (CIs) adjusting for sex, gestational age and birthweight z-scores. The box size of point estimate is proportional to inverse variance. P-value for heterogeneity in infancy = 0.40, in toddlers = 0.83.

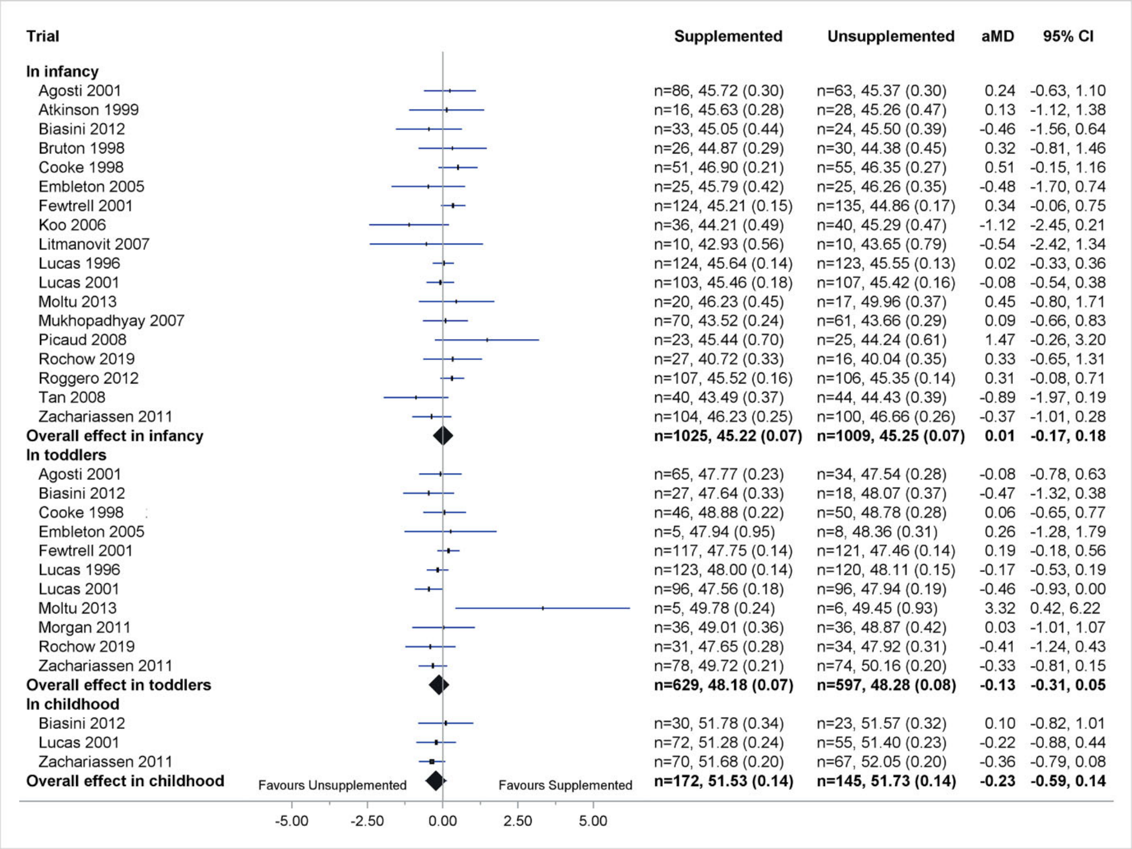

Trial	Supplemented	Unsupplemented	aMD	95% CI
**In infancy**				
Agosti 2001	n=86, 45.72 (0.30)	n=63, 45.37 (0.30)	0.24	-0.63, 1.10
Atkinson 1999	n=16, 45.63 (0.28)	n=28, 45.26 (0.47)	0.13	-1.12, 1.38
Biasini 2012	n=33, 45.05 (0.44)	n=24, 45.50 (0.39)	-0.46	-1.56, 0.64
Bruton 1998	n=26, 44.87 (0.29)	n=30, 44.38 (0.45)	0.32	-0.81, 1.46
Cooke 1998	n=51, 46.90 (0.21)	n=55, 46.35 (0.27)	0.51	-0.15, 1.16
Embleton 2005	n=25, 45.79 (0.42)	n=25, 46.26 (0.35)	-0.48	-1.70, 0.74
Fewtrell 2001	n=124, 45.21 (0.15)	n=135, 44.86 (0.17)	0.34	-0.06, 0.75
Koo 2006	n=36, 44.21 (0.49)	n=40, 45.29 (0.47)	-1.12	-2.45, 0.21
Litmanovit 2007	n=10, 42.93 (0.56)	n=10, 43.65 (0.79)	-0.54	-2.42, 1.34
Lucas 1996	n=124, 45.64 (0.14)	n=123, 45.55 (0.13)	0.02	-0.33, 0.36
Lucas 2001	n=103, 45.46 (0.18)	n=107, 45.42 (0.16)	-0.08	-0.54, 0.38
Moltu 2013	n=20, 46.23 (0.45)	n=17, 49.96 (0.37)	0.45	-0.80, 1.71
Mukhopadhyay 2007	n=70, 43.52 (0.24)	n=61, 43.66 (0.29)	0.09	-0.66, 0.83
Picaud 2008	n=23, 45.44 (0.70)	n=25, 44.24 (0.61)	1.47	-0.26, 3.20
Rochow 2019	n=27, 40.72 (0.33)	n=16, 40.04 (0.35)	0.33	-0.65, 1.31
Roggero 2012	n=107, 45.52 (0.16)	n=106, 45.35 (0.14)	0.31	-0.08, 0.71
Tan 2008	n=40, 43.49 (0.37)	n=44, 44.43 (0.39)	-0.89	-1.97, 0.19
Zachariassen 2011	n=104, 46.23 (0.25)	n=100, 46.66 (0.26)	-0.37	-1.01, 0.28
**Overall effect in infancy**	**n=1025, 45.22 (0.07)**	**n=1009, 45.25 (0.07)**	**0.01**	**-0.17, 0.18**
**In toddlers**				
Agosti 2001	n=65, 47.77 (0.23)	n=34, 47.54 (0.28)	-0.08	-0.78, 0.63
Biasini 2012	n=27, 47.64 (0.33)	n=18, 48.07 (0.37)	-0.47	-1.32, 0.38
Cooke 1998	n=46, 48.88 (0.22)	n=50, 48.78 (0.28)	0.06	-0.65, 0.77
Embleton 2005	n=5, 47.94 (0.95)	n=8, 48.36 (0.31)	0.26	-1.28, 1.79
Fewtrell 2001	n=117, 47.75 (0.14)	n=121, 47.46 (0.14)	0.19	-0.18, 0.56
Lucas 1996	n=123, 48.00 (0.14)	n=120, 48.11 (0.15)	-0.17	-0.53, 0.19
Lucas 2001	n=96, 47.56 (0.18)	n=96, 47.94 (0.19)	-0.46	-0.93, 0.00
Moltu 2013	n=5, 49.78 (0.24)	n=6, 49.45 (0.93)	3.32	0.42, 6.22
Morgan 2011	n=36, 49.01 (0.36)	n=36, 48.87 (0.42)	0.03	-1.01, 1.07
Rochow 2019	n=31, 47.65 (0.28)	n=34, 47.92 (0.31)	-0.41	-1.24, 0.43
Zachariassen 2011	n=78, 49.72 (0.21)	n=74, 50.16 (0.20)	-0.33	-0.81, 0.15
**Overall effect in toddlers**	**n=629, 48.18 (0.07)**	**n=597, 48.28 (0.08)**	**-0.13**	**-0.31, 0.05**
**In childhood**				
Biasini 2012	n=30, 51.78 (0.34)	n=23, 51.57 (0.32)	0.10	-0.82, 1.01
Lucas 2001	n=72, 51.28 (0.24)	n=55, 51.40 (0.23)	-0.22	-0.88, 0.44
Zachariassen 2011	n=70, 51.68 (0.20)	n=67, 52.05 (0.20)	-0.36	-0.79, 0.08
**Overall effect in childhood**	**n=172, 51.53 (0.14)**	**n=145, 51.73 (0.14)**	**-0.23**	**-0.59, 0.14**

Figure S10. IPD analysis of head circumference. Data are mean and standard error, with adjusted mean difference (aMD) for treatment effect and 95% confidence intervals (CIs) adjusting for sex, gestational age, and birthweight z-scores. The box size of point estimate is proportional to inverse variance. P-value for heterogeneity in infancy = 0.09, in toddlers = 0.39, in childhood = 0.60.

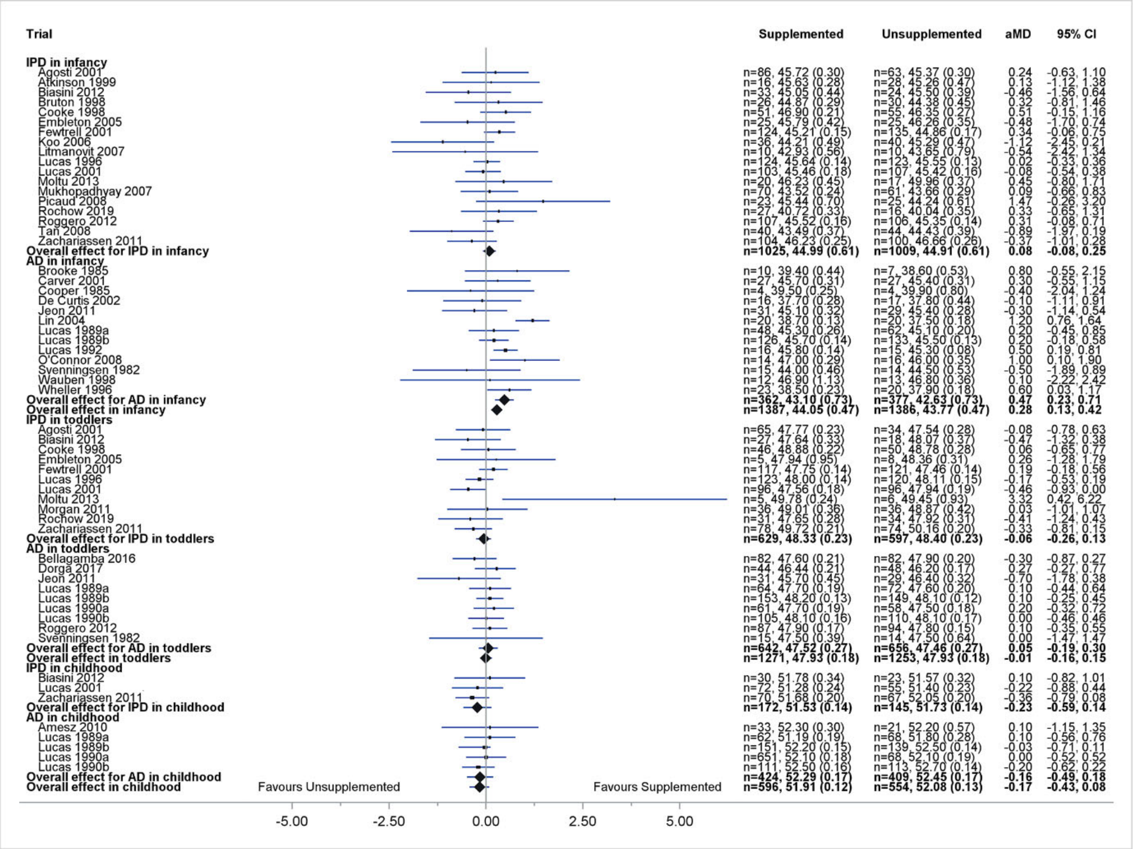

Figure S11. Combined IPD and AD analysis of head circumference. Data are mean and standard error, with adjusted mean difference (aMD) for treatment effect and 95% confidence intervals (CIs) adjusting for gestational age. The box size of point estimate is proportional to inverse variance. IPD, individual participant data; AD, aggregated data.

a.

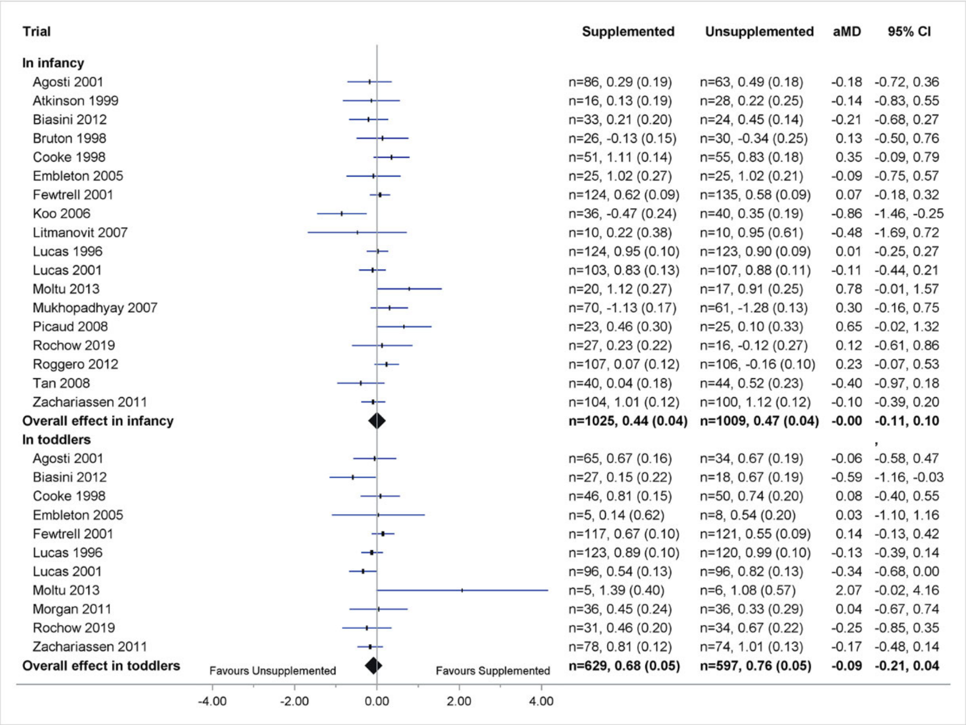

b.

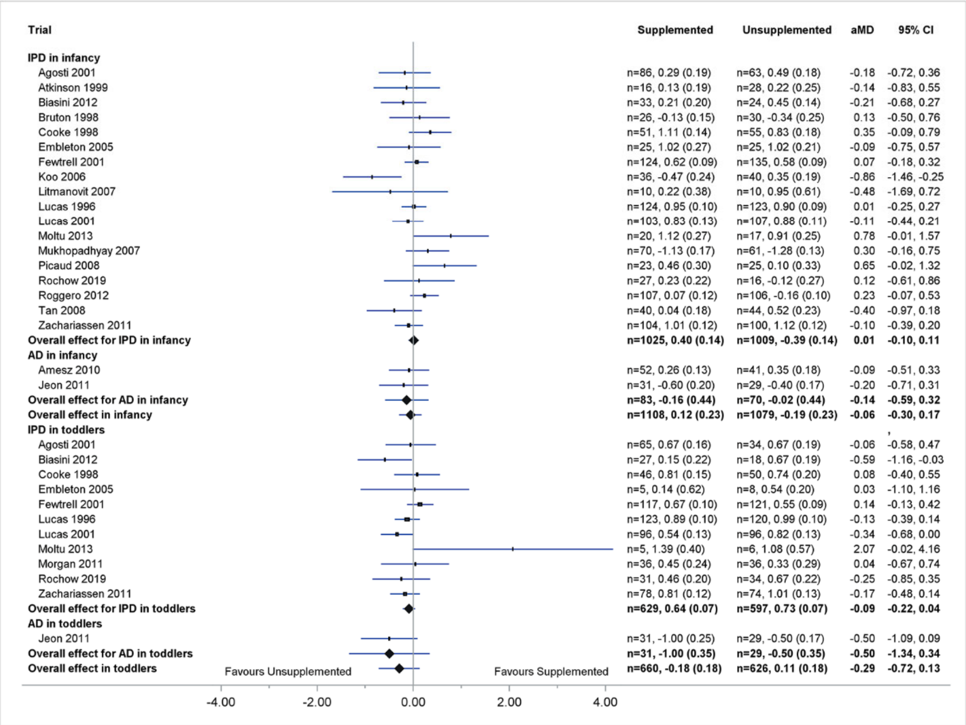

Figure S12. Forest plot of effect of macronutrient supplementation on head circumference z-scores. a. IPD analysis, b. Combined IPD and AD analysis. Data are mean and standard error, with adjusted mean difference (aMD) for treatment effect and 95% confidence intervals (CIs) adjusted for sex, gestational age and birthweight z-scores. The box size of point estimate is proportional to inverse variance. P-value for heterogeneity of IPD analysis in infancy = 0.10, in toddlers = 0.32. IPD, individual participant data; AD, aggregated data.

a.

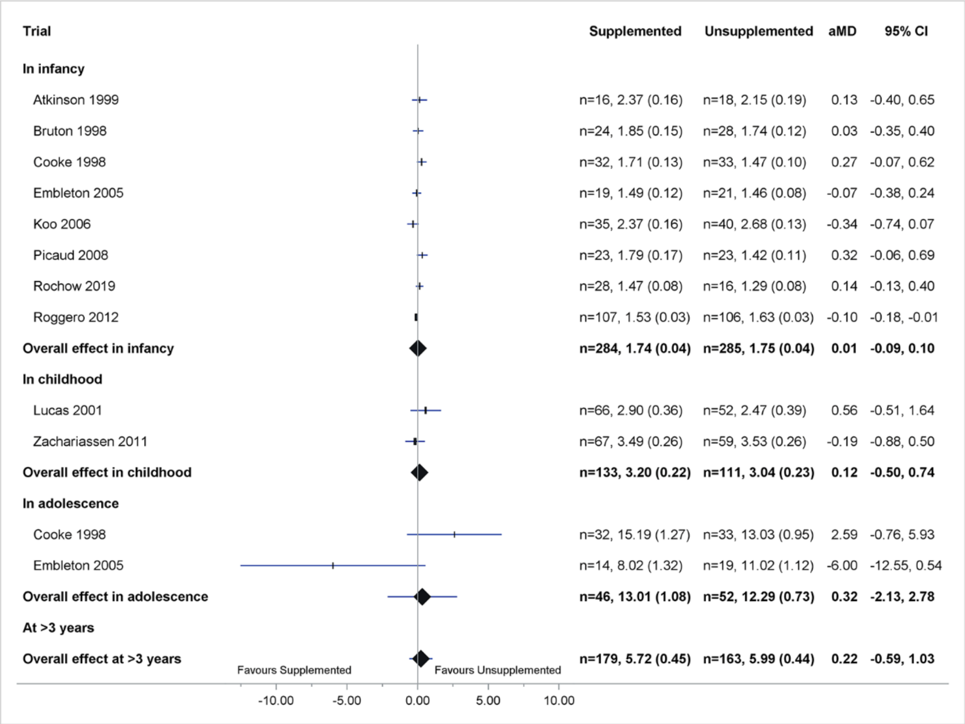

b.

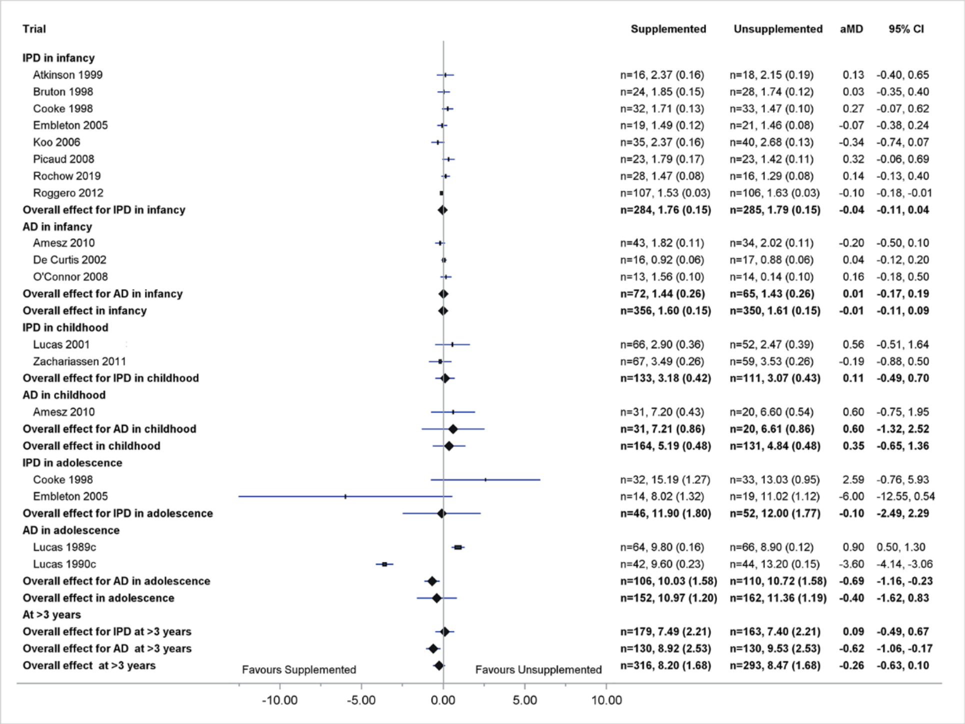

Figure S13. Forest plot of effect of macronutrient supplementation on fat mass. a. IPD analysis, b. Combined IPD and AD analysis. Data are mean and standard error, with adjusted mean difference (aMD) for treatment effect and 95% confidence intervals (CIs) adjusted for sex, gestational age and birthweight z-scores. The box size of point estimate is proportional to inverse variance within each age group. P-value for heterogeneity of IPD analysis in infancy = 0.03, in childhood = 0.43, in adolescence = 0.02, at >3 years = 0.02. IPD, individual participant data; AD, aggregated data.

a.

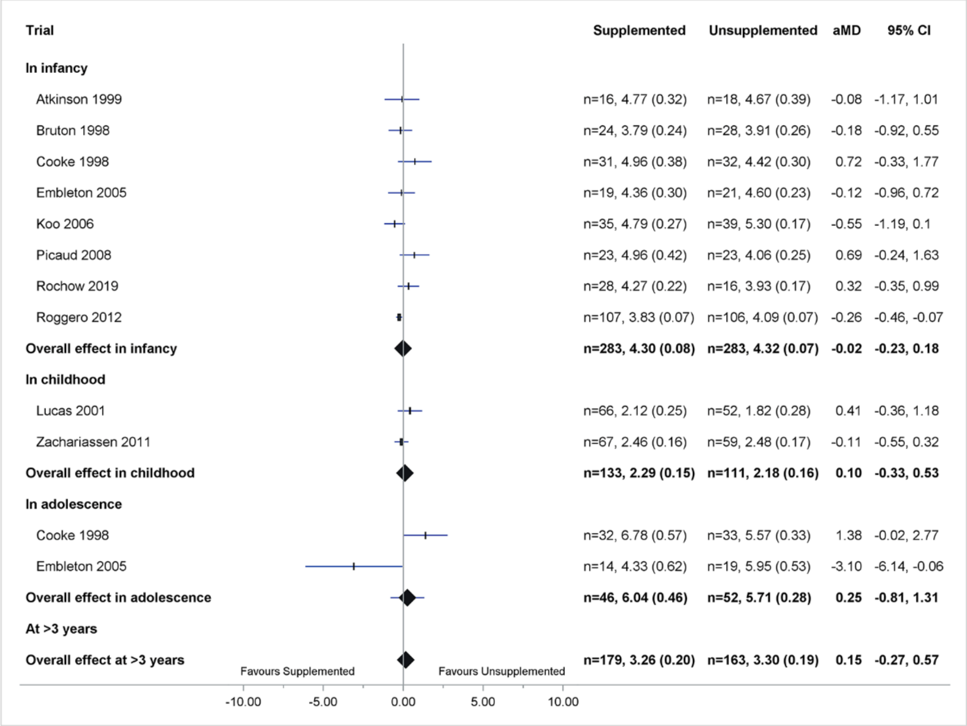

b.

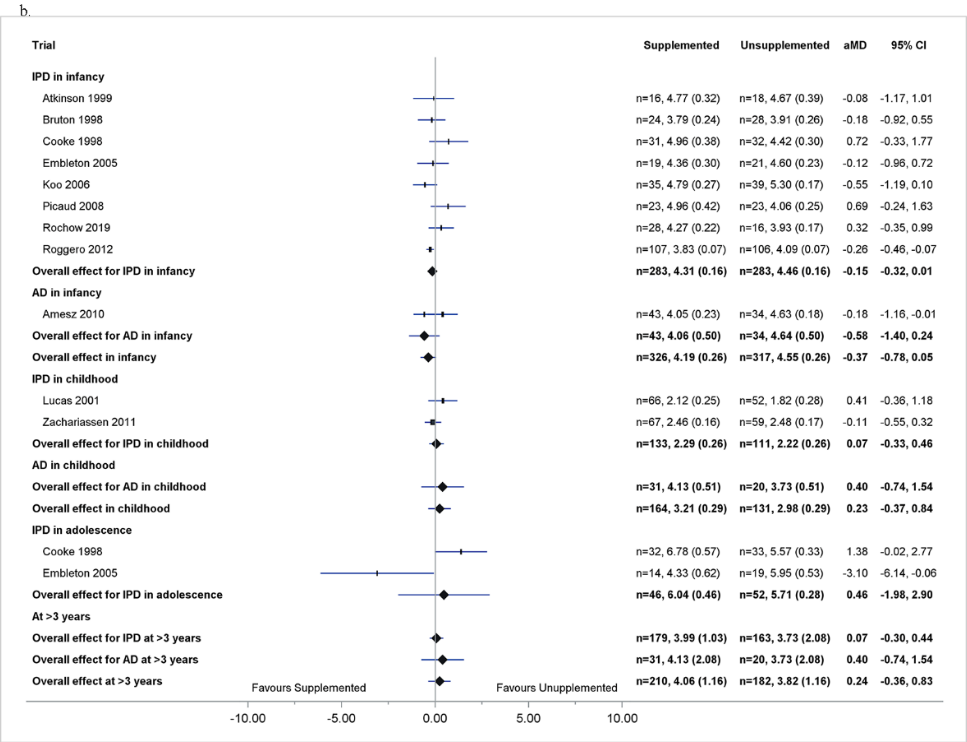

Figure S14. Forest plot of effect of macronutrient supplementation on fat mass index. a. IPD analysis, b. Combined IPD and AD analysis. Data are mean and standard error, with adjusted mean difference (aMD) for treatment effect and 95% confidence intervals (CIs) adjusted for sex, gestational age and birthweight z-scores. The box size of point estimate is proportional to inverse variance. P-value for heterogeneity of IPD analysis in infancy = 0.84, in childhood = 0.42, in adolescence = 0.01, at >3 years = 0.01. IPD, individual participant data; AD, aggregated data.

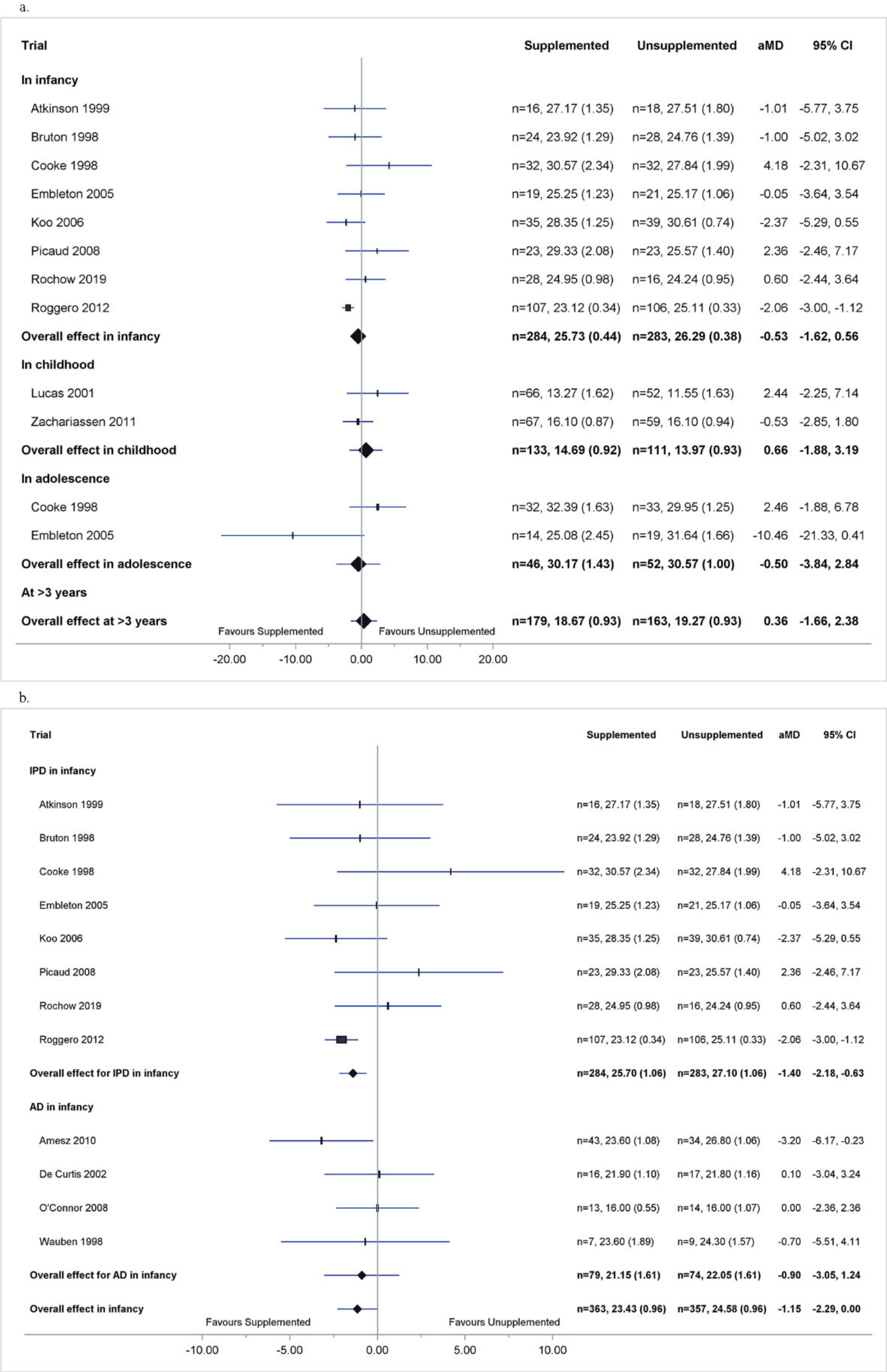

Figure S15. Forest plot of effect of macronutrient supplementation on percent fat mass. a. IPD analysis, b. Combined IPD and AD analysis. Data are mean and standard error, with adjusted mean difference (aMD) for treatment effect and 95% confidence intervals (CIs) adjusted for sex, gestational age and birthweight z-scores. The box size of point estimate is proportional to inverse variance within each age group. P-value for heterogeneity of IPD analysis in infancy = 0.07, in childhood = 0.43, in adolescence = 0.02, at >3 years = 0.23. IPD, individual participant data; AD, aggregated data.

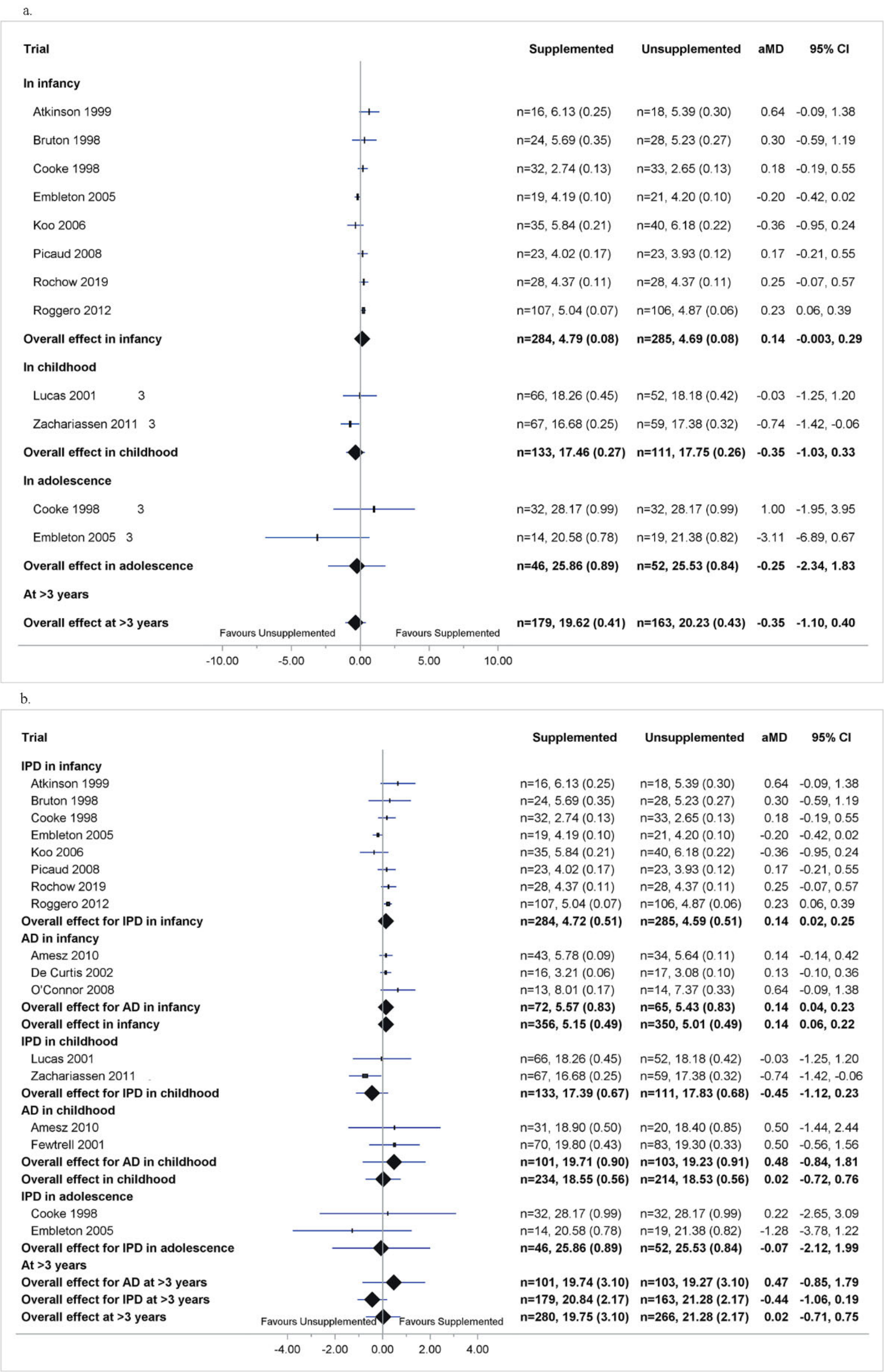

Figure S16. Forest plot of effect of macronutrient supplementation on lean mass. a. IPD analysis, b. Combined IPD and AD analysis. Data are mean and standard error, with adjusted mean difference (aMD) for treatment effect and 95% confidence intervals (CIs) adjusted for sex, gestational age and birthweight z-scores. The box size of point estimate is proportional to inverse variance within each age group. P-value for heterogeneity of IPD analysis in infancy = 0.12, in childhood = 0.40, in adolescence = 0.36, at >3 years = 0.632. IPD, individual participant data; AD, aggregated data.

a.

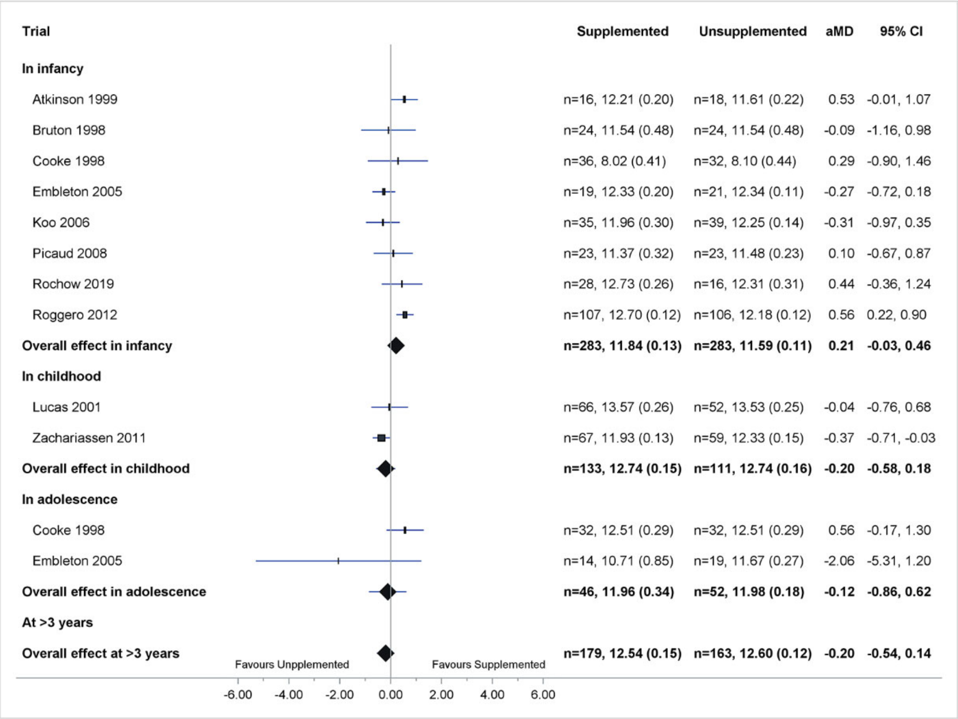

b.

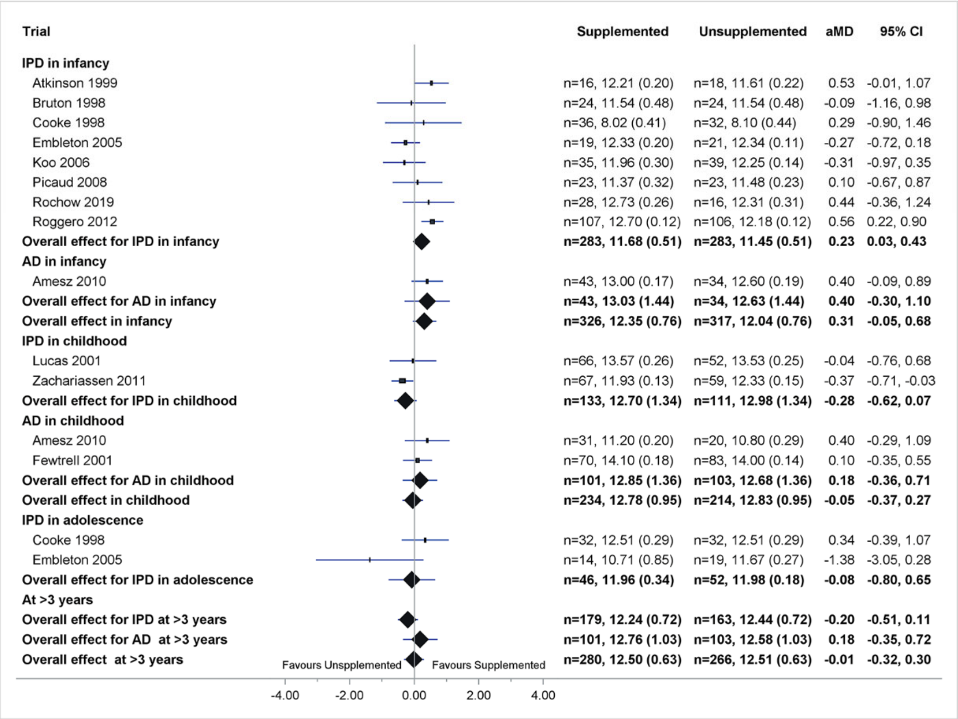

Figure S17. Forest plot of effect of macronutrient supplementation on lean mass index. a. IPD analysis, b. Combined IPD and AD analysis. Data are mean and standard error, with adjusted mean difference (aMD) for treatment effect and 95% confidence intervals (CIs) adjusted for sex, gestational age and birthweight z-scores. The box size of point estimate is proportional to inverse variance. P-value for heterogeneity of IPD analysis in infancy = 0.37, in childhood = 0.41, in adolescence = 0.04, at >3 years = 0.11. IPD, individual participant data; AD, aggregated data.

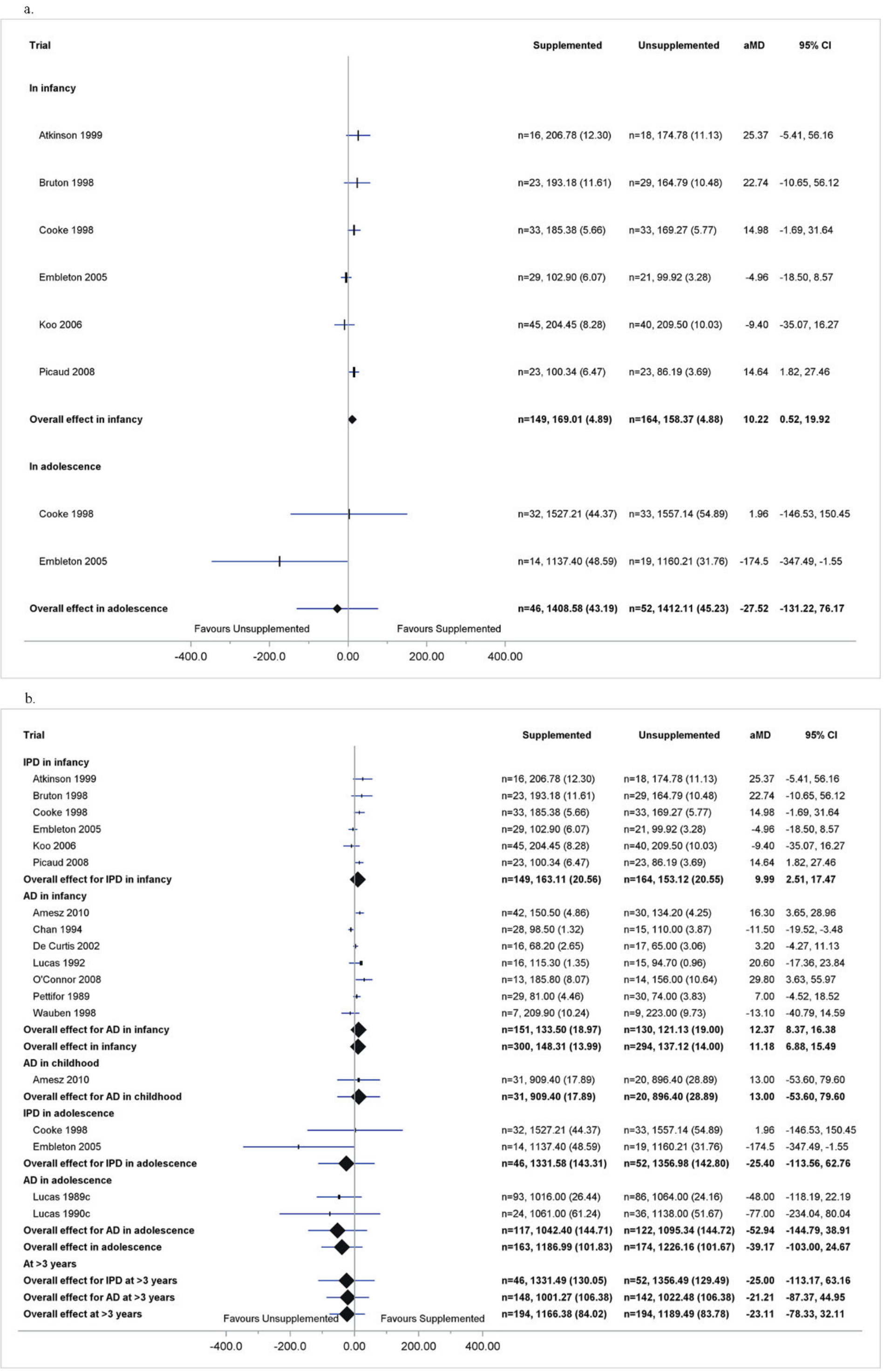

Figure S18. Forest plot of effect of macronutrient supplementation on bone mineral content. a. IPD analysis, b. Combined IPD and AD analysis. Data are mean and standard error, with adjusted mean difference (aMD) for treatment effect and 95% confidence intervals (CIs) adjusted for sex, gestational age and birthweight z-scores. The box size of point estimate is proportional to inverse variance within each age group. P-value for heterogeneity of IPD analysis in infancy = 0.21, in adolescence = 0.91. IPD, individual participant data; AD, aggregated data.

a.

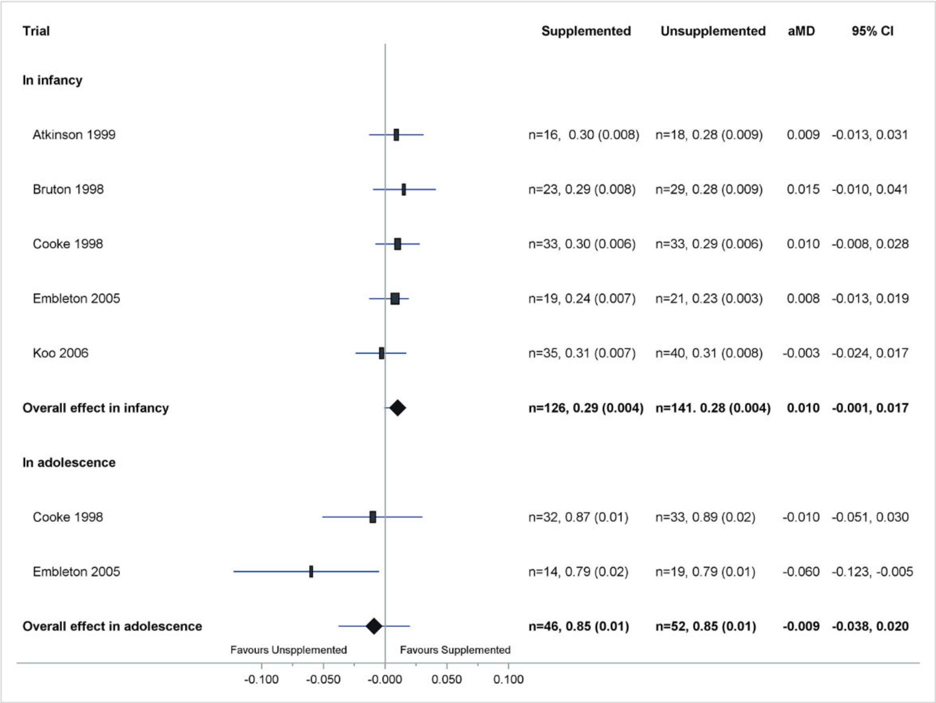

b.

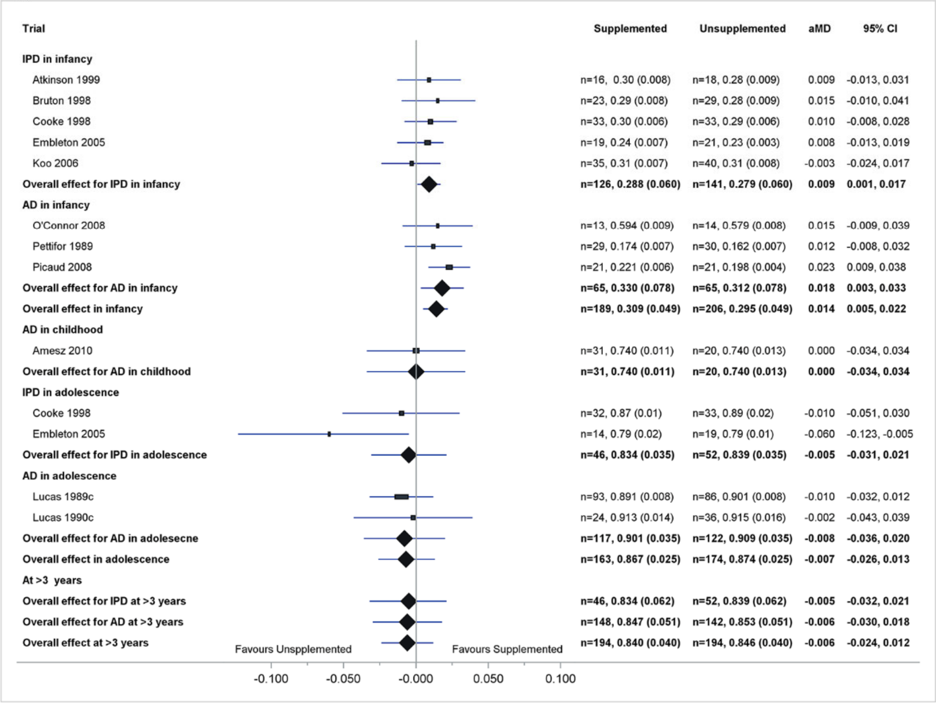

Figure S19. Forest plot of effect of macronutrient supplementation on bone mineral density. a. IPD analysis, b. Combined IPD and AD analysis. Data are mean and standard error, with adjusted mean difference (aMD) for treatment effect and 95% confidence intervals (CIs) adjusted for sex, gestational age and birthweight z-scores. The box size of point estimate is proportional to inverse variance within each age group. P-value for heterogeneity of IPD analysis in infancy = 0.74, in adolescence = 0.91. IPD, individual participant data; AD, aggregated data.

Table S1. Risk of bias within studies.

Study	Randomisation[1]	Concealment[2]	Performance[3]	Detection[4]	Attrition[5]	Reporting[6]
**Studies with IPD**						
Agosti 2003	Unclear	Unclear	Low	Low	Low	Low
Atkinson 1999	Low	Low	Low	Low	Low	Low
Biasini 2012	High	High	High	Low	Low	Low
Brunton 1998	Low	Low	Low	High	Low	Low
Cooke 1998	Low	Low	Low	Low	Low	Low
Embleton 2005	Low	Low	Low	Low	High	Low
Fewtrell 2001	Low	Low	Low	Low	Low	Low
Koo 2006	Low	Low	Low	Low	Low	Low
Litmanovitz 2007	High	High	Low	Low	Low	Low
Lucas 1996	Low	Low	High	Low	Low	Low
Lucas 2001	Low	Low	Low	Low	Low	Low
Moltu 2013	Low	Low	High	High	Low	Low
Morgan 201	Low	Low	Low	Low	Unclear	Low
Mukhopadhyay 2007	Low	Low	Unclear	Unclear	Low	Low
Picaud 2008	Low	Low	Low	Low	Low	Low
Rochow 2019	Low	Low	Low	Low	Low	Low
Roggero 2012	Low	Low	High	Low	Low	Low
Tan 2008	Low	Low	High	High	Low	Low
Zachariassen 2001	Low	Low	High	High	Low	Low
**Studies with AD**						
Amesz 2010	Low	Low	Low	Unclear	High	Low
Bellagamba 2016	Low	Unclear	Low	Low	Unclear	Low
Brooke 1985	Unclear	Unclear	Low	Unclear	Low	Low
Carver 2001	Low	Unclear	Unclear	Unclear	High	Unclear
Chan 1994	Unclear	Unclear	Unclear	Unclear	Low	Low
Cooper 1985	Unclear	Unclear	Unclear	Unclear	Low	High
De Curtis 2002	Unclear	Unclear	Unclear	Unclear	Low	Low
Dogra 2017	Low	Low	Low	Low	High	Low
Jeon 2011	Unclear	Unclear	Unclear	Unclear	High	Unclear
Lin 2004	Unclear	Unclear	Unclear	Unclear	Low	Low
Lucas 1989	Low	Low	High	Low	High	Low
Lucas 1990	Low	Low	High	Low	High	Low
Lucas 1992	Unclear	Unclear	Unclear	Unclear	Low	Low
O'Connor 2008	Low	Low	Unclear	Unclear	High	Low
Svenningsen 1982	Unclear	Unclear	Unclear	Unclear	Low	High
Wauben 1998	Low	Unclear	High	High	High	Low
Wheeler 1996	Low	Unclear	Unclear	Unclear	Low	Low

**Study**	**Randomisation[1]**	**Concealment[2]**	**Performance[3]**	**Detection[4]**	**Attrition[5]**	**Reporting[6]**
[1]Random sequence generation. [2]Allocation concealment. [3]Blinding of participants and personnel. [4]Blinding of outcome assessment. [5]Incomplete outcome data. [6]Selective reporting.<br>We used IPD not the published data for studies with IPD, so the risk of reporting bias is low for all the studies with IPD.<br>IPD: individual participant data; AD: aggregated data.						

Table S2. Subgroup analyses of infant sex.

Outcome	Subgroup	No. of trials	No. of participants	aMD (95% CI)	P for overall effect	P for heterogeneity	P for subgroup interaction
**BMI**							
BMI in childhood (kg/m^2)	Boys	3 trials	155	-0.32 (-0.85, 0.21)	0.23	0.17	0.25
	Girls	3 trials	178	0.10 (-0.39, 0.59)	0.70	0.74	
BMI in infancy (kg/m^2)	Boys	18 trials	1036	0.09 (-0.09, 0.27)	0.32	0.18	0.36
	Girls	18 trials	972	-0.03 (-0.21, 0.15)	0.75	0.76	
BMI in toddlers (kg/m^2)	Boys	10 trials	592	-0.10 (-0.30, 0.11)	0.35	0.09	0.72
	Girls	10 trials	602	-0.05 (-0.25, 0.16)	0.66	0.67	
BMI in adolescence (kg/m^2)	Boys	2 trials	50	0.45 (-1.59, 2.48)	0.66	0.08	0.68
	Girls	2 trials	53	-0.15 (-2.13, 1.83)	0.88	0.06	
BMI at >3 years (kg/m^2)	Boys	5 trials	205	-0.12 (-0.74, 0.50)	0.71	0.01	0.73
	Girls	5 trials	231	0.03 (-0.55, 0.61)	0.92	0.16	
BMI z-scores in infancy	Boys	18 trials	1036	0.07 (-0.06, 0.20)	0.29	0.18	0.30
	Girls	18 trials	972	-0.03 (-0.16, 0.10)	0.67	0.76	
BMI z-scores in toddlers	Boys	10 trials	592	-0.10 (-0.25, 0.05)	0.20	0.66	0.63
	Girls	10 trials	602	-0.05 (-0.20, 0.11)	0.54	0.09	
BMI z-scores in childhood	Boys	3 trials	155	-0.25 (-0.58, 0.09)	0.15	0.28	0.21
	Girls	3 trials	178	0.05 (-0.27, 0.36)	0.77	0.82	
BMI z-scores in adolescence	Boys	2 trials	50	0.01 (-0.74, 0.75)	0.99	0.10	0.76
	Girls	2 trials	53	-0.16 (-0.88, 0.56)	0.66	0.02	
BMI z-scores at >3 years	Boys	5 trials	205	-0.18 (-0.49, 0.13)	0.26	0.09	0.42
	Girls	5 trials	231	-0.01 (-0.29, 0.29)	0.98	0.24	
**Weight**							
Weight in infancy (kg)	Boys	18 trials	1043	0.12 (-0.03, 0.26)	0.13	0.01	0.63
	Girls	18 trials	989	0.06 (-0.09, 0.22)	0.42	0.06	
Weight in toddlers (kg)	Boys	10 trials	599	-0.01 (-0.22, 0.20)	0.94	0.28	0.86
	Girls	10 trials	605	0.02 (-0.19, 0.23)	0.85	0.53	
Weight in childhood (kg)	Boys	3 trials	155	-0.88 (-1.98, 0.23)	0.12	0.20	0.32
	Girls	3 trials	178	-0.12 (-1.15, 0.91)	0.82	0.58	
	Boys	2 trials	50	0.69 (-5.09, 6.48)	0.81	0.12	0.74

Outcome	Subgroup	No. of trials	No. of participants	aMD (95% CI)	P for overall effect	P for heterogeneity	P for subgroup interaction
Weight in adolescence (kg)	Girls	2 trials	53	-0.64 (-6.28, 5.01)	0.82	0.38	
Weight at >3 years (kg)	Boys	5 trials	205	-0.50 (-2.09, 1.08)	0.53	0.04	0.80
	Girls	5 trials	231	-0.22 (-1.71, 1.26)	0.77	0.46	
Weight z-scores in infancy	Boys	18 trials	1043	0.11 (-0.02, 0.23)	0.11	0.006	0.44
	Girls	18 trials	989	0.03 (-0.10, 0.17)	0.63	0.12	
Weight z-scores in toddlers	Boys	10 trials	599	0.06 (-0.09, 0.22)	0.41	0.25	0.47
	Girls	10 trials	605	-0.01 (-0.17, 0.14)	0.86	0.10	
Weight z-scores in childhood	Boys	3 trials	155	-0.88 (01.98, 0.23)	0.12	0.47	0.44
	Girls	3 trials	178	-0.12 (-1.15, 0.91)	0.82	0.74	
**Length/height**							
Length in infancy (cm)	Boys	18 trials	1036	0.28 (-0.21, 0.77)	0.26	0.07	0.64
	Girls	18 trials	972	0.45 (-0.06, 0.95)	0.08	0.04	
Height in toddlers (cm)	Boys	10 trials	595	0.24 (-0.37, 0.84)	0.44	0.41	0.87
	Girls	10 trials	603	0.16 (-0.44, 0.76)	0.59	0.25	
Height in childhood (cm)	Boys	3 trials	156	-1.25 (-2.94, 0.45)	0.15	0.75	0.70
	Girls	3 trials	178	-0.80 (-2.38, 0.78)	0.32	0.73	
Height in adolescence (cm)	Boys	2 trials	50	-1.62 (-6.73, 3.49)	0.53	0.87	0.74
	Girls	2 trials	53	-0.41 (-5.39, 4.58)	0.87	0.76	
Height at >3 years (cm)	Boys	5 trials	206	-1.45 (-3.19, 0.30)	0.10	0.93	0.54
	Girls	5 trials	231	-0.70 (-2.35, 0.95)	0.40	0.94	
Length z-scores in infancy	Boys	18 trials	1036	0.09 (-0.05, 0.24)	0.21	0.01	0.98
	Girls	18 trials	972	0.09 (-0.06, 0.24)	0.24	0.12	
Height z-scores in toddlers	Boys	10 trials	595	0.20 (0.02, 0.37)	0.03	0.21	0.21
	Girls	10 trials	603	0.04 (-0.13, 0.21)	0.64	0.05	
Height z-scores in childhood	Boys	3 trials	156	-0.21 (-0.51, 0.08)	0.16	0.65	0.91
	Girls	3 trials	178	-0.19 (-0.47, 0.09)	0.18	0.74	
Height z-scores in adolescence	Boys	2 trials	50	0.07 (-0.52, 0.64)	0.83	0.27	0.57
	Girls	2 trials	53	-0.17 (-0.73, 0.39)	0.55	0.63	
Height z-scores at >3 years	Boys	5 trials	206	-0.14 (-0.40, 0.12)	0.29	0.48	0.84
	Girls	5 trials	231	-0.18 (-0.43, 0.07)	0.16	0.85	
Weight for length z-scores in infancy	Boys	18 trials	1036	0.06 (-0.07, 0.18)	0.38	0.17	0.42
	Girls	18 trials	972	-0.02 (-0.15, 0.11)	0.78	0.62	
	Boys	10 trials	591	-0.06 (-0.21, 0.09)	0.44	0.16	0.76

Outcome	Subgroup	No. of trials	No. of participants	aMD (95% CI)	P for overall effect	P for heterogeneity	P for subgroup interaction
Weight for length z-scores in toddlers	Girls	10 trials	602	-0.03 (-0.18, 0.13)	0.73	0.51	
**HC**							
HC in infancy (cm)	Boys	18 trials	1045	0.01 (-0.23, 0.26)	0.91	0.05	0.94
	Girls	18 trials	989	-0.00 (-0.25, 0.25)	0.99	0.12	
HC in toddlers (cm)	Boys	11 trials	611	-0.09 (-0.35, 0.16)	0.48	0.71	0.70
	Girls	11 trials	615	-0.16 (-0.41, 0.09)	0.21	0.07	
HC in childhood (cm)	Boys	3 trials	151	-0.18 (-0.71, 0.35)	0.50	0.59	0.80
	Girls	3 trials	166	-0.27 (-0.77, 0.23)	0.28	0.06	
HC z-scores in infancy	Boys	18 trials	1045	0.01 (-0.13, 0.16)	0.87	0.03	0.78
	Girls	18 trials	989	-0.02 (-0.17, 0.13)	0.82	0.11	
HC z-scores in toddlers	Boys	11 trials	611	-0.05 (-0.23, 0.13)	0.56	0.58	0.62
	Girls	11 trials	615	-0.12 (-0.30, 0.06)	0.20	0.12	
**Body composition**							
Fat mass in infancy (kg)	Boys	8 trials	291	0.01 (-0.12, 0.14)	0.83	0.006	0.89
	Girls	8 trials	278	0.00 (-0.13, 0.13)	0.99	0.24	
Fat mass in childhood (kg)	Boys	2 trials	106	0.09 (-0.84, 1.02)	0.84	0.003	0.93
	Girls	2 trials	138	0.15 (-0.67, 0.98)	0.72	0.32	
Fat mass in adolescence (kg)	Boys	2 trials	49	0.78 (-2.69, 4.24)	0.66	0.08	0.72
	Girls	2 trials	49	-0.13 (-3.64, 3.37)	0.94	0.21	
Fat mass at >3 years (kg)	Boys	4 trials	155	0.40 (-0.80, 1.59)	0.52	0.40	0.67
	Girls	4 trials	187	0.05 (-1.06, 1.14)	0.94	0.004	
Fat mass index in infancy (kg/m^2)	Boys	8 trials	288	0.01 (-0.28, 0.29)	0.96	0.02	0.78
	Girls	8 trials	278	-0.05 (-0.34, 0.24)	0.73	0.55	
Fat mass index in childhood (kg/m^2)	Boys	2 trials	106	0.12 (-0.52, 0.76)	0.71	0.003	0.91
	Girls	2 trials	138	0.07 (-0.49, 0.64)	0.80	0.33	
Fat mass index in adolescence (kg/m^2)	Boys	2 trials	49	0.29 (-1.20, 1.79)	0.70	0.08	0.94
	Girls	2 trials	49	0.21 (-1.29, 1.71)	0.78	0.04	
Fat mass index at >3 years (kg/m^2)	Boys	4 trials	155	0.22 (-0.41, 0.84)	0.50	0.26	0.76
	Girls	4 trials	187	0.09 (-0.49, 0.66)	0.77	0.40	
	Boys	8 trials	289	-0.35 (-1.87, 1.17)	0.65	0.03	0.53

Outcome	Subgroup	No. of trials	No. of participants	aMD (95% CI)	P for overall effect	P for heterogeneity	P for subgroup interaction
Percent fat mass in infancy (%)	Girls	8 trials	278	-0.71 (-2.26, 0.84)	0.37	0.87	
Percent fat mass in childhood (%)	Boys	2 trials	106	1.11 (-2.68, 4.90)	0.57	0.006	0.73
	Girls	2 trials	138	0.21 (-3.15, 3.56)	0.91	0.35	
Percent fat mass in adolescence (%)	Boys	2 trials	49	-0.89 (-5.60, 3.81)	0.71	0.20	0.81
	Girls	2 trials	49	-0.11 (-4.18, 4.61)	0.97	0.07	
Percent fat mass at >3 years (%)	Boys	4 trials	155	0.58 (_2.40, 3.56)	0.70	0.02	0.83
	Girls	4 trials	187	0.14 (-2.60, 2.87)	0.92	0.46	
Lean mass in infancy (kg)	Boys	8 trials	291	0.16 (-0.05, 0.37)	0.13	0.004	0.82
	Girls	8 trials	278	0.13 (-0.08, 0.34)	0.23	0.28	
Lean mass in childhood (kg)	Boys	2 trials	106	-0.70 (-1.71, 0.32)	0.18	0.37	0.31
	Girls	2 trials	138	-0.01 (-0.90, 0.89)	0.99	0.80	
Lean mass in adolescence (kg)	Boys	2 trials	49	0.15 (-2.79, 3.08)	0.92	0.22	0.71
	Girls	2 trials	49	-0.65 (-3.63, 2.32)	0.66	0.75	
Lean mass at >3 years (kg)	Boys	4 trials	155	-0.52 (-1.63, 0.59)	0.36	0.33	0.67
	Girls	4 trials	187	-0.19 (-1.20, 0.83)	0.72	0.93	
Lean mass index in infancy (kg/m²)	Boys	8 trials	288	0.21 (-0.13, 0.55)	0.23	0.20	0.96
	Girls	8 trials	278	0.22 (-0.13, 0.56)	0.22	0.79	
Lean mass index in childhood (kg/m²)	Boys	2 trials	106	-0.45 (-1.02, 0.12)	0.12	0.79	0.20
	Girls	2 trials	138	0.05 (-0.46, 0.55)	0.86	0.48	
Lean mass index in adolescence (kg/m²)	Boys	2 trials	49	-0.16 (-1.20, 0.88)	0.77	0.08	0.93
	Girls	2 trials	49	-0.09 (-1.14, 0.97)	0.87	0.99	
Lean mass index at >3 years (kg/m²)	Boys	4 trials	155	-0.38 (-0.88, 0.12)	0.14	0.19	0.29
	Girls	4 trials	187	-0.02 (-0.47, 0.44)	0.95	0.89	
**Bone development**							
BMC in infancy (g)	Boys	6 trials	158	7.86 (-5.72, 21.45)	0.26	0.06	0.63
	Girls	6 trials	155	12.58 (-1.28, 26.43)	0.08	0.79	

Outcome	Subgroup	No. of trials	No. of participants	aMD (95% CI)	P for overall effect	P for heterogeneity	P for subgroup interaction
BMC in adolescence (g)	Boys	2 trials	49	-0.31 (-146.51, 145.89)	0.99	0.72	0.61
	Girls	2 trials	49	-54.74 (-203.05, 93.58)	0.47	0.81	
BMD in infancy	Boys	5 trials	131	0.005 (-0.008, 0.018)	0.45	0.20	0.55
	Girls	5 trials	136	0.011 (-0.002, 0.023)	0.11	0.98	
BMD in adolescence	Boys	2 trials	49	0.002 (-0.039, 0.043)	0.92	0.51	0.44
	Girls	2 trials	49	-0.021 (-0.062, 0.021)	0.32	0.82	

Abbreviation: BMI: body mass index; HC: head circumference; BMC: bone mineral content; BMD: bone mineral density; aMD: adjusted mean difference.
Mean differences were adjusted for gestational age and birthweight z-scores.

Table S3. Subgroup analyses of size for gestation of the infants

Outcome	Subgroup	No. of trials	No. of participants	aMD (95% CI)	P for overall effect	P for heterogeneity	P for subgroup interaction
**BMI**							
BMI in childhood (kg/m^2)	AGA	3 trials	280	0.02 (-0.38, 0.41)	0.93	0.63	0.21
	SGA	3 trials	53	-0.63 (-1.55, 0.29)	0.18	0.17	
BMI in infancy (kg/m^2)	AGA	18 trials	1442	0.06 (-0.09, 0.21)	0.44	0.39	0.52
	SGA	18 trials	566	-0.03 (-0.28, 0.21)	0.79	0.32	
BMI in toddlers (kg/m^2)	AGA	10 trials	865	-0.06 (-0.23, 0.11)	0.48	0.93	0.77
	SGA	10 trials	329	-0.11 (-0.39, 0.17)	0.44	0.75	
BMI in adolescence (kg/m^2)	AGA	2 trials	83	0.04 (-1.54, 1.61)	0.96	0.03	0.68
	SGA	2 trials	20	0.82 (-2.54, 4.18)	0.63	0.01	
BMI at >3 years (kg/m^2)	AGA	5 trials	363	0.01 (-0.46, 0.48)	0.97	0.01	0.79
	SGA	5 trials	73	-0.15 (-1.20, 0.90)	0.78	0.01	
BMI z-scores in infancy	AGA	18 trials	1442	-0.04 (-0.22, 0.13)	0.63	0.45	0.36
	SGA	18 trials	566	0.05 (-0.06, 0.16)	0.37	0.14	
BMI z-scores in toddlers	AGA	10 trials	865	-0.07 (-0.20, 0.06)	0.32	0.81	0.77
	SGA	10 trials	329	-0.10 (-0.31, 0.11)	0.34	0.98	
BMI z-scores in childhood	AGA	3 trials	280	-0.03 (-0.28, 0.23)	0.85	0.72	0.29
	SGA	3 trials	53	-0.37 (-0.96, 0.22)	0.21	0.25	
BMI z-scores in adolescence	AGA	2 trials	83	-0.05 (-0.63, 0.53)	0.87	0.02	1.00
	SGA	2 trials	20	-0.05 (-1.29, 1.18)	0.93	0.004	
BMI z-scores at >3 years	AGA	5 trials	363	-0.04 (-0.28, 0.19)	0.73	0.06	0.55
	SGA	5 trials	73	-0.22 (-0.74, 0.31)	0.42	0.01	
**Weight**							
Weight in infancy (kg)	AGA	18 trials	1462	0.11 (-0.02, 0.24)	0.08	0.03	0.62
	SGA	18 trials	570	0.05 (-0.16, 0.26)	0.62	0.05	
Weight in toddlers (kg)	AGA	10 trials	872	0.02 (-0.16, 0.20)	0.80	0.92	0.68
	SGA	10 trials	332	-0.05 (-0.34, 0.25)	0.74	0.87	
Weight in childhood (kg)	AGA	3 trials	280	-0.18 (-1.01, 0.66)	0.68	0.58	0.14
	SGA	3 trials	53	-1.79 (-3.73, 0.16)	0.07	0.13	
Weight in adolescence (kg)	AGA	2 trials	83	-0.53 (-4.99, 3.94)	0.82	0.14	0.54
	SGA	2 trials	20	2.74 (-6.79, 12.26)	0.57	0.01	
Weight at >3 years (kg)	AGA	5 trials	363	-0.26 (-1.45, 0.93)	0.67	0.09	0.85
	SGA	5 trials	73	-0.55 (-3.23, 2.13)	0.69	0.001	
Weight z-scores in infancy	AGA	18 trials	1462	0.11 (-0.01, 0.22)	0.07	0.02	0.30
	SGA	18 trials	570	-0.01 (-0.19, 0.18)	0.95	0.16	
	AGA	10 trials	872	0.05 (-0.08, 0.18)	0.43	0.75	0.40

Outcome	Subgroup	No. of trials	No. of participants	aMD (95% CI)	P for overall effect	P for heterogeneity	P for subgroup interaction
Weight z-scores in toddlers	SGA	10 trials	332	-0.05 (0.26, 0.16)	0.63	0.74	
Weight z-scores in childhood	AGA	3 trials	278	-0.09 (-0.33, 0.15)	0.47	0.69	0.06
	SGA	3 trials	53	-0.67 (-1.23, -0.12)	0.02	0.49	
**Length/ height**							
Length in infancy (cm)	AGA	18 trials	1442	0.38 (-0.05, 0.80)	0.08	0.006	0.99
	SGA	18 trials	566	0.38 (-0.29, 1.05)	0.27	0.09	
Height in toddlers (cm)	AGA	10 trials	868	0.22 (-0.29, 0.72)	0.40	0.46	0.50
	SGA	10 trials	330	0.12 (-0.71, 0.64)	0.78	0.39	
Height in childhood (cm)	AGA	3 trials	280	-0.64 (-1.91, 0.62)	0.32	0.77	0.28
	SGA	3 trials	54	-2.41 (-5.35, 0.53)	0.11	0.76	
Height in adolescence (cm)	AGA	2 trials	83	-1.46 (-5.40, 2.49)	0.47	0.90	0.61
	SGA	2 trials	20	0.97 (-7.44, 9.38)	0.82	0.10	
Height at >3 years (cm)	AGA	5 trials	363	-0.80 (-2.12, 0.51)	0.23	0.96	0.48
	SGA	5 trials	73	-1.97 (-4.92, 0.97)	0.19	0.13	
Length z-scores in infancy	AGA	18 trials	1442	0.06 (-0.01, 0.24)	0.07	0.05	0.57
	SGA	18 trials	566	0.05 (-0.15, 0.25)	0.64	0.40	
Height z-scores in toddlers	AGA	10 trials	868	0.15 (0.01, 0.30)	0.04	0.13	0.36
	SGA	10 trials	330	0.02 (-0.22, 0.25)	0.87	0.54	
Height z-scores in childhood	AGA	3 trials	280	-0.07 (-0.30, 0.15)	0.52	0.40	0.02
	SGA	3 trials	54	-0.77 (-1.28, -0.25)	0.004	0.56	
Height z-scores in adolescence	AGA	2 trials	83	-0.06 (-0.52, 0.39)	0.79	0.50	0.73
	SGA	2 trials	20	0.13 (-0.84, 1.09)	0.80	0.002	
Height z-scores at >3 years	AGA	5 trials	363	-0.07 (-0.27, 0.13)	0.47	0.72	0.08
	SGA	5 trials	73	-0.51 (-0.95, -0.06)	0.03	0.002	
Weight for length z-scores in infancy	AGA	18 trials	1442	0.06 (-0.05, 0.18)	0.29	0.15	0.23
	SGA	18 trials	566	-0.07 (-0.24, 0.11)	0.45	0.43	
Weight for length z-scores in toddlers	AGA	10 trials	865	-0.03 (-0.16, 0.10)	0.67	0.77	0.58
	SGA	10 trials	329	-0.10 (-0.31, 0.11)	0.36	0.96	
**HC**							
HC in infancy (cm)	AGA	18 trials	1465	0.06 (-0.14, 0.27)	0.54	0.05	0.34
	SGA	18 trials	569	-0.13 (-0.46, 0.21)	0.46	0.12	
HC in toddlers (cm)	AGA	11 trials	896	-0.11 (-0.32, 0.10)	0.30	0.62	0.78
	SGA	11 trials	330	-0.17 (-0.52, 0.18)	0.34	0.31	
HC in childhood (cm)	AGA	3 trials	264	-0.15 (-0.55, 0.25)	0.47	0.61	0.54
	SGA	3 trials	53	-0.45 (-1.36, 0.45)	0.33	0.84	

Outcome	Subgroup	No. of trials	No. of participants	aMD (95% CI)	P for overall effect	P for heterogeneity	P for subgroup interaction
HC z-scores in infancy	AGA	18 trials	1465	0.06 (-0.06, 0.18)	0.34	0.02	0.08
	SGA	18 trials	569	-0.15 (-0.35, 0.05)	0.14	0.67	
HC z-scores in toddlers	AGA	11 trials	896	0.08 (-0.21, 0.09)	0.41	0.70	0.56
	SGA	11 trials	330	-0.15 (-0.40, 0.10)	0.24	0.29	
**Body composition**							
Fat mass in infancy (kg)	AGA	8 trials	422	0.01 (-0.10, 0.11)	0.93	0.03	0.70
	SGA	8 trials	147	0.05 (-0.14, 0.23)	0.61	0.11	
Fat mass in childhood (kg)	AGA	2 trials	211	0.21 (-0.46, 0.88)	0.54	0.37	0.59
	SGA	2 trials	33	-0.29 (-1.98, 1.40)	0.73	0.90	
Fat mass in adolescence (kg)	AGA	2 trials	78	-0.03 (-2.77, 2.72)	0.99	0.08	0.50
	SGA	2 trials	20	2.15 (-3.53, 7.84)	0.45	0.02	
Fat mass at >3 years (kg)	AGA	4 trials	289	0.13 (-0.76, 1.01)	0.78	0.02	0.63
	SGA	4 trials	53	0.68 (-1.41, 2.77)	0.52	0.04	
Fat mass index in infancy (kg/m^2)	AGA	8 trials	419	-0.03 (-0.27, 0.21)	0.83	0.03	0.78
	SGA	8 trials	147	0.04 (-0.36, 0.44)	0.85	0.07	
Fat mass index in childhood (kg/m^2)	AGA	2 trials	211	0.14 (-0.32, 0.60)	0.56	0.38	0.70
	SGA	2 trials	33	-0.11 (-1.27, 1.06)	0.86	0.91	
Fat mass index in adolescence (kg/m^2)	AGA	2 trials	78	0.17 (-1.02, 1.36)	0.78	0.02	0.69
	SGA	2 trials	20	0.72 (-1.73, 3.17)	0.56	0.06	
Fat mass index at >3 years (kg/m^2)	AGA	4 trials	289	0.14 (-0.32, 0.60)	0.55	0.02	0.17
	SGA	4 trials	53	0.19 (-0.90, 1.28)	0.73	0.18	
Percent fat mass in infancy (%)	AGA	8 trials	420	-0.79 (-2.05, 0.47)	0.22	0.04	0.35
	SGA	8 trials	147	0.38 (-1.75, 2.51)	0.73	0.09	
Percent fat mass in childhood (%)	AGA	2 trials	211	0.81 (-1.91, 3.53)	0.56	0.42	0.71
	SGA	2 trials	33	-0.59 (-7.44, 6.26)	0.87	0.93	
Percent fat mass in adolescence (%)	AGA	2 trials	78	-0.68 (-4.45, 3.09)	0.72	0.03	0.69
	SGA	2 trials	20	1.04 (-6.71, 8.80)	0.79	0.23	
Percent fat mass at >3 years (%)	AGA	4 trials	289	0.43 (-1.76, 2.63)	0.70	0.26	0.86
	SGA	4 trials	53	-0.07 (-5.26, 5.13)	0.98	0.56	
Lean mass in infancy (kg)	AGA	8 trials	422	0.20 (0.03, 0.37)	0.03	0.14	0.35
	SGA	8 trials	147	0.04 (-0.25, 0.32)	0.82	0.12	
Lean mass in childhood (kg)	AGA	2 trials	211	-0.24 (-0.98, 0.50)	0.53	0.35	0.73
	SGA	2 trials	33	-0.59 (-2.46, 1.28)	0.54	0.87	
	AGA	2 trials	78	-0.47 (-2.79, 1.84)	0.69	0.48	0.70

Outcome	Subgroup	No. of trials	No. of participants	aMD (95% CI)	P for overall effect	P for heterogeneity	P for subgroup interaction
Lean mass in adolescence (kg)	SGA	2 trials	20	0.56 (-4.23, 5.35)	0.82	0.13	
Lean mass at >3 years (kg)	AGA	4 trials	289	-0.33 (-1.15, 0.49)	0.43	0.57	0.95
	SGA	4 trials	53	-0.27 (-2.20, 1.67)	0.79	0.23	
Lean mass index in infancy (kg/m²)	AGA	8 trials	419	0.33 (0.05. 0.61)	0.02	0.05	0.10
	SGA	8 trials	147	-0.14 (-0.61, 0.34)	0.57	0.84	
Lean mass index in childhood (kg/m²)	AGA	2 trials	211	-0.17 (-0.58, 0.25)	0.44	0.46	0.85
	SGA	2 trials	33	-0.05 (-1.10, 0.99)	0.92	0.34	
Lean mass index in adolescence (kg/m²)	AGA	2 trials	78	-0.19 (-1.01, 0.63)	0.64	0.05	0.76
	SGA	2 trials	20	0.10 (-1.59, 1.80)	0.91	0.11	
Lean mass index at >3 years (kg/m²)	AGA	4 trials	289	-0.20 (-0.57, 0.17)	0.29	0.08	0.64
	SGA	4 trials	53	0.03 (-0.85, 0.90)	0.95	0.35	
**Bone development**							
BMC in infancy (g)	AGA	6 trials	248	11.06 (0.20, 21.93)	0.046	0.31	0.90
	SGA	6 trials	65	9.50 (-11.85, 30.54)	0.38	0.21	
BMC in adolescence (g)	AGA	2 trials	78	-32.23 (-147.59, 83.14)	0.58	0.91	0.88
	SGA	2 trials	20	-12.33 (-251.26, 226.59)	0.92	0.17	
BMD in infancy	AGA	5 trials	212	0.007 (-0.003, 0.018)	0.15	0.45	0.98
	SGA	5 trials	55	0.007 (-0.013, 0.027)	0.49	0.59	
BMD in adolescence	AGA	2 trials	78	-0.006 (-0.038, 0.027)	0.72	0.51	0.57
	SGA	2 trials	20	-0.027 (-0.094, 0.040)	0.42	0.56	

Abbreviation: AGA: appropriate for gestational age; SGA: small for gestational age; BMI: body mass index; HC: head circumference; BMC: bone mineral content; BMD: bone mineral density; aMD: adjusted mean difference.
Mean differences were adjusted for sex and gestational age.

Table S4. Subgroup analyses of size of infant at birth

Outcome	Subgroup	No. of trials	No. of participants	aMD (95% CI)	P for overall effect	P for heterogeneity	P for subgroup interaction
**BMI**							
BMI in childhood (kg/m²)	≤1 kg	3 trials	76	0.47 (-0.37, 1.31)	0.27	0.13	0.12
	>1 kg	3 trials	257	-0.25 (-0.66, 0.15)	0.22	0.26	
BMI in infancy (kg/m²)	≤1 kg	17 trials	436	0.04 (-0.24, 0.32)	0.77	0.89	0.89
	>1 kg	18 trials	1572	0.02 (-0.13, 0.17)	0.78	0.36	
BMI in toddlers (kg/m²)	≤1 kg	9 trials	231	-0.07 (-0.40, 0.27)	0.70	0.47	0.91
	>1 kg	10 trials	963	-0.09 (-0.25, 0.08)	0.30	0.44	
BMI in adolescence (kg/m²)	≤1 kg	2 trials	10	-3.05 (-6.59, 0.50)	0.08	0.20	0.22
	>1 kg	2 trials	93	0.57 (-0.95, 2.08)	0.46	0.04	
BMI at >3 years (kg/m²)	≤1 kg	5 trials	86	0.13 (-0.70, 0.96)	0.75	0.08	0.76
	>1 kg	5 trials	350	-0.05 (-0.54, 0.45)	0.86	0.01	
BMI z-scores in infancy	≤1 kg	17 trials	436	0.008 (-0.19, 0.21)	0.93	0.91	0.92
	>1 kg	18 trials	1572	0.02 (-0.86, 0.13)	0.71	0.35	
BMI z-scores in toddlers	≤1 kg	9 trials	231	-0.08 (-0.34, 0.17)	0.52	0.41	1.00
	>1 kg	10 trials	963	-0.08 (-0.21, 0.04)	0.19	0.38	
BMI z-scores in childhood	≤1 kg	3 trials	76	0.33 (-0.20, 0.86)	0.22	0.11	0.07
	>1 kg	3 trials	257	-0.21 (-0.46, 0.05)	0.12	0.45	
BMI z-scores in adolescence	≤1 kg	2 trials	10	-1.19 (-3.01, 0.63)	0.16	0.38	0.13
	>1 kg	2 trials	93	0.13 (-0.41, 0.68)	0.63	0.03	
BMI z-scores at >3 years	≤1 kg	5 trials	86	0.16 (-0.35, 0.66)	0.54	0.11	0.36
	>1 kg	5 trials	350	-0.12 (-0.36, 0.12)	0.33	0.04	
**Weight**							
Weight in infancy (kg)	≤1 kg	17 trials	449	0.06 (-0.18, 0.29)	0.63	0.43	0.83
	>1 kg	18 trials	1583	0.09 (-0.04, 0.21)	0.17	0.12	
Weight in toddlers (kg)	≤1 kg	9 trials	233	-0.18 (-0.54, 0.18)	0.33	0.87	0.30
	>1 kg	10 trials	971	0.03 (-0.14, 0.21)	0.73	0.81	

Outcome	Subgroup	No. of trials	No. of participants	aMD (95% CI)	P for overall effect	P for heterogeneity	P for subgroup interaction
Weight in childhood (kg)	≤1 kg	3 trials	76	-0.26 (-1.86, 1.33)	0.75	0.38	0.75
	>1 kg	3 trials	257	-0.56 (-1.42, 0.31)	0.21	0.55	
Weight in adolescence (kg)	≤1 kg	2 trials	10	-5.12 (-18.06, 7.82)	0.43	N/A	0.40
	>1 kg	2 trials	93	0.77 (-3.47, 5.00)	0.72	0.16	
Weight at >3 years (kg)	≤1 kg	5 trials	86	-0.82 (-3.27, 1.64)	0.52	0.55	0.68
	>1 kg	5 trials	350	-0.24 (-1.45, 0.98)	0.70	0.13	
Weight z-scores in infancy	≤1 kg	17 trials	449	0.02 (-0.19, 0.23)	0.85	0.58	0.65
	>1 kg	18 trials	1583	0.08 (-0.04, 0.19)	0.18	0.18	
Weight z-scores in toddlers	≤1 kg	9 trials	233	0.13 (-0.36, 0.16)	0.44	0.86	0.33
	>1 kg	10 trials	971	0.04 (-0.09, 0.17)	0.53	0.90	
Weight z-scores in childhood	≤1 kg	3 trials	75	-0.04 (-0.51, 0.42)	0.85	0.44	0.46
	>1 kg	3 trials	256	-0.24 (-0.49, 0.01)	0.06	0.87	
**Length/ height**							
Length in infancy (cm)	≤1 kg	17 trials	436	0.29 (-0.48, 1.06)	0.46	0.63	0.89
	>1 kg	18 trials	1572	0.35 (-0.05, 0.76)	0.09	0.10	
Height in toddlers (cm)	≤1 kg	9 trials	231	-0.49 (-1.48, 0.51)	0.34	0.95	0.15
	>1 kg	10 trials	967	0.33 (-0.16, 0.81)	0.19	0.11	
Height in childhood (cm)	≤1 kg	3 trials	77	-2.54 (-4.97, -0.11)	0.04	0.85	0.16
	>1 kg	3 trials	257	-0.56 (-1.89, 0.77)	0.41	0.84	
Height in adolescence (cm)	≤1 kg	2 trials	10	-1.90 (-13.39, 9.59)	0.74	N/A	0.87
	>1 kg	2 trials	93	-0.87 (-4.63, 2.89)	0.65	0.96	
Height at >3 years (cm)	≤1 kg	5 trials	87	-2.49 (-5.20, 0.23)	0.07	0.48	0.24
	>1 kg	5 trials	350	-0.66 (-2.01, 0.68)	0.33	0.98	
Length z-scores in infancy	≤1 kg	17 trials	436	0.02 (-0.21, 0.25)	0.85	0.66	0.55
	>1 kg	18 trials	1572	0.10 (-0.02, 0.22)	0.10	0.08	
Height z-scores in toddlers	≤1 kg	9 trials	231	-0.09 (-0.37, 0.20)	0.55	0.97	0.13
	>1 kg	10 trials	967	0.16 (0.02, 0.29)	0.03	0.18	
	≤1 kg	3 trials	77	-0.48 (-0.91, -0.05)	0.03	0.48	0.15

Outcome	Subgroup	No. of trials	No. of participants	aMD (95% CI)	P for overall effect	P for heterogeneity	P for subgroup interaction
Height z-scores in childhood	>1 kg	3 trials	257	-0.12 (-0.35, 0.12)	0.33	0.40	
Height z-scores in adolescence	≤1 kg	2 trials	10	-0.27 (-1.58, 1.05)	0.69	N/A	0.67
	>1 kg	2 trials	93	0.22 (-0.41, 0.46)	0.90	0.44	
Height z-scores at >3 years	≤1 kg	5 trials	87	-0.45 (-0.86, -0.04)	0.03	0.43	0.12
	>1 kg	5 trials	350	-0.08 (-0.28, 0.13)	0.45	0.56	
Weight for length z-scores in infancy	≤1 kg	17 trials	436	-0.01 (-0.2\, 0.19)	0.93	0.91	0.79
	>1 kg	18 trials	1572	0.02 (-0.08, 0.13)	0.69	0.19	
Weight for length z-scores in toddlers	≤1 kg	9 trials	231	-0.09 (-0.35, 0.16)	0.49	0.49	0.75
	>1 kg	10 trials	963	-0.05 (-0.17, 0.08)	0.47	0.57	
**HC**							
HC in infancy (cm)	≤1 kg	17 trials	449	-0.02 (-0.40, 0.35)	0.90	0.71	0.91
	>1 kg	18 trials	1585	0.001 (-0.20, 0.20)	0.99	0.52	
HC in toddlers (cm)	≤1 kg	18 trials	261	-0.06 (-0.46, 0.33)	0.76	0.62	0.68
	>1 kg	19 trials	965	-0.16 (-0.36, 0.05)	0.14	0.55	
HC in childhood (cm)	≤1 kg	3 trials	74	-0.51 (-1.26, 0.24)	0.18	0.84	0.40
	>1 kg	3 trials	243	-0.14 (-0.55, 0.27)	0.51	0.17	
HC z-scores in infancy	≤1 kg	17 trials	449	-0.02 (-0.24, 0.20)	0.85	0.69	0.90
	>1 kg	18 trials	1585	-0.005 (-0.12, 0.11)	0.94	0.41	
HC z-scores in toddlers	≤1 kg	18 trials	261	-0.05 (-0.33, 0.23)	0.72	0.64	0.74
	>1 kg	19 trials	965	-0.11 (-0.25, 0.04)	0.16	0.70	
**Body composition**							
Fat mass in infancy (kg)	≤1 kg	8 trials	161	-0.03 (-0.21, 0.14)	0.71	0.65	0.57
	>1 kg	8 trials	408	0.03 (-0.08, 0.14)	0.62	0.06	
Fat mass in childhood (kg)	≤1 kg	2 trials	44	0.95 (-0.52, 2.43)	0.20	0.07	0.23
	>1 kg	2 trials	200	-0.05 (-0.74, 0.64)	0.88	0.96	
	≤1 kg	2 trials	10	0.68 (-1.94, 3.30)	0.61	N/A	0.73

Outcome	Subgroup	No. of trials	No. of participants	aMD (95% CI)	P for overall effect	P for heterogeneity	P for subgroup interaction
Fat mass in adolescence (kg)	>1 kg	2 trials	88	-0.79 (-8.56, 6.98)	0.84	0.11	
Fat mass at >3 years (kg)	≤1 kg	4 trials	54	0.59 (-1.48, 2.66)	0.58	0.30	0.70
	>1 kg	4 trials	288	0.14 (-0.75, 1.03)	0.75	0.11	
Fat mass index in infancy (kg/m²)	≤1 kg	8 trials	160	-0.20 (-0.58, 0.19)	0.32	0.90	0.28
	>1 kg	8 trials	406	0.06 (-0.19, 0.30)	0.65	0.03	
Fat mass index in childhood (kg/m²)	≤1 kg	2 trials	44	0.74 (-0.28, 1.75)	0.15	0.03	0.17
	>1 kg	2 trials	200	-0.04 (-0.52, 0.43)	0.86	0.98	
Fat mass index in adolescence (kg/m²)	≤1 kg	2 trials	10	-0.79 (-4.11, 2.52)	0.64	N/A	0.47
	>1 kg	2 trials	88	0.48 (-0.65, 1.60)	0.40	0.04	
Fat mass index at >3 years (kg/m²)	≤1 kg	4 trials	54	0.46 (-0.62, 1.54)	0.40	0.11	0.54
	>1 kg	4 trials	288	0.09 (-0.37, 0.56)	0.69	0.06	
Percent fat mass in infancy (%)	≤1 kg	8 trials	161	-1.27 (-3.31, 0.76)	0.22	0.96	0.38
	>1 kg	8 trials	406	-0.20 (-1.48, 1.08)	0.76	0.04	
Percent fat mass in childhood (%)	≤1 kg	2 trials	44	4.16 (-1.82, 10.14)	0.17	0.03	0.20
	>1 kg	2 trials	200	-0.15 (-2.94, 2.64)	0.92	0.94	
Percent fat mass in adolescence (%)	≤1 kg	2 trials	10	0.32 (-10.20, 10.84)	0.95	N/A	0.92
	>1 kg	2 trials	88	-0.23 (-3.80, 3.33)	0.90	0.07	
Percent fat mass at >3 years (%)	≤1 kg	4 trials	54	3.49 (-1.65, 8.62)	0.18	0.08	0.20
	>1 kg	4 trials	288	-0.19 (-2.41, 2.02)	0.86	0.55	
Lean mass in infancy (kg)	≤1 kg	8 trials	161	0.11 (-0.17, 0.39)	0.43	0.66	0.76
	>1 kg	8 trials	408	0.16 (-0.01, 0.33)	0.07	0.10	
Lean mass in childhood (kg)	≤1 kg	2 trials	44	0.02 (-1.62, 1.65)	0.99	0.93	0.64
	>1 kg	2 trials	200	-0.41 (-1.17, 0.35)	0.29	0.38	
Lean mass in adolescence (kg)	≤1 kg	2 trials	10	-4.02 (-10.49, 2.46)	0.22	N/A	0.23
	>1 kg	2 trials	88	0.22 (-1.97, 2.40)	0.84	0.59	
Lean mass at >3 years (kg)	≤1 kg	4 trials	54	-0.77 (-2.69, 1.14)	0.43	0.37	0.63
	>1 kg	4 trials	288	-0.27 (-1.09, 0.56)	0.53	0.65	
Lean mass index in infancy (kg/m²)	≤1 kg	8 trials	160	0.05 (-0.40, 0.51)	0.82	0.52	0.43
	>1 kg	8 trials	406	0.27 (-0.02, 0.55)	0.07	0.37	
	≤1 kg	2 trials	44	0.37 (-0.54, 1.28)	0.42	0.15	0.91

Outcome	Subgroup	No. of trials	No. of participants	aMD (95% CI)	P for overall effect	P for heterogeneity	P for subgroup interaction
Lean mass index in childhood (kg/m²)	>1 kg	2 trials	200	-0.31 (-0.73, 0.11)	0.15	0.57	
Lean mass index in adolescence (kg/m²)	≤1 kg	2 trials	10	-1.47 (-3.76, 0.81)	0.20	N/A	0.22
	>1 kg	2 trials	88	0.03 (-0.74, 0.80)	0.94	0.03	
Lean mass index at >3 years (kg/m²)	≤1 kg	4 trials	54	0.03 (-0.84, 0.89)	0.95	0.02	0.58
	>1 kg	4 trials	288	-0.24 (-0.61, 0.14)	0.21	0.08	
**Bone development**							
BMC in infancy (g)	≤1 kg	6 trials	84	9.93 (-8.99, 28.86)	0.30	0.82	0.90
	>1 kg	6 trials	229	11.35 (-0.03, 22.72)	0.05	0.13	
BMC in adolescence (g)	≤1 kg	2 trials	10	-126.80 (-452.38, 198.78)	0.44	N/A	0.52
	>1 kg	2 trials	88	-14.77 (-124.48, 94.94)	0.79	0.74	
BMD in infancy	≤1 kg	5 trials	72	0.007 (-0.011, 0.024)	0.47	0.91	0.86
	>1 kg	5 trials	195	0.008 (-0.002, 0.019)	0.12	0.42	
BMD in adolescence	≤1 kg	2 trials	10	-0.055 (-0.146, 0.036)	0.23	N/A	0.29
	>1 kg	2 trials	88	-0.004 (-0.034, 0.027)	0.81	0.34	

Abbreviation: BMI: body mass index; HC: head circumference; BMC: bone mineral content; BMD: bone mineral density; aMD: adjusted mean difference; N/A: not applicable.

Mean differences were adjusted for sex and gestational age.

Table S5. Subgroup analyses of gestational age of infant at birth

Outcome	Subgroup	No. of trials	No. of participants	aMD (95% CI)	P for overall effect	P for heterogeneity	P for subgroup interaction
**BMI**							
BMI in childhood (kg/m²)	≤28 weeks	3 trials	121	0.62 (-0.03, 1.26)	0.06	0.15	0.01
	29 to 32 weeks	3 trials	187	-0.46 (-0.93, 0.01)	0.06	<.0001	
	33 to 36 weeks	2 trials	25	-0.84 (-1.91, 0.23)	0.51	0.13	
BMI in infancy (kg/m²)	≤28 weeks	17 trials	651	0.17 (-0.06, 0.40)	0.14	0.51	0.16
	29 to 32 weeks	15 trials	936	-0.08 (-0.26, 0.11)	0.42	0.02	
	33 to 36 weeks	7 trials	161	0.26 (-0.16, 0.68)	0.23	0.43	
BMI in toddlers (kg/m²)	≤28 weeks	9 trials	317	0.19 (-0.10, 0.48)	0.20	0.51	0.01
	29 to 32 weeks	9 trials	537	-0.29 (-0.51, -0.07)	0.01	0.03	
	33 to 36 weeks	6 trials	103	0.15 (-0.22, 0.52)	0.43	0.69	
BMI in adolescence (kg/m²)	≤28 weeks	2 trials	17	-2.07 (-5.44, 1.30)	0.21	0.62	0.25
	29 to 32 weeks	2 trials	70	0.27 (-1.52, 2.06)	0.76	0.006	
	33 to 36 weeks	2 trials	16	2.81 (-1.16, 6.78)	0.15	0.36	
BMI at >3 years (kg/m²)	≤28 weeks	5 trials	138	0.33 (-0.35, 1.01)	0.34	0.04	0.44
	29 to 32 weeks	5 trials	257	-0.26 (-0.85, 0.32)	0.30	<.0001	
	33 to 36 weeks	4 trials	41	0.12 (-1.46, 1.70)	0.78	0.04	
BMI z-scores in infancy	≤28 weeks	17 trials	651	0.11 (-0.06, 0.27)	0.20	0.61	0.50
	29 to 32 weeks	15 trials	936	-0.04 (-0.17, 0.10)	0.60	0.01	
	33 to 36 weeks	7 trials	161	0.15 (-0.18, 0.48)	0.38	0.48	
BMI z-scores in toddlers	≤28 weeks	9 trials	317	0.10 (-0.12, 0.32)	0.35	0.49	0.01
	29 to 32 weeks	9 trials	537	-0.23 (-0.40, -0.07)	0.006	0.04	
	33 to 36 weeks	6 trials	103	0.12 (-0.17, 0.42)	0.41	0.84	
BMI z-scores in childhood	≤28 weeks	3 trials	121	0.38 (-0.03, 0.79)	0.06	0.01	0.01
	29 to 32 weeks	3 trials	187	-0.33 (-0.62, -0.03)	0.03	<.0001	
	33 to 36 weeks	2 trials	25	-0.63 (-1.34, 0.09)	0.08	0.09	
BMI z-scores in adolescence	≤28 weeks	2 trials	17	0.83 (-1.00, 2.67)	0.34	0.34	0.56
	29 to 32 weeks	2 trials	70	-0.08 (-0.71, 0.55)	0.80	0.002	
	33 to 36 weeks	2 trials	16	-0.51 (-1.89, 0.87)	0.44	0.51	
BMI z-scores at >3 years	≤28 weeks	5 trials	138	0.27 (-0.11, 0.66)	0.27	0.13	0.10
	29 to 32 weeks	5 trials	257	-0.26 (-0.54, 0.01)	0.06	<.0001	
	33 to 36 weeks	4 trials	41	0.34 (-0.82, 0.55)	0.69	0.05	
**Weight**							
Weight in infancy (kg)	≤28 weeks	17 trials	672	0.11 (-0.09, 0.31)	0.29	0.06	0.86
	29 to 32 weeks	15 trials	939	0.03 (-0.13, 0.18)	0.76	0.06	
	33 to 36 weeks	7 trials	161	0.13 (-0.20, 0.45)	0.44	0.68	
	≤28 weeks	9 trials	323	-0.02 (-0.32, 0.29)	0.91	0.71	0.16

Outcome	Subgroup	No. of trials	No. of participants	aMD (95% CI)	P for overall effect	P for heterogeneity	P for subgroup interaction
Weight in toddlers (kg)	29 to 32 weeks	9 trials	540	-0.14 (-0.38, 0.10)	0.24	0.70	
	33 to 36 weeks	6 trials	104	0.38 (-0.043, 0.81)	0.08	0.78	
Weight in childhood (kg)	≤28 weeks	3 trials	121	0.45 (-0.93, 1.83)	0.52	0.21	0.22
	29 to 32 weeks	3 trials	187	-0.95 (-1.92, 0.01)	0.05	0.0001	
	33 to 36 weeks	1 trial	24	-1.60 (-4.18, 0.99)	0.21	N/A	
Weight in adolescence (kg)	≤28 weeks	2 trials	17	-5.34 (-15.00, 4.33)	0.25	0.75	0.24
	29 to 32 weeks	2 trials	70	0.15 (-0.489, 5.18)	0.95	0.06	
	33 to 36 weeks	2 trials	16	10.03 (-4.88, 24.94)	0.17	N/A	
Weight at >3 years (kg)	≤28 weeks	5 trials	138	-0.17 (-1.72, 1.39)	0.83	0.10	0.57
	29 to 32 weeks	5 trials	257	-0.66 (-2.16, 0.84)	0.39	0.001	
	33 to 36 weeks	3 trials	40	0.80 (-4.27, 5.87)	0.75	0.15	
Weight z-scores in infancy	≤28 weeks	17 trials	672	0.07 (-0.10, 0.23)	0.44	0.10	0.87
	29 to 32 weeks	15 trials	939	0.03 (-0.11, 0.17)	0.70	0.02	
	33 to 36 weeks	7 trials	161	0.13 (-0.17, 0.44)	0.39	0.80	
Weight z-scores in toddlers	≤28 weeks	9 trials	323	0.05 (-0.17, 0.27)	0.68	0.78	0.70
	29 to 32 weeks	9 trials	540	-0.10 (-0.26, 0.07)	0.25	0.07	
	33 to 36 weeks	6 trials	104	0.22 (-0.08, 0.51)	0.14	0.89	
Weight z-scores in childhood	≤28 weeks	3 trials	119	0.09 (-0.30, 0.48)	0.64	0.12	0.09
	29 to 32 weeks	3 trials	187	-0.29 (-0.57, -0.01)	0.04	0.003	
	33 to 36 weeks	1 trial	24	-1.01 (-1.81, -0.21)	0.02	N/A	
**Length/ height**							
Length in infancy (cm)	≤28 weeks	17 trials	651	0.20 (-0.48, 0.88)	0.57	0.02	0.94
	29 to 32 weeks	15 trials	936	0.26 (-0.26, 0.77)	0.33	0.62	
	33 to 36 weeks	7 trials	161	0.15 (-0.94, 1.23)	0.79	0.65	
Height in toddlers (cm)	≤28 weeks	9 trials	318	-0.55 (-1.46, 0.35)	0.23	0.66	0.25
	29 to 32 weeks	9 trials	538	0.18 (-0.49, 0.85)	0.60	0.34	
	33 to 36 weeks	6 trials	103	1.05 (-0.24, 2.34)	0.11	0.52	
Height in childhood (cm)	≤28 weeks	3 trials	121	-1.45 (-3.40, 0.51)	0.15	0.71	0.78
	29 to 32 weeks	3 trials	188	-0.92 (-2.55, 0.71)	0.27	0.33	
	33 to 36 weeks	1 trial	24	-1.00 (-4.93, 2.93)	0.61	N/A	
Height in adolescence (cm)	≤28 weeks	2 trials	17	-1.38 (-8.92, 6.17)	0.70	0.83	0.67
	29 to 32 weeks	2 trials	70	-1.12 (-5.73, 3.49)	0.63	0.77	
	33 to 36 weeks	2 trials	16	-0.95 (-10.76, 8.86)	0.84	N/A	
Height at >3 years (cm)	≤28 weeks	5 trials	138	-1.45 (-3.40, 0.51)	0.15	0.93	0.85
	29 to 32 weeks	5 trials	258	-0.92 (-2.55, 0.71)	0.27	0.81	
	33 to 36 weeks	3 trials	40	-1.00 (-4.93, 2.92)	0.61	0.73	
	≤28 weeks	17 trials	651	0.002 (-0.19, 0.19)	0.98	0.08	0.77
	29 to 32 weeks	15 trials	936	0.08 (-0.08, 0.23)	0.34	0.17	

Outcome	Subgroup	No. of trials	No. of participants	aMD (95% CI)	P for overall effect	P for heterogeneity	P for subgroup interaction
Length z-scores in infancy	33 to 36 weeks	7 trials	161	0.06 (-0.28, 0.40)	0.72	0.57	
Height z-scores in toddlers	≤28 weeks	9 trials	318	-0.06 (-0.31, 0.19)	0.64	0.76	0.38
	29 to 32 weeks	9 trials	538	0.11 (-0.07, 0.29)	0.25	0.11	
	33 to 36 weeks	6 trials	103	0.22 (-0.11, 0.54)	0.20	0.82	
Height z-scores in childhood	≤28 weeks	3 trials	121	-0.20 (-0.54, 0.14)	0.25	0.24	0.45
	29 to 32 weeks	3 trials	188	-0.15 (-0.42, 0.13)	0.29	0.70	
	33 to 36 weeks	1 trial	24	-0.83 (-1.57, -0.09)	0.03	N/A	
Height z-scores in adolescence	≤28 weeks	2 trials	17	-0.03 (-0.84, 0.78)	0.95	0.97	0.99
	29 to 32 weeks	2 trials	70	-0.02 (-0.55, 0.51)	0.94	0.43	
	33 to 36 weeks	2 trials	16	0.04 (-0.96, 1.02)	0.94	N/A	
Height z-scores at >3 years	≤28 weeks	5 trials	138	-0.18 (-0.49, 0.13)	0.26	0.53	0.53
	29 to 32 weeks	5 trials	258	-0.11 (-0.35, 0.14)	0.39	0.72	
	33 to 36 weeks	3 trials	40	-0.56 (-1.12, 0.01)	0.05	0.34	
Weight for length z-scores in infancy	≤28 weeks	17 trials	651	0.09 (-0.08, 0.26)	0.29	0.42	0.44
	29 to 32 weeks	15 trials	936	-0.03 (-0.16, 0.11)	0.70	0.007	
	33 to 36 weeks	7 trials	161	0.14 (-0.18, 0.45)	0.40	0.52	
Weight for length z-scores in toddlers	≤28 weeks	9 trials	317	0.09 (-0.13, 0.31)	0.41	0.54	0.01
	29 to 32 weeks	9 trials	537	-0.20 (-0.37, -0.04)	0.02	0.04	
	33 to 36 weeks	6 trials	103	0.27 (-0.06, 0.59)	0.11	0.77	
**HC**							
HC in infancy (cm)	≤28 weeks	17 trials	675	-0.10 (-0.42, 0.23)	0.56	0.60	0.78
	29 to 32 weeks	15 trials	939	0.002 (-0.26, 0.26)	0.99	0.05	
	33 to 36 weeks	7 trials	160	-0.10 (-0.66, 0.46)	0.73	0.31	
HC in toddlers (cm)	≤28 weeks	10 trials	370	-0.31 (-0.68, 0.05)	0.09	0.65	0.74
	29 to 32 weeks	9 trials	515	-0.20 (-0.47, 0.07)	0.15	0.85	
	33 to 36 weeks	5 trials	102	-0.03 (-0.57, 0.52)	0.92	0.78	
HC in childhood (cm)	≤28 weeks	3 trials	111	-0.14 (-0.85, 0.56)	0.69	0.46	0.59
	29 to 32 weeks	3 trials	181	-0.35 (-0.78, 0.09)	0.12	0.80	
	33 to 36 weeks	1 trial	24	0.03(-0.99, 1.05)	0.95	N/A	
HC z-scores in infancy	≤28 weeks	17 trials	675	-0.05 (-0.23, 0.13)	0.60	0.48	0.79
	29 to 32 weeks	15 trials	939	0.006 (-0.15, 0.17)	0.94	0.03	
	33 to 36 weeks	7 trials	160	-0.09 (-0.44, 0.27)	0.63	0.12	
HC z-scores in toddlers	≤28 weeks	10 trials	370	-0.22 (-0.48, 0.04)	0.09	0.81	0.79
	29 to 32 weeks	9 trials	515	-0.13 (-0.33, 0.06)	0.17	0.74	
	33 to 36 weeks	5 trials	102	-0.07 (-0.44, 0.30)	0.71	0.89	
**Body composition**							

Outcome	Subgroup	No. of trials	No. of participants	aMD (95% CI)	P for overall effect	P for heterogeneity	P for subgroup interaction
Fat mass in infancy (kg)	≤28 weeks	8 trials	247	-0.03 (-0.18, 0.12)	0.69	0.62	0.63
	29 to 32 weeks	7 trials	303	0.01 (-0.12, 0.14)	0.87	0.01	
	33 to 36 weeks	2 trials	19	0.26 (-0.33, 0.85)	0.36	0.21	
Fat mass in childhood (kg)	≤28 weeks	2 trials	76	0.79 (-0.16, 1.75)	0.10	0.03	0.24
	29 to 32 weeks	2 trials	146	-0.34 (-1.20, 0.52)	0.43	0.89	
	33 to 36 weeks	1 trial	22	0.47(-1.96, 2.89)	0.69	N/A	
Fat mass in adolescence (kg)	≤28 weeks	2 trials	16	0.21 (-1.05, 1.47)	0.74	0.73	0.32
	29 to 32 weeks	2 trials	66	-0.06 (-1.17, 1.05)	0.92	0.03	
	33 to 36 weeks	2 trials	16	1.61 (-1.38, 4.59)	0.28	N/A	
Fat mass at >3 years (kg)	≤28 weeks	4 trials	92	0.21 (-1.05, 1.47)	0.74	0.06	0.43
	29 to 32 weeks	4 trials	212	-0.06 (-1.17, 1.05)	0.92	0.004	
	33 to 36 weeks	3 trials	38	1.61 (-1.38, 4.59)	0.28	0.23	
Fat mass index in infancy (kg/m^2)	≤28 weeks	8 trials	246	-0.18 (-0.50, 0.14)	0.27	0.71	0.36
	29 to 32 weeks	7 trials	301	0.06 (-0.22, 0.33)	0.69	0.004	
	33 to 36 weeks	2 trials	19	0.63 (-1.09, 2.35)	0.44	0.19	
Fat mass index in childhood (kg/m^2)	≤28 weeks	2 trials	76	0.55 (-0.05, 1.15)	0.07	0.02	0.21
	29 to 32 weeks	2 trials	146	-0.25 (-0.84, 0.34)	0.41	0.84	
	33 to 36 weeks	1 trial	22	0.49 (-1.49, 2.46)	0.61	N/A	
Fat mass index in adolescence (kg/m^2)	≤28 weeks	2 trials	16	-0.48 (-2.78, 1.83)	0.66	0.69	0.58
	29 to 32 weeks	2 trials	66	0.27 (-1.13, 1.66)	0.70	0.009	
	33 to 36 weeks	2 trials	16	1.30 (-1.13, 3.73)	0.26	N/A	
Fat mass index at >3 years (kg/m^2)	≤28 weeks	4 trials	92	0.40 (-0.20, 1.01)	0.19	0.10	0.37
	29 to 32 weeks	4 trials	212	-0.08 (-0.67, 0.51)	0.78	0.006	
	33 to 36 weeks	3 trials	38	0.77 (-0.72, 2.26)	0.30	0.46	
Percent fat mass in infancy (%)	≤28 weeks	8 trials	247	-1.45 (-3.05, 0.16)	0.08	0.87	0.11
	29 to 32 weeks	7 trials	301	-0.21 (-1.69, 1.27)	0.78	0.001	
	33 to 36 weeks	2 trials	19	5.58 (-5.01, 16.17)	0.28	0.21	
Percent fat mass in childhood (%)	≤28 weeks	2 trials	76	3.10 (-0.20, 6.39)	0.07	0.03	0.14
	29 to 32 weeks	2 trials	146	-1.58 (-4.92, 1.77)	0.35	0.74	
	33 to 36 weeks	1 trial	22	5.19 (-9.37, 19.74)	0.46	N/A	
Percent fat mass in adolescence (%)	≤28 weeks	2 trials	16	-2.64 (-11.68, 6.41)	0.54	0.81	0.71
	29 to 32 weeks	2 trials	66	-0.33 (-4.67, 4.00)	0.88	0.02	
	33 to 36 weeks	2 trials	16	1.49 (-5.89, 8.87)	0.67	N/A	
Percent fat mass at >3 years (%)	≤28 weeks	4 trials	92	2.25 (-0.80, 5.31)	0.15	0.09	0.19
	29 to 32 weeks	4 trials	212	-1.17 (-3.82, 1.48)	0.38	0.17	
	33 to 36 weeks	3 trials	38	3.96 (-5.09, 13.01)	0.38	0.88	
	≤28 weeks	8 trials	247	0.23 (-0.03, 0.49)	0.08	0.49	0.60

Outcome	Subgroup	No. of trials	No. of participants	aMD (95% CI)	P for overall effect	P for heterogeneity	P for subgroup interaction
Lean mass in infancy (kg)	29 to 32 weeks	7 trials	303	0.08 (-0.10, 0.25)	0.39	0.22	
	33 to 36 weeks	2 trials	19	0.06 (-0.76, 0.89)	0.87	0.67	
Lean mass in childhood (kg)	≤28 weeks	2 trials	76	-0.33 (-1.31, 0.65)	0.51	0.10	0.49
	29 to 32 weeks	2 trials	146	-0.09 (-1.00, 0.82)	0.84	0.43	
	33 to 36 weeks	1 trial	22	-1.97 (-5.49, 1.55)	0.25	N/A	
Lean mass in adolescence (kg)	≤28 weeks	2 trials	16	-1.50 (-5.37, 2.36)	0.41	0.56	0.29
	29 to 32 weeks	2 trials	66	-0.63 (-3.23, 1.97)	0.63	0.28	
	33 to 36 weeks	2 trials	16	3.37 (-4.87, 11.61)	0.39	N/A	
Lean mass at >3 years (kg)	≤28 weeks	4 trials	92	-0.54 (-1.51, 0.44)	0.28	0.21	0.92
	29 to 32 weeks	4 trials	212	-0.29 (-1.30, 0.71)	0.57	0.40	
	33 to 36 weeks	3 trials	38	-0.49 (-3.91, 2.92)	0.77	0.29	
Lean mass index in infancy (kg/m²)	≤28 weeks	8 trials	246	0.30 (-0.05, 0.64)	0.09	0.77	0.71
	29 to 32 weeks	7 trials	301	0.12 (-0.21, 0.44)	0.48	0.27	
	33 to 36 weeks	2 trials	19	-0.15 (-2.85, 2.55)	0.91	0.66	
Lean mass index in childhood (kg/m²)	≤28 weeks	2 trials	76	-0.17 (-0.64, 0.29)	0.47	0.05	0.13
	29 to 32 weeks	2 trials	146	0.01 (-0.49, 0.52)	0.96	0.20	
	33 to 36 weeks	1 trial	22	-1.54 (-3.76, 0.69)	0.17	N/A	
Lean mass index in adolescence (kg/m²)	≤28 weeks	2 trials	16	-0.47 (-1.61, 0.67)	0.38	0.32	0.48
	29 to 32 weeks	2 trials	66	-0.29 (-1.27, 0.69)	0.56	0.02	
	33 to 36 weeks	2 trials	16	1.50 (-0.42, 3.41)	0.11	N/A	
Lean mass index at >3 years (kg/m²)	≤28 weeks	4 trials	92	-0.25 (-0.66, 0.18)	0.25	0.10	0.75
	29 to 32 weeks	4 trials	212	-0.09 (-0.55, 0.37)	0.69	0.02	
	33 to 36 weeks	3 trials	38	-0.45 (-2.00, 1.09)	0.55	0.15	
**Bone development**							
BMC in infancy (g)	≤28 weeks	6 trials	153	15.04 (-0.40, 30.49)	0.06	0.62	0.50
	29 to 32 weeks	5 trials	141	4.71 (-9.16, 18.59)	0.50	0.45	
	33 to 36 weeks	2 trials	19	6.95 (-16.57, 30.47)	0.54	0.67	
BMC in adolescence (g)	≤28 weeks	2 trials	16	-58.61 (-281.74, 164.52)	0.58	0.54	0.50
	29 to 32 weeks	2 trials	66	65.64 (-173.23, 89.29)	0.53	0.70	
	33 to 36 weeks	2 trials	16	-25.73 (-348.42, 296.97)	0.86	N/A	
BMD in infancy	≤28 weeks	5 trials	137	0.007 (-0.006, 0.021)	0.27	0.69	0.96

Outcome	Subgroup	No. of trials	No. of participants	aMD (95% CI)	P for overall effect	P for heterogeneity	P for subgroup interaction
	29 to 32 weeks	4 trials	111	0.006 (-0.009, 0.021)	0.42	0.21	
	33 to 36 weeks	2 trials	19	0.010 (-0.029, 0.048)	0.61	0.95	
BMD in adolescence	≤28 weeks	2 trials	16	-0.040 (-0.121, 0.042)	0.31	0.18	0.83
	29 to 32 weeks	2 trials	66	-0.006 (-0.041, 0.029)	0.72	0.80	
	33 to 36 weeks	2 trials	16	-0.050 (-0.141, 0.040)	0.25	N/A	
Abbreviation: BMI: body mass index; HC: head circumference; BMC: bone mineral content; BMD: bone mineral density; aMD: adjusted mean difference; N/A: not applicable. Mean differences were adjusted for sex and birthweight z-scores.							

Table S6. Subgroup analyses of timing of supplements.

Outcome	Subgroup	No. of trials	No. of participants	aMD (95% CI)	P for overall effect	P for heterogeneity	P for subgroup interaction
**BMI**							
BMI in childhood (kg/m^2)	In hospital	1 trial	55	-0.61 (-1.80, 0.57)	0.30	N/A	0.23
	Post discharge	2 trials	278	-0.01 (-0.37, 0.35)	0.97	0.46	
BMI in infancy (kg/m^2)	In hospital	9 trials	622	0.02 (-0.21, 0.25)	0.89	0.88	0.93
	Post discharge	11 trials	1386	0.03 (-0.12, 0.18)	0.70	0.13	
BMI in toddlers (kg/m^2)	In hospital	5 trials	402	-0.06 (-0.31, 0.19)	0.64	0.81	0.91
	Post discharge	5 trials	792	-0.08 (-0.25, 0.10)	0.40	0.36	
BMI in adolescence (kg/m^2)	In hospital	1 trial	36	-2.49 (-4.51, -0.48)	0.02	N/A	0.02
	Post discharge	1 trial	67	1.40 (-0.48, 3.27)	0.14	N/A	
BMI at >3 years (kg/m^2)	In hospital	2 trials	91	-1.17 (-2.25, -0.10)	0.03	0.18	0.01
	Post discharge	3 trials	345	0.26 (-0.20, 0.71)	0.27	0.06	
BMI z-scores in infancy	In hospital	9 trials	622	-0.008 (-0.17, 0.16)	0.92	0.96	0.70
	Post discharge	11 trials	1386	0.03 (-0.08, 0.14)	0.59	0.07	
BMI z-scores in toddlers	In hospital	5 trials	402	-0.07 (-0.25, 0.12)	0.48	0.77	0.94
	Post discharge	5 trials	792	-0.08 (-0.21, 0.06)	0.26	0.43	
BMI z-scores in childhood	In hospital	1 trial	55	-0.29 (-1.01, 0.43)	0.43	N/A	0.48
	Post discharge	2 trials	278	-0.06 (-0.29, 0.18)	0.63	0.51	

Outcome	Subgroup	No. of trials	No. of participants	aMD (95% CI)	P for overall effect	P for heterogeneity	P for subgroup interaction
BMI z-scores in adolescence	In hospital	1 trial	36	-1.20 (-2.08, -0.31)	0.01	N/A	0.01
	Post discharge	1 trial	67	0.47 (-0.17, 1.11)	0.15	N/A	
BMI z-scores at >3 years	In hospital	2 trials	91	-0.55 (-1.11, 0.02)	0.06	0.22	0.02
	Post discharge	3 trials	345	0.04 (-0.18, 0.26)	0.73	0.18	
**Weight**							
Weight in infancy (kg)	In hospital	9 trials	644	-0.09 (-0.28, 0.10)	0.36	0.63	0.03
	Post discharge	11 trials	1388	0.18 (0.04, 0.30)	0.01	0.004	
Weight in toddlers (kg)	In hospital	5 trials	408	-0.08 (-0.34, 0.17)	0.53	0.88	0.40
	Post discharge	5 trials	796	0.05 (-0.13, 0.24)	0.58	0.53	
Weight in childhood (kg)	In hospital	1 trial	55	-1.74 (-3.61, 0.13)	0.07	N/A	0.14
	Post discharge	2 trials	278	-0.21 (-1.03, 0.61)	0.61	0.43	
Weight in adolescence (kg)	In hospital	1 trial	36	-4.58 (-11.46, 2.30)	0.19	N/A	0.11
	Post discharge	1 trial	67	2.31 (-2.59, 7.21)	0.35	N/A	
Weight at >3 years (kg)	In hospital	2 trials	91	-2.84 (-5.22, -0.46)	0.02	0.25	0.02
	Post discharge	3 trials	345	0.29 (-0.92, 1.50)	0.64	0.19	
Weight z-scores in infancy	In hospital	9 trials	644	-0.02 (-0.18, 0.15)	0.85	0.65	0.23
	Post discharge	11 trials	1388	0.11 (-0.01, 0.22)	0.06	0.003	
Weight z-scores in toddlers	In hospital	5 trials	408	-0.09 (-0.27, 0.09)	0.33	0.80	0.13
	Post discharge	5 trials	796	0.07 (-0.05, 0.22)	0.21	0.55	
Weight z-scores in childhood	In hospital	1 trial	53	-0.33 (-0.88, 0.23)	0.25	N/A	0.63
	Post discharge	2 trials	278	-0.18 (-0.42, 0.06)	0.14	0.74	
**Length/ height**							
Length in infancy (cm)	In hospital	9 trials	622	-0.38 (-1.01, 0.25)	0.23	0.54	0.005
	Post discharge	11 trials	1386	0.70 (0.27, 1.12)	0.001	0.009	
Height in toddlers (cm)	In hospital	5 trials	402	-0.16 (-0.89, 0.58)	0.68	0.09	0.24
	Post discharge	5 trials	796	0.38 (-0.14, 0.90)	0.15	0.28	
Height in childhood (cm)	In hospital	1 trial	55	-2.35 (-5.21, 0.51)	0.11	N/A	0.32
	Post discharge	2 trials	279	-0.76 (-2.02, 0.50)	0.24	0.73	
Height in adolescence (cm)	In hospital	1 trial	36	-1.90 (-8.05, 4.25)	0.54	N/A	0.73
	Post discharge	1 trial	67	-0.56 (-4.94, 3.82)	0.80	N/A	
Height at >3 years (cm)	In hospital	2 trials	91	-2.29 (-4.94, 0.36)	0.09	0.96	0.30
	Post discharge	3 trials	346	-0.73 (-2.07, 0.61)	0.29	0.94	
	In hospital	9 trials	622	-0.04 (-0.23, 0.14)	0.66	0.27	0.10

Outcome	Subgroup	No. of trials	No. of participants	aMD (95% CI)	P for overall effect	P for heterogeneity	P for subgroup interaction
Length z-scores in infancy	Post discharge	11 trials	1386	0.14 (0.02, 0.27)	0.02	0.0024	
Height z-scores in toddlers	In hospital	5 trials	402	-0.07 (-0.28, 0.14)	0.50	0.08	0.03
	Post discharge	5 trials	796	0.21 (0.07, 0.36)	0.005	0.52	
Height z-scores in childhood	In hospital	1 trial	55	0.03 (-0.48, 0.53)	0.92	N/A	0.34
	Post discharge	2 trials	279	-0.24 (-0.46, -0.02)	0.03	0.83	
Height z-scores in adolescence	In hospital	1 trial	36	-0.44 (-1.13, 0.26)	0.22	N/A	0.19
	Post discharge	1 trial	67	0.14 (-0.36, 0.63)	0.59	N/A	
Height z-scores at >3 years	In hospital	2 trials	91	-0.14 (-0.53, 0.26)	0.50	0.33	0.89
	Post discharge	3 trials	346	-0.17 (-0.37, 0.04)	0.11	0.33	
Weight for length z-scores in infancy	In hospital	9 trials	622	-0.002 (-0.17, 0.16)	0.98	0.99	0.78
	Post discharge	11 trials	1386	0.03 (-0.08, 0.14)	0.65	0.06	
Weight for length z-scores in toddlers	In hospital	5 trials	402	-0.07 (-0.26. 0.11)	0.43	0.95	0.68
	Post discharge	5 trials	796	-0.03 (-0.16, 0.11)	0.69	0.32	
**HC**							
HC in infancy (cm)	In hospital	9 trials	649	-0.20 (-0.51, 0.11)	0.20	0.35	0.12
	Post discharge	11 trials	1385	0.10 (-0.11, 0.31)	0.35	0.14	
HC in toddlers (cm)	In hospital	6 trials	449	-0.14 (-0.44, 0.15)	0.34	0.54	0.88
	Post discharge	5 trials	777	-0.12 (-0.34, 0.11)	0.31	0.21	
HC in childhood (cm)	In hospital	1 trial	53	0.12 (-0.77, 1.01)	0.79	N/A	0.40
	Post discharge	2 trials	264	-0.30 (-0.70, 0.10)	0.14	0.63	
HC z-scores in infancy	In hospital	9 trials	649	-0.009 (-0.19, 0.17)	0.92	0.29	0.96
	Post discharge	11 trials	1385	-0.004 (-0.13, 0.12)	0.96	0.09	
HC z-scores in toddlers	In hospital	6 trials	449	-0.12 (-0.33, 0.09)	0.25	0.48	0.67
	Post discharge	5 trials	777	-0.07 (-0.22, 0.10)	0.43	0.19	
**Body composition**							
Fat mass in infancy (kg)	In hospital	2 trials	84	0.06 (-0.18, 0.31)	0.62	0.42	0.64
	Post discharge	6 trials	485	-0.001 (-0.10, 0.10)	0.98	0.01	
Fat mass in adolescence (kg)	In hospital	1 trial	33	-3.14 (-7.40, 1.23)	0.15	N/A	0.06
	Post discharge	1 trial	65	1.92 (-1.01, 4.84)	0.20	N/A	

Outcome	Subgroup	No. of trials	No. of participants	aMD (95% CI)	P for overall effect	P for heterogeneity	P for subgroup interaction
Fat mass at >3 years (kg)	In hospital	1 trial	33	-2.80 (-5.42, -0.18)	0.04	N/A	0.02
	Post discharge	3 trials	309	0.52 (-0.32, 1.37)	0.23	0.14	
Fat mass index in infancy (kg/m²)	In hospital	2 trials	84	0.14 (-0.40, 0.68)	0.60	0.52	0.52
	Post discharge	6 trials	482	-0.05 (-0.27, 0.18)	0.68	0.02	
Fat mass index in adolescence (kg/m²)	In hospital	1 trial	33	-1.65 (-3.48, 0.17)	0.07	N/A	0.02
	Post discharge	1 trial	65	1.10 (-0.15, 2.35)	0.08	N/A	
Fat mass index at >3 years (kg/m²)	In hospital	1 trial	33	-1.45 (-2.81, -0.08)	0.04	N/A	0.02
	Post discharge	3 trials	309	0.32 (-0.12, 0.76)	0.16	0.11	
Percent fat mass in infancy (%)	In hospital	2 trials	84	0.28 (-2.59, 3.15)	0.85	0.86	0.56
	Post discharge	6 trials	483	-0.65 (-1.82, 0.53)	0.28	0.04	
Percent fat mass in adolescence (%)	In hospital	1 trial	33	-5.98 (-11.78, -0.18)	0.04	N/A	0.03
	Post discharge	1 trial	65	2.03 (-1.95, 6.02)	0.31	N/A	
Percent fat mass at >3 years (%)	In hospital	1 trial	33	-5.17 (-11.71, 1.38)	0.12	N/A	0.08
	Post discharge	3 trials	309	0.93 (-1.18, 3.04)	0.39	0.63	
Lean mass in infancy (kg)	In hospital	2 trials	84	0.10 (-0.29, 0.49)	0.61	0.03	0.82
	Post discharge	6 trials	485	0.15 (-0.01, 0.31)	0.06	0.10	
Lean mass in adolescence (kg)	In hospital	1 trial	33	-1.33 (-5.01, 2.35)	0.48	N/A	0.49
	Post discharge	1 trial	65	0.24 (-2.28, 2.77)	0.85	N/A	
Lean mass at >3 years (kg)	In hospital	1 trial	33	-1.54 (-3.99, 0.90)	0.22	N/A	0.31
	Post discharge	3 trials	309	-0.21 (-1.00, 0.58)	0.60	0.65	
Lean mass index in infancy (kg/m²)	In hospital	2 trials	84	0.20 (-0.46, 0.84)	0.55	0.14	0.96
	Post discharge	6 trials	482	0.22 (-0.05, 0.48)	0.11	0.25	
Lean mass index in adolescence (kg/m²)	In hospital	1 trial	33	-1.19 (-2.47, 0.09)	0.07	N/A	0.05
	Post discharge	1 trial	65	0.37 (-0.51, 1.25)	0.41	N/A	
Lean mass index at >3 years (kg/m²)	In hospital	1 trial	33	-1.33 (-2.43, -0.23)	0.02	N/A	0.03
	Post discharge	3 trials	309	-0.06 (-0.42, 0.29)	0.72	0.29	

Outcome	Subgroup	No. of trials	No. of participants	aMD (95% CI)	P for overall effect	P for heterogeneity	P for subgroup interaction
**Bone development**							
BMC in infancy (g)	In hospital	1 trial	40	-2.80 (-30.01, 24.41)	0.84	N/A	0.32
	Post discharge	5 trials	273	12.08 (1.72, 22.44)	0.02	0.24	
BMC in adolescence (g)	In hospital	1 trial	33	-20.18 (-204.06, 163.71)	0.83	N/A	0.92
	Post discharge	1 trial	65	-31.23 (-157.39, 94.93)	0.62	N/A	
BMD in infancy	In hospital	1 trial	40	0.007 (-0.017, 0.03)	0.58	N/A	0.92
	Post discharge	4 trials	227	0.008 (-0.002, 0.18)	0.11	0.61	
BMD in adolescence	In hospital	1 trial	33	0.01 (-0.04, 0.06)	0.70	N/A	0.36
	Post discharge	1 trial	65	-0.02 (-0.054, 0.017)	0.28	N/A	

Abbreviation: BMI: body mass index; HC: head circumference; BMC: bone mineral content; BMD: bone mineral density; aMD: adjusted mean difference; N/A: not applicable.

Mean differences were adjusted for sex, gestational age and birthweight z-scores.

Table S7. Subgroup analyses of type of supplement

Outcome	Subgroup	No. of trials	No. of participants	aMD (95% CI)	P for overall effect	P for heterogeneity	P for subgroup interaction
**BMI**							
BMI in childhood (kg/m²)	Protein	1 trial	55	-0.61 (-1.80, 0.57)	0.30	N/A	0.23
	Multicomponent	2 trials	278	-0.01 (-0.37, 0.35)	0.97	0.46	
BMI in infancy (kg/m²)	Protein	3 trials	150	0.02 (-0.45, 0.49)	0.94	0.69	0.98
	Multicomponent	15 trials	1858	0.03 (-0.11, 0.16)	0.70	0.26	
BMI in toddlers (kg/m²)	Protein	2 trials	56	-0.09 (-0.77, 0.58)	0.78	0.47	0.95
	Multicomponent	8 trials	1138	-0.07 (-0.22, 0.08)	0.35	0.70	
BMI in adolescence (kg/m²)	Protein	1 trial	36	-2.49 (-4.51, -0.48)	0.02	N/A	0.02
	Multicomponent	1 trial	67	1.40 (-0.48, 3.27)	0.14	N/A	
BMI at >3 years (kg/m²)	Protein	2 trials	91	-1.17 (-2.25, -0.10)	0.03	0.18	0.01
	Multicomponent	3 trials	345	0.26 (-0.20, 0.71)	0.27	0.06	
BMI z-scores in infancy	Protein	3 trials	150	-0.002 (-0.34, 0.34)	0.99	0.79	0.90
	Multicomponent	15 trials	1858	0.02 (-0.08, 0.12)	0.69	0.20	
BMI z-scores in toddlers	Protein	2 trials	56	-0.11 (-0.62, 0.39)	0.67	0.46	0.88
	Multicomponent	8 trials	1138	-0.07 (-0.18, 0.04)	0.21	0.76	
BMI z-scores in childhood	Protein	1 trial	55	-0.29 (-1.01, 0.43)	0.43	N/A	0.48
	Multicomponent	2 trials	278	-0.06 (-0.29, 0.18)	0.63	0.51	
BMI z-scores in adolescence	Protein	1 trial	36	-1.20 (-2.08, -0.31)	0.01	N/A	0.01
	Multicomponent	1 trial	67	0.47 (-0.17, 1.11)	0.15	N/A	
BMI z-scores at >3 years	Protein	2 trials	91	-0.55 (-1.11, 0.02)	0.06	0.22	0.02
	Multicomponent	3 trials	345	0.04 (-0.18, 0.26)	0.73	0.18	
**Weight**							
	Protein	3 trials	163	-0.10 (-0.48, 0.27)	0.60	0.83	0.30

Outcome	Subgroup	No. of trials	No. of participants	aMD (95% CI)	P for overall effect	P for heterogeneity	P for subgroup interaction
Weight in infancy (kg)	Multicomponent	15 trials	1869	0.11 (-0.005, 0.22)	0.06	0.002	
Weight in toddlers (kg)	Protein	2 trials	58	0.032 (-0.72, 0.66)	0.93	0.31	0.91
	Multicomponent	8 trials	1146	0.008 (0.14, 0.16)	0.92	0.73	
Weight in childhood (kg)	Protein	1 trial	55	-1.74 (-3.61, 0.13)	0.07	N/A	0.14
	Multicomponent	2 trials	278	-0.21 (-1.03, 0.61)	0.61	0.43	
Weight in adolescence (kg)	Protein	1 trial	36	-4.58 (-11.46, 2.30)	0.19	N/A	0.11
	Multicomponent	1 trial	67	2.31 (-2.59, 7.21)	0.35	N/A	
Weight at >3 years (kg)	Protein	2 trials	91	-2.84 (-5.22, -0.46)	0.02	0.25	0.02
	Multicomponent	3 trials	345	0.29 (-0.92, 1.50)	0.64	0.19	
Weight z-scores in infancy	Protein	3 trials	163	0.01 (-0.32, 0.33)	0.97	0.98	0.71
	Multicomponent	15 trials	1869	0.07 (-0.03, 0.17)	0.14	0.004	
Weight z-scores in toddlers	Protein	2 trials	58	-0.22 (-0.72, 0.27)	0.38	0.50	0.31
	Multicomponent	8 trials	1146	0.04 (-0.07, 0.15)	0.51	0.65	
Weight z-scores in childhood	Protein	1 trial	53	-0.33 (-0.88, 0.23)	0.25	N/A	0.63
	Multicomponent	2 trials	278	-0.18 (-0.42, 0.06)	0.14	0.74	
**Length/ height**							
Length in infancy (cm)	Protein	3 trials	150	-0.47 (-1.75, 0.82)	0.48	0.58	0.19
	Multicomponent	15 trials	1858	0.43 (0.06, 0.79)	0.02	0.002	
Height in toddlers (cm)	Protein	2 trials	56	0.24 (-1.76, 2.25)	0.81	0.05	0.97
	Multicomponent	8 trials	1142	0.20 (-0.24, 0.63)	0.37	0.20	
Height in childhood (cm)	Protein	1 trial	55	-2.35 (-5.21, 0.51)	0.11	N/A	0.32
	Multicomponent	2 trials	279	-0.76 (-2.02, 0.50)	0.24	0.73	
Height in adolescence (cm)	Protein	1 trial	36	-1.90 (-8.05, 4.25)	0.54	N/A	0.73
	Multicomponent	1 trial	67	-0.56 (-4.94, 3.82)	0.80	N/A	

Outcome	Subgroup	No. of trials	No. of participants	aMD (95% CI)	P for overall effect	P for heterogeneity	P for subgroup interaction
Height at >3 years (cm)	Protein	2 trials	91	-2.29 (-4.94, 0.36)	0.09	0.96	0.30
	Multicomponent	3 trials	346	-0.73 (-2.07, 0.61)	0.29	0.94	
Length z-scores in infancy	Protein	3 trials	150	-0.07 (-0.45, 0.31)	0.71	0.67	0.40
	Multicomponent	15 trials	1858	0.10 (-0.01, 0.21)	0.07	0.001	
Height z-scores in toddlers	Protein	2 trials	56	-0.24 (-0.80, 0.33)	0.41	0.04	0.21
	Multicomponent	8 trials	1142	0.14 (0.01, 0.26)	0.03	0.16	
Height z-scores in childhood	Protein	1 trial	55	0.03 (-0.48, 0.53)	0.92	N/A	0.34
	Multicomponent	2 trials	279	-0.24 (-0.46, -0.02)	0.03	0.83	
Height z-scores in adolescence	Protein	1 trial	36	-0.44 (-1.13, 0.26)	0.22	N/A	0.19
	Multicomponent	1 trial	67	0.14 (-0.36, 0.63)	0.59	N/A	
Height z-scores at >3 years	Protein	2 trials	91	-0.14 (-0.53, 0.26)	0.50	0.33	0.89
	Multicomponent	3 trials	346	-0.17 (-0.37, 0.04)	0.11	0.33	
Weight for length z-scores in infancy	Protein	3 trials	150	0.02 (-0.32, 0.36)	0.91	0.91	0.99
	Multicomponent	15 trials	1858	0.02 (-0.08, 0.11)	0.73	0.22	
Weight for length z-scores in toddlers	Protein	2 trials	56	-0.12 (-0.63, 0.39)	0.64	0.75	0.76
	Multicomponent	8 trials	1138	-0.04 (-0.15, 0.07)	0.48	0.69	
**HC**							
HC in infancy (cm)	Protein	3 trials	163	-0.27 (-0.88, 0.34)	0.38	0.59	0.36
	Multicomponent	15 trials	1871	0.03 (-0.15, 0.21)	0.76	0.07	
HC in toddlers (cm)	Protein	2 trials	58	-0.48 (-1.31, 0.35)	0.26	0.79	0.40
	Multicomponent	9 trials	1168	-0.11 (-0.29, 0.07)	0.24	0.30	
HC in childhood (cm)	Protein	1 trial	53	0.12 (-0.77, 1.01)	0.79	N/A	0.40
	Multicomponent	2 trials	264	-0.30 (-0.70, 0.10)	0.14	0.63	
	Protein	3 trials	163	-0.06 (-0.42, 0.31)	0.76	0.64	0.77

Outcome	Subgroup	No. of trials	No. of participants	aMD (95% CI)	P for overall effect	P for heterogeneity	P for subgroup interaction
HC z-scores in infancy	Multicomponent	15 trials	1871	-0.001 (-0.11, 0.11)	0.99	0.06	
HC z-scores in toddlers	Protein	2 trials	58	-0.50 (-1.08, 0.09)	0.10	0.53	0.16
	Multicomponent	9 trials	1168	-0.07 (-0.20, 0.06)	0.32	0.37	
**Body composition**							
Fat mass in infancy (kg)	Protein	2 trials	92	0.04 (-0.19, 0.27)	0.74	0.93	0.77
	Multicomponent	6 trials	477	0.001 (-0.10, 0.10)	0.98	0.008	
Fat mass in adolescence (kg)	Protein	1 trial	33	-3.14 (-7.40, 1.23)	0.15	N/A	0.06
	Multicomponent	1 trial	65	1.92 (-1.01, 4.84)	0.20	N/A	
Fat mass at >3 years (kg)	Protein	1 trial	33	-2.80 (-5.42, -0.18)	0.04	N/A	0.02
	Multicomponent	3 trials	309	0.52 (-0.32, 1.37)	0.23	0.14	
Fat mass index in infancy (kg/m²)	Protein	2 trials	92	-0.08 (-0.59, 0.43)	0.76	0.57	0.81
	Multicomponent	6 trials	474	-0.01 (-0.24, 0.21)	0.92	0.01	
Fat mass index in adolescence (kg/m²)	Protein	1 trial	33	-1.65 (-3.48, 0.17)	0.07	N/A	0.02
	Multicomponent	1 trial	65	1.10 (-0.15, 2.35)	0.08	N/A	
Fat mass index at >3 years (kg/m²)	Protein	1 trial	33	-1.45 (-2.81, -0.08)	0.04	N/A	0.02
	Multicomponent	3 trials	309	0.32 (-0.12, 0.76)	0.16	0.11	
Percent fat mass in infancy (%)	Protein	2 trials	92	-0.50 (-3.20, 2.20)	0.72	0.49	0.98
	Multicomponent	6 trials	475	-0.55 (-1.73, 0.64)	0.37	0.02	
Percent fat mass in adolescence (%)	Protein	1 trial	33	-5.98 (-11.78, -0.18)	0.04	N/A	0.03
	Multicomponent	1 trial	65	2.03 (-1.95, 6.02)	0.31	N/A	
Percent fat mass at >3 years (%)	Protein	1 trial	33	-5.17 (-11.71, 1.38)	0.12	N/A	0.08
	Multicomponent	3 trials	309	0.93 (-1.18, 3.04)	0.39	0.63	

Outcome	Subgroup	No. of trials	No. of participants	aMD (95% CI)	P for overall effect	P for heterogeneity	P for subgroup interaction
Lean mass in infancy (kg)	Protein	2 trials	92	0.18 (-0.19, 0.55)	0.33	0.30	0.83
	Multicomponent	7 trials	477	0.14 (-0.03, 0.30)	0.10	0.05	
Lean mass in adolescence (kg)	Protein	1 trial	33	-1.33 (-5.01, 2.35)	0.48	N/A	0.49
	Multicomponent	1 trial	65	0.24 (-2.28, 2.77)	0.85	N/A	
Lean mass at >3 years (kg)	Protein	1 trial	33	-1.54 (-3.99, 0.90)	0.22	N/A	0.31
	Multicomponent	3 trials	309	-0.21 (-1.00, 0.58)	0.60	0.65	
Lean mass index in infancy (kg/m^2)	Protein	2 trials	92	-0.03 (-0.63, 0.57)	0.93	0.77	0.39
	Multicomponent	6 trials	474	0.26 (-0.01, 0.52)	0.06	0.22	
Lean mass index in adolescence (kg/m^2)	Protein	1 trial	33	-1.19 (-2.47, 0.09)	0.07	N/A	0.05
	Multicomponent	1 trial	65	0.37 (-0.51, 1.25)	0.41	N/A	
Lean mass index at >3 years (kg/m^2)	Protein	1 trial	33	-1.33 (-2.43, -0.23)	0.02	N/A	0.03
	Multicomponent	3 trials	309	-0.06 (-0.42, 0.29)	0.72	0.29	
**Bone development**							
BMC in infancy (g)	Protein	2 trials	92	13.10 (-4.88, 31.08)	0.15	0.19	0.71
	Multicomponent	4 trials	221	8.99 (-2.51, 20.49)	0.13	0.20	
BMC in adolescence (g)	Protein	1 trial	33	-20.18 (-204.06, 163.71)	0.83	N/A	0.92
	Multicomponent	1 trial	65	-31.23 (-157.39, 94.93)	0.62	N/A	
BMD in infancy	Protein	2 trials	92	0.011 (-0.005, 0.027)	0.16	0.65	0.59
	Multicomponent	3 trials	175	0.006 (-0.005, 0.017)	0.31	0.53	
	Protein	1 trial	33	0.01 (-0.04, 0.06)	0.70	N/A	0.36

Outcome	Subgroup	No. of trials	No. of participants	aMD (95% CI)	P for overall effect	P for heterogeneity	P for subgroup interaction
BMD in adolescence	Multicomponent	1 trial	65	-0.02 (-0.054, 0.017)	0.28	N/A	
Abbreviation: BMI: body mass index; HC: head circumference; BMC: bone mineral content; BMD: bone mineral density; aMD: adjusted mean difference; N/A: not applicable. Mean differences were adjusted for sex, gestational age and birthweight z-scores.							

Table S8. Subgroup analyses of primary milk feed.

Outcome	Subgroup	No. of trials	No. of participants	aMD (95% CI)	P for overall effect	P for heterogeneity	P for subgroup interaction
**BMI**							
BMI in childhood (kg/m^2)	BM	2 trials	206	-0.26 (-0.71, 0.19)	0.25	0.42	0.26
	Formula	1 trial	127	0.14 (-0.47, 0.75)	0.65	N/A	
BMI in infancy (kg/m^2)	BM	5 trials	670	0.02 (-0.21, 0.25)	0.88	0.77	0.88
	Formula	11 trials	1231	0.04 (-0.12, 0.19)	0.67	0.11	
	PN+EN	2 trials	107	-0.13 (-0.70, 0.44)	0.65	0.79	
BMI in toddlers (kg/m^2)	BM	4 trials	526	-0.04 (-0.26, 0.19)	0.76	0.91	0.66
	Formula	5 trials	634	-0.10 (-0.29, 0.09)	0.31	0.28	
	PN+EN	1 trial	34	-0.63 (-1.66, 0.42)	0.22	N/A	
BMI in adolescence (kg/m^2)	BM	none					N/A
	Formula	2 trials	103	0.14 (-1.27, 1.56)	0.84	0.02	
BMI at >3 years (kg/m^2)	BM	2 trials	206	-0.26 (-0.71, 0.19)	0.25	0.42	0.32
	Formula	3 trials	230	0.14 (-0.56, 0.84)	0.70	0.01	
BMI z-scores in infancy	BM	5 trials	670	-0.008 (-0.18, 0.16)	0.93	0.87	0.77
	Formula	11 trials	1231	0.04 (-0.08, 0.15)	0.53	0.07	
	PN+EN	2 trials	107	-0.11 (-0.50, 0.28)	0.58	0.72	
BMI z-scores in toddlers	BM	4 trials	526	-0.06 (-0.23, 0.11)	0.50	0.89	0.66
	Formula	5 trials	634	-0.08 (-0.23, 0.06)	0.26	0.32	
	PN+EN	1 trial	34	-0.47 (-1.25, 0.33)	0.24	N/A	
BMI z-scores in childhood	BM	2 trials	206	-0.17 (-0.47, 0.12)	0.25	0.72	0.37
	Formula	1 trial	127	0.03 (-0.34, 0.39)	0.88	N/A	
BMI z-scores in adolescence (kg/m^2)	BM	none					N/A
	Formula	2 trials	103	-0.08 (-0.60, 0.44)	0.76	0.01	
BMI z-scores at >3 years	BM	2 trials	206	-0.17 (-0.47, 0.12)	0.25	0.72	0.44
	Formula	3 trials	230	-0.03 (-0.33, 0.27)	0.84	0.01	

Outcome	Subgroup	No. of trials	No. of participants	aMD (95% CI)	P for overall effect	P for heterogeneity	P for subgroup interaction
**Weight**							
Weight in infancy (kg)	BM	5 trials	685	0.05 (-0.14, 0.24)	0.59	0.63	0.33
	Formula	11 trials	1232	0.12 (-0.009, 0.26)	0.07	0.0006	
	PN+EN	2 trials	115	-0.26 (-0.72, 0.19)	0.26	0.36	
Weight in toddlers (kg)	BM	4 trials	530	-0.13 (-0.38, 0.11)	0.29	0.92	0.30
	Formula	5 trials	637	0.08 (-0.10, 0.27)	0.38	0.68	
	PN+EN	1 trial	37	-0.11 (1.11, 0.89)	0.82	N/A	
Weight in childhood (kg)	BM	2 trials	206	-0.85 (-1.81, 0.11)	0.08	0.30	0.21
	Formula	1 trial	127	0.14 (-1.08, 1.37)	0.62	N/A	
Weight in adolescence (kg)	BM	none					N/A
	Formula	2 trials	103	0.01 (-4.01, 4.04)	0.99	0.11	
Weight at >3 years (kg)	BM	2 trials	206	-0.87 (-2.43, 0.71)	0.28	0.30	0.38
	Formula	3 trials	230	0.10 (-1.40, 1.60)	0.89	0.06	
Weight z-scores in infancy	BM	5 trials	685	0.06 (-0.11, 0.23)	0.49	0.81	0.50
	Formula	11 trials	1232	0.08 (-0.04, 0.20)	0.17	0.0026	
	PN+EN	2 trials	115	-0.18 (-0.57, 0.20)	0.35	0.18	
Weight z-scores in toddlers	BM	4 trials	530	-0.08 (-0.25, 0.09)	0.36	0.98	0.28
	Formula	5 trials	637	0.09 (-0.06, 0.23)	0.24	0.42	
	PN+EN	1 trial	37	0.04 (-0.67, 0.75)	0.91	N/A	
Weight z-scores in childhood	BM	2 trials	204	-0.24 (-0.52, 0.03)	0.08	0.81	0.59
	Formula	1 trial	127	-0.12 (-0.47, 0.23)	0.50	N/A	
**Length**							
Length in infancy (cm)	BM	5 trials	670	0.008 (-0.62, 0.63)	0.98	0.61	0.16
	Formula	11 trials	1231	0.59 (0.16, 1.03)	0.008	0.001	
	PN+EN	2 trials	107	-0.67 (-2.48, 1.42)	0.47	0.24	
Height in toddlers (cm)	BM	4 trials	526	-0.41 (-1.13, 0.31)	0.27	0.53	0.04
	Formula	5 trials	638	0.55 (0.04, 1.07)	0.04	0.23	
	PN+EN	1 trial	34	0.93 (-1.96, 3.81)	0.52	N/A	
Height in childhood (cm)	BM	2 trials	206	-1.31 (-2.78, 0.16)	0.08	0.44	0.51
	Formula	1 trial	128	-0.51 (-2.38, 1.36)	0.59	N/A	
Height in adolescence (cm)	BM	None					N/A
	Formula	2 trials	103	-1.00 (-4.55, 0.56)	0.58	0.73	
Height at >3 years (cm)	BM	2 trials	206	-1.33 (-3.07, 0.42)	0.14	0.44	0.67
	Formula	3 trials	231	-0.80 (-2.45, 0.86)	0.34	0.78	
Length z-scores in infancy	BM	5 trials	670	0.08 (-0.11, 0.28)	0.41	0.62	0.69
	Formula	11 trials	1231	0.09 (-0.03, 0.22)	0.14	0.001	
	PN+EN	2 trials	107	-0.14 (-0.60, 0.32)	0.55	0.02	
	BM	4 trials	526	-0.07 (-0.26, 0.13)	0.50	0.88	0.02

Outcome	Subgroup	No. of trials	No. of participants	aMD (95% CI)	P for overall effect	P for heterogeneity	P for subgroup interaction
Height z-scores in toddlers	Formula	5 trials	638	0.23 (0.07, 0.39)	0.005	0.07	
	PN+EN	1 trial	34	0.41 (-0.37, 1.18)	0.29	N/A	
Height z-scores in childhood	BM	2 trials	206	-0.16 (-0.42, 0.09)	0.21	0.36	0.64
	Formula	1 trial	128	-0.26 (-0.59, 0.07)	0.12	N/A	
Height z-scores in adolescence (cm)	BM	None					N/A
	Formula	2 trials	103	-0.06 (-0.46, 0.35)	0.79	0.19	
Height z-scores at >3 years	BM	2 trials	206	-0.17 (-0.43, 0.09)	0.21	0.36	0.98
	Formula	3 trials	231	-0.17(-0.42, 0.08)	0.17	0.28	
Weight for length z-scores in infancy	BM	5 trials	670	-0.02 (-0.19, 0.15)	0.82	0.98	0.87
	Formula	11 trials	1231	0.03 (-0.08, 0.14)	0.60	0.06	
	PN+EN	2 trials	107	0.03 (-0.38, 0.43)	0.90	0.65	
Weight for length z-scores in toddlers	BM	4 trials	526	-0.07 (-0.23, 0.10)	0.43	0.94	0.72
	Formula	5 trials	634	-0.02 (-0.17, 0.12)	0.76	0.33	
	PN+EN	1 trial	34	-0.40 (-1.19, 0.39)	0.31	N/A	
**HC**							
HC in infancy (cm)	BM	5 trials	682	-0.12 (-0.40, 0.17)	0.43	0.57	0.05
	Formula	11 trials	1231	0.14 (-0.09, 0.36)	0.23	0.14	
	PN+EN	2 trials	121	-0.67 (-1.48, 0.15)	0.11	0.37	
HC in toddlers (cm)	BM	4 trials	505	-0.29 (-0.55, -0.03)	0.03	0.82	0.23
	Formula	5 trials	638	-0.09 (-0.33, 0.16)	0.50	0.28	
	PN+EN	2 trial	83	0.25 (-0.67, 1.17)	0.59	0.21	
HC in childhood (cm)	BM	2 trials	190	-0.26 (-0.72, 0.21)	0.28	0.27	0.82
	Formula	1 trial	128	-0.17 (-0.75, 0.40)	0.56	N/A	
HC z-scores in infancy	BM	5 trials	682	-0.001 (-0.17, 0.16)	0.99	0.61	0.70
	Formula	11 trials	1231	0.009 (-0.13, 0.15)	0.90	0.10	
	PN+EN	2 trials	121	-0.11 (-0.57, 0.34)	0.62	0.03	
HC z-scores in toddlers	BM	4 trials	505	-0.21 (-0.39, -0.03)	0.03	0.60	0.24
	Formula	5 trials	638	-0.04 (-0.21, 0.14)	0.69	0.22	
	PN+EN	2 trials	83	0.17 (-0.45, 0.79)	0.59	0.29	
**Body composition**							
Fat mass in infancy (kg)	BM	1 trial	44	0.14 (-0.20, 0.49)	0.42	N/A	0.42
	Formula	7 trials	525	-0.003 (-0.10, 0.09)	0.96	0.02	
Fat mass in childhood (kg)	BM	1 trial	126	-0.11 (-0.97, 0.75)	0.80	N/A	0.67
	Formula	1 trial	118	0.38 (-0.50, 1.27)	0.40	N/A	
Fat mass in adolescence (kg)	BM	None					N/A
	Formula	2 trials	98	0.33 (-2.11, 2.77)	0.79	0.06	
	BM	1 trial	126	-0.14 (-1.47, 1.20)	0.84	N/A	0.53

Outcome	Subgroup	No. of trials	No. of participants	aMD (95% CI)	P for overall effect	P for heterogeneity	P for subgroup interaction
Fat mass at >3 years (kg)	Formula	3 trials	216	0.40 (-0.62, 1.42)	0.44	0.04	
Fat mass index in infancy (kg/m²)	BM	1 trial	44	0.31 (-0.45, 1.07)	0.43	N/A	0.38
	Formula	7 trials	522	-0.04 (-0.26, 0.17)	0.68		
Fat mass index in childhood (kg/m²)	BM	1 trial	126	-0.07 (-0.66, 0.52)	0.81	N/A	0.43
	Formula	1 trial	118	0.27 (-0.34, 0.89)	0.38	N/A	
Fat mass index in adolescence (kg/m²)	BM	None					
	Formula	2 trials	98	0.25 (-0.80, 1.31)	0.63	0.02	
Fat mass index at >3 years (kg/m²)	BM	1 trial	126	-0.08 (-0.78, 0.61)	0.82	N/A	0.42
	Formula	3 trials	216	0.28 (-0.25, 0.81)	0.30	0.03	
Percent fat mass in infancy (%)	BM	1 trial	44	0.63 (-3.41, 4.66)	0.76	N/A	0.57
	Formula	7 trials	523	-0.60 (-1.73, 0.53)	0.30	0.05	
Percent fat mass in childhood (%)	BM	1 trial	126	-0.34 (-3.84, 3.17)	0.85	N/A	0.45
	Formula	1 trial	118	1.61 (-2.00, 5.22)	0.38	N/A	
Percent fat mass in adolescence (%)	BM	None					N/A
	Formula	2 trials	98	-0.50 (-3.82, 2.82)	0.77	0.03	
Percent fat mass at >3 years (%)	BM	1 trial	126	-0.33 (-3.65, 3.00)	0.85	N/A	0.62
	Formula	3 trials	216	0.74 (-1.80, 3.27)	0.57	0.21	
Lean mass in infancy (kg)	BM	1 trial	44	0.31 (-0.24, 0.85)	0.27	N/A	0.54
	Formula	7 trials	525	0.13 (-0.02, 0.28)	0.09	0.11	
Lean mass in childhood (kg)	BM	1 trial	126	-0.62 (-1.56, 0.32)	0.20	N/A	0.36
	Formula	1 trial	118	0.01 (-0.95, 0.98)	0.98	N/A	
Lean mass in adolescence (kg)	BM	None					N/A
	Formula	2 trials	98	-0.25 (-2.32, 1.82)	0.81	0.49	
Lean mass at >3 years (kg)	BM	1 trial	126	-0.66 (-1.89, 0.58)	0.30	N/A	0.52
	Formula	3 trials	216	-0.15 (-1.09, 0.80)	0.76	0.56	
Lean mass index in infancy (kg/m²)	BM	1 trial	44	0.42 (-0.48, 1.32)	0.36		0.63
	Formula	7 trials	522	0.20 (-0.06, 0.45)	0.13	0.31	
Lean mass index in childhood (kg/m²)	BM	1 trial	126	-0.35 (-0.88, 0.18)	0.20	N/A	0.35
	Formula	1 trial	118	0.01 (-0.53, 0.56)	0.97	N/A	
Lean mass index in adolescence (kg/m²)	BM	None					N/A
	Formula	2 trials	98	-0.12 (-0.86, 0.61)	0.74	0.05	

Outcome	Subgroup	No. of trials	No. of participants	aMD (95% CI)	P for overall effect	P for heterogeneity	P for subgroup interaction
Lean mass index at >3 years (kg/m²)	BM	1 trial	126	-0.36 (-0.92, 0.20)	0.21	N/A	0.44
	Formula	3 trials	216	-0.08 (-0.51, 0.35)	0.71	0.11	
**Bone development**							
BMC in infancy (g)	BM	None					N/A
	Formula	6 trials	313	10.22 (0.52, 19.91)	0.04	0.22	
BMC in adolescence (g)	BM	None					N/A
	Formula	2 trials	98	-27.12 (-130.40, 76.15)	0.60	0.92	
BMD in infancy	BM	None					N/A
	Formula	5 trials	267	0.008 (-0.001, 0.017)	0.10	0.75	
BMD in adolescence	BM	None					N/A
	Formula	2 trials	98	-0.009 (-0.038, 0.020)	0.53	0.36	
Abbreviation: BMI: body mass index; HC: head circumference; BMC: bone mineral content; BMD: bone mineral density; aMD: adjusted mean difference; N/A: not applicable<br>Mean differences were adjusted for sex, gestational age and birthweight z-scores.							

Table S9. Subgroup analyse of different epochs.

Outcome	Subgroup	No. of trials	No. of participants	aMD (95% CI)	P for overall effect	P for heterogeneity	P for subgroup interaction
**BMI**							
BMI in childhood (kg/m²)	Before or in 2000	1 trial	127	0.14 (-0.47, 0.75)	0.65	N/A	0.26
	After 2000	2 trials	206	-0.26 (-0.71, 0.19)	0.25	0.42	
BMI in infancy (kg/m²)	Before or in 2000	7 trials	1071	0.13 (-0.05, 0.30)	0.15	0.55	0.09
	After 2000	11 trials	937	-0.09 (-0.28, 0.10)	0.34	0.50	
BMI in toddlers (kg/m²)	Before or in 2000	5 trials	864	-0.08 (-0.25, 0.09)	0.36	0.47	0.85
	After 2000	5 trials	330	-0.05 (-0.32, 0.23)	0.73	0.75	
BMI in adolescence (kg/m²)	Before or in 2000	1 trial	67	1.40 (-0.48, 3.27)	0.14	N/A	0.02
	After 2000	1 trial	36	-2.49 (-4.51, -0.48)	0.02	N/A	
BMI at >3 years (kg/m²)	Before or in 2000	2 trials	194	0.57 (-0.17, 1.32)	0.13	0.13	0.01
	After 2000	3 trials	242	-0.52 (-1.00, -0.04)	0.03	0.03	
BMI z-scores in infancy	Before or in 2000	7 trials	1071	0.10 (-0.03, 0.23)	0.06	0.61	0.06
	After 2000	11 trials	937	-0.08 (-0.21, 0.06)	0.27	0.43	

Outcome	Subgroup	No. of trials	No. of participants	aMD (95% CI)	P for overall effect	P for heterogeneity	P for subgroup interaction
BMI z-scores in toddlers	Before or in 2000	5 trials	864	-0.07 (-0.20, 0.06)	0.28	0.53	0.93
	After 2000	5 trials	330	-0.08 (-0.29, 0.13)	0.45	0.73	
BMI z-scores in childhood	Before or in 2000	1 trial	127	0.03 (-0.34, 0.39)	0.88	N/A	0.37
	After 2000	2 trials	206	-0.17 (-0.47, 0.12)	0.25	0.72	
BMI z-scores in adolescence	Before or in 2000	1 trial	67	0.47 (-0.17, 1.11)	0.15	N/A	0.006
	After 2000	1 trial	36	-1.20 (-2.08, -0.31)	0.01	N/A	
BMI z-scores at >3 years	Before or in 2000	2 trials	194	0.17 (-0.15, 0.49)	0.30	0.23	0.03
	After 2000	3 trials	242	-0.29 (-0.57, -0.01)	0.04	0.14	
**Weight**							
Weight in infancy (kg)	Before or in 2000	7 trials	1072	0.22 (0.07, 0.36)	0.004	0.48	0.01
	After 2000	11 trials	760	-0.05 (-0.21, 0.11)	0.51	0.022	
Weight in toddlers (kg)	Before or in 2000	5 trials	868	0.06 (-0.11, 0.24)	0.48	0.58	0.24
	After 2000	5 trials	336	-0.14 (-0.42, 0.15)	0.34	0.94	
Weight in childhood (kg)	Before or in 2000	1 trial	127	0.14 (-1.08, 1.37)	0.62	N/A	0.21
	After 2000	2 trials	206	-0.85 (-1.81, 0.11)	0.08	0.30	
Weight in adolescence (kg)	Before or in 2000	1 trial	67	2.31 (-2.59, 7.21)	0.35	N/A	0.11
	After 2000	1 trial	36	-4.58 (-11.46, 2.30)	0.19	N/A	
Weight at >3 years (kg)	Before or in 2000	2 trials	194	0.95 (-0.67, 0.57)	0.25	0.27	0.03
	After 2000	3 trial	242	-1.40 (-2.84, 0.05)	0.06	0.05	
Weight z-scores in infancy	Before or in 2000	7 trials	1072	0.17 (0.04, 0.30)	0.009	0.73	0.02
	After 2000	11 trials	760	-0.05 (-0.18, 0.09)	0.50	0.013	
Weight z-scores in toddlers	Before or in 2000	5 trials	868	0.05 (-0.07, 0.18)	0.40	0.31	0.42
	After 2000	5 trials	336	-0.04 (-0.25, 0.16)	0.67	0.83	
Weight z-scores in childhood	Before or in 2000	1 trial	127	-0.12 (-0.47, 0.23)	0.50	N/A	0.59
	After 2000	2 trials	204	-0.24 (-0.52, 0.03)	0.08	0.81	
Length/ height							

Outcome	Subgroup	No. of trials	No. of participants	aMD (95% CI)	P for overall effect	P for heterogeneity	P for subgroup interaction
Length in infancy (cm)	Before or in 2000	7 trials	1071	0.75 (0.27, 1.23)	0.002	0.18	0.02
	After 2000	11 trials	937	-0.08 (-0.59, 0.44)	0.77	0.0397	
Height in toddlers (cm)	Before or in 2000	5 trials	868	0.41 (-0.09, 0.91)	0.11	0.28	0.13
	After 2000	5 trials	330	-0.34 (-1.15, 0.48)	0.42	0.33	
Height in childhood (cm)	Before or in 2000	1 trial	128	-0.51 (-2.38, 1.36)	0.59	N/A	0.51
	After 2000	2 trials	206	-1.31 (-2.78, 0.16)	0.08	0.44	
Height in adolescence (cm)	Before or in 2000	1 trial	67	-0.56 (-4.94, 3.82)	0.80	N/A	0.73
	After 2000	1 trial	36	-1.90 (-8.05, 4.25)	0.54	N/A	
Height at >3 years (cm)	Before or in 2000	2 trials	195	-0.54 (-2.33, 1.26)	0.56	0.99	0.45
	After 2000	3 trial	242	-1.46 (-3.07, 0.15)	0.08	0.67	
Length z-scores in infancy	Before or in 2000	7 trials	1071	0.16 (0.02, 0.30)	0.024	0.14	0.13
	After 2000	11 trials	937	0.001 (-0.15, 0.15)	0.99	0.0074	
Height z-scores in toddlers	Before or in 2000	5 trials	868	0.17 (0.03, 0.31)	0.02	0.12	0.21
	After 2000	5 trials	330	-0.007 (-0.24, 0.22)	0.95	0.08	
Height z-scores in childhood	Before or in 2000	1 trial	128	-0.26 (-0.59, 0.07)	0.12	N/A	0.64
	After 2000	2 trials	206	-0.16 (-0.42, 0.09)	0.21	0.36	
Height z-scores in adolescence	Before or in 2000	1 trial	67	0.14 (-0.36, 0.63)	0.59	N/A	0.19
	After 2000	1 trial	36	-0.44 (-1.13, 0.26)	0.22	N/A	
Height z-scores at >3 years	Before or in 2000	2 trials	195	-0.13 (-0.40, 0.15)	0.36	0.17	0.75
	After 2000	3 trial	242	-0.19 (-0.43, 0.05)	0.13	0.54	
Weight for length z-scores in infancy	Before or in 2000	7 trials	1071	0.10 (-0.02, 0.23)	0.11	0.66	0.05
	After 2000	11 trials	937	-0.08 (-0.22, 0.05)	0.23	0.53	
Weight for length z-scores in toddlers	Before or in 2000	5 trials	868	-0.03 (-0.16, 0.10)	0.64	0.40	0.72
	After 2000	5 trials	330	-0.07 (-0.28, 0.13)	0.48	0.94	
**HC**							

Outcome	Subgroup	No. of trials	No. of participants	aMD (95% CI)	P for overall effect	P for heterogeneity	P for subgroup interaction
HC in infancy (cm)	Before or in 2000	7 trials	1071	0.18 (-0.06, 0.42)	0.14	0.83	0.04
	After 2000	11 trials	963	-0.19 (-0.44, 0.06)	0.14	0.11	
HC in toddlers (cm)	Before or in 2000	5 trials	868	-0.08 (-0.30, 0.13)	0.44	0.23	0.48
	After 2000	6 trials	358	-0.22 (-0.55, 0.11)	0.18	0.56	
HC in childhood (cm)	Before or in 2000	1 trial	128	-0.17 (-0.75, 0.40)	0.56	N/A	0.82
	After 2000	2 trials	190	-0.26 (-0.72, 0.21)	0.28	0.27	
HC z-scores in infancy	Before or in 2000	7 trials	1071	0.02 (-0.12, 0.16)	0.77	0.68	0.60
	After 2000	11 trials	963	-0.03 (-0.18, 0.12)	0.65	0.02	
HC z-scores in toddlers	Before or in 2000	5 trials	868	-0.06 (-0.21, 0.10)	0.46	0.18	0.47
	After 2000	6 trials	358	-0.16 (-0.39, 0.08)	0.18	0.48	
**Body composition**							
Fat mass in infancy (kg)	Before or in 2000	3 trials	151	0.16 (-0.01, 0.35)	0.07	0.85	0.04
	After 2000	5 trials	418	-0.05 (-0.16, 0.06)	0.36	0.008	
Fat mass in childhood (kg)	Before or in 2000	1 trial	118	0.38 (-0.50, 1.27)	0.40	N/A	0.67
	After 2000	1 trial	126	-0.11 (-0.97, 0.75)	0.80	N/A	
Fat mass in adolescence (kg)	Before or in 2000	1 trial	65	1.92 (-1.01, 4.84)	0.20	N/A	0.06
	After 2000	1 trial	33	-3.14 (-7.40, 1.23)	0.15	N/A	
Fat mass at >3 years (kg)	Before or in 2000	2 trials	183	0.96 (-0.14, 2.06)	0.09	0.21	0.05
	After 2000	2 trials	159	-0.67 (-1.85, 0.51)	0.26	0.02	
Fat mass index in infancy (kg/m^2)	Before or in 2000	3 trials	149	0.19 (-0.21, 0.59)	0.36	0.04	0.23
	After 2000	5 trials	417	-0.10 (-0.33, 0.14)	0.44	0.05	
Fat mass index in childhood (kg/m^2)	Before or in 2000	1 trial	118	0.27 (-0.34, 0.89)	0.38	N/A	0.43
	After 2000	1 trial	126	-0.07 (-0.66, 0.52)	0.81	N/A	
Fat mass index in adolescence (kg/m^2)	Before or in 2000	1 trial	65	1.10 (-0.15, 2.35)	0.08	N/A	0.02
	After 2000	1 trial	33	-1.65 (-3.48, 0.17)	0.07	N/A	

Outcome	Subgroup	No. of trials	No. of participants	aMD (95% CI)	P for overall effect	P for heterogeneity	P for subgroup interaction
Fat mass index at >3 years (kg/m²)	Before or in 2000	2 trials	183	0.58 (0.01, 1.16)	0.05	0.21	0.03
	After 2000	2 trials	159	-0.36 (-0.97, 0.26)	0.25	0.03	
Percent fat mass in infancy (%)	Before or in 2000	3 trials	150	0.75 (-1.36, 2.86)	0.49	0.58	0.17
	After 2000	5 trials	417	-0.98 (-2.24, 0.29)	0.13	0.005	
Percent fat mass in childhood (%)	Before or in 2000	1 trial	118	1.61 (-2.00, 5.22)	0.38	N/A	0.45
	After 2000	1 trial	126	-0.34 (-3.84, 3.17)	0.85	N/A	
Percent fat mass in adolescence (%)	Before or in 2000	1 trial	65	2.03 (-1.95, 6.02)	0.31	N/A	0.03
	After 2000	1 trial	33	-5.98 (-11.78, -0.18)	0.04	N/A	
Percent fat mass at >3 years (%)	Before or in 2000	2 trials	183	1.78 (-0.96, 4.52)	0.20	0.86	0.13
	After 2000	2 trials	159	-1.31 (-4.25, 1.64)	0.38	0.12	
Lean mass in infancy (kg)	Before or in 2000	3 trials	151	0.32 (0.04, 0.61)	0.03	0.43	0.15
	After 2000	5 trials	418	0.08 (-0.09, 0.25)	0.37	0.0.9	
Lean mass in childhood (kg)	Before or in 2000	1 trial	118	0.01 (-0.95, 0.98)	0.98	N/A	0.36
	After 2000	1 trial	126	-0.62 (-1.56, 0.32)	0.20	N/A	
Lean mass in adolescence (kg)	Before or in 2000	1 trial	65	0.24 (-2.28, 2.77)	0.85	N/A	0.49
	After 2000	1 trial	33	-1.33 (-5.01, 2.35)	0.48	N/A	
Lean mass at >3 years (kg)	Before or in 2000	2 trials	183	0.09 (-0.93, 1.12)	0.86	0.86	0.22
	After 2000	2 trials	159	-0.83 (-1.93, 0.26)	0.14	0.28	
Lean mass index in infancy (kg/m²)	Before or in 2000	3 trials	149	0.11 (-0.36, 0.59)	0.64	0.68	0.63
	After 2000	5 trials	417	0.25 (-0.04, 0.53)	0.09	0.10	
Lean mass index in childhood (kg/m²)	Before or in 2000	1 trial	118	0.01 (-0.53, 0.56)	0.97	N/A	0.35
	After 2000	1 trial	126	-0.35 (-0.88, 0.18)	0.20	N/A	
Lean mass index in adolescence (kg/m²)	Before or in 2000	1 trial	65	0.37 (-0.51, 1.25)	0.41	N/A	0.05
	After 2000	1 trial	33	-1.19 (-2.47, 0.09)	0.07	N/A	
	Before or in 2000	2 trials	183	0.14 (-0.33, 0.60)	0.57	0.56	0.05

Outcome	Subgroup	No. of trials	No. of participants	aMD (95% CI)	P for overall effect	P for heterogeneity	P for subgroup interaction
Lean mass index at >3 years (kg/m²)	After 2000	2 trials	159	-0.55 (-1.04, -0.05)	0.03	0.05	
**Bone development**							
BMC in infancy (g)	Before or in 2000	3 trials	152	21.57 (7.75, 35.39)	0.002	0.69	0.03
	After 2000	3 trials	161	-0.50 (-13.87, 12.88)	0.94	0.49	
BMC in adolescence (g)	Before or in 2000	1 trial	65	-31.23 (-157.39, 94.93)	0.62	N/A	0.92
	After 2000	1 trial	33	-20.18 (-204.06, 163.71)	0.83	N/A	
BMD in infancy	Before or in 2000	3 trials	152	0.013 (0.001, 0.025)	0.04	0.94	0.22
	After 2000	2 trials	115	0.001 (-0.013, 0.015)	0.87	0.59	
BMD in adolescence	Before or in 2000	1 trial	65	-0.02 (-0.054, 0.017)	0.28	N/A	0.36
	After 2000	1 trial	33	0.01 (-0.04, 0.06)	0.70	N/A	

Abbreviation: BMI: body mass index; HC: head circumference; BMC: bone mineral content; BMD: bone mineral density; aMD: adjusted mean difference; N/A: not applicable.
Mean differences were adjusted for sex, gestational age and birthweight z-score

Table S10. Search strategies.

**Embase from 1980 to 2019 April 01**	
#	Search strategies
1	exp prematurity/
2	exp low birth weight/
3	exp small for date infant/
4	exp very low birth weight/
5	(prematur* adj2 infant*).tw.
6	(prematur* adj2 newborn*).tw.
7	(prematur* adj2 neonate*).tw.
8	preterm.tw.
9	low birth weight.tw.
10	low birthweight.tw.
11	VLBW.tw.
12	LBW.tw.
13	ELBW.tw.
14	small for gestation*.tw.

15	SGA.tw.
16	(less than adj6 g).tw.
17	(less than adj3 32 weeks).tw.
18	birth weight below.tw.
19	(gestation* adj2 less than).tw.
20	or/1-19
21	exp breast feeding/
22	exp infant nutrition/
23	exp protein intake/
24	exp dietary supplement/
25	exp omega 3 fatty acid/ct, ad, dt, ig, pa [Clinical Trial, Drug Administration, Drug Therapy, Intragastric Drug Administration, Parenteral Drug Administration]
26	exp arachidonic acid/ae, ct, ad, dt, ig, pa, th [Adverse Drug Reaction, Clinical Trial, Drug Administration, Drug Therapy, Intragastric Drug Administration, Parenteral Drug Administration, Therapy]
27	exp unsaturated fatty acid/ct, dt, pa, th [Clinical Trial, Drug Therapy, Parenteral Drug Administration, Therapy]
28	exp fat intake/ae, ad, dt [Adverse Drug Reaction, Drug Administration, Drug Therapy]
29	exp enteric feeding/
30	exp parenteral nutrition/
31	exp artificial milk/
32	exp breast milk/
33	exp fortified food/
34	exp elemental diet/
35	exp baby food/
36	(breast milk or human milk).tw.
37	formula.tw.
38	PUFA supplement*.tw.
39	feed* regimen*.tw.
40	(protein* adj2 concentration*).tw.
41	probiotic$.tw.
42	parenteral*.tw.
43	enteral*.tw.
44	maternal milk.tw.
45	multinutrient supplement*.tw.
46	(breast fed or breastfed).tw.
47	prebiotic*.tw.
48	diet* supplement*.tw.
49	nutrient enriched.tw.
50	Docosahexaenoic Acid*.tw.
51	arachidonic acid*.tw.
52	(glutamine adj2 supplement*).tw.
53	(taurine adj2 supplement*).tw.

54	(calcium adj2 supplement*).tw.
55	palm olein.tw.
56	palmitic acid.tw.
57	(fortification or fortified).tw.
58	fatty acids.tw.
59	supplement* feed*.tw.
60	complementary feed*.tw.
61	nutrition*.tw.
62	Hydrolysed liquid.tw.
63	Hydrolyzed liquid.tw.
64	gamma-linoleic acid.tw.
65	(diet* adj3 protein*).tw.
66	or/21-65
67	20 and 66
68	Clinical Trial/
69	Randomized Controlled Trial/
70	exp randomization/
71	Single Blind Procedure/
72	Double Blind Procedure/
73	Crossover Procedure/
74	Placebo/
75	Randomi?ed controlled trial$.tw.
76	Rct.tw.
77	random allocation.tw.
78	randomly.tw.
79	randomly allocated.tw.
80	allocated randomly.tw.
81	(allocated adj2 random).tw.
82	Single blind$.tw.
83	Double blind$.tw.
84	((treble or triple) adj blind$).tw.
85	placebo$.tw.
86	prospective study/
87	or/68-86
88	case study/
89	case report.tw.
90	abstract report/ or letter/
91	or/88-90
92	87 not 91
93	67 and 92

Table S11. List of excluded studies

After reading the full texts, we excluded 62 records. The reasons for exclusion and the excluded studies are outlined below.

Reasons	Studies
Wrong intervention	Bai 2005 [1], Beauport 2017 [2], Bernabe-Garcia 2017 [3], Boehm 1993 [4], Boehm 1991 [5], Boehm 1991 [6], Boehm 1993 [7], Boehm 1990 [8], Bora 2017 [9], Carey 1987 [10], Corpeleijn 2016 [11], Costa 1996 [12], Cristofalo 2011 [13], Cristofalo 2013 [14], dos Santos 1997 [15], Faerk 2000 [16], Faerk 2001 [17], Florendo 2006 [18], Gathwala 2008 [19], Hering 1987 [20], Juhl 2018 [21], Lainwala 2017 [22], Lapillonne 1997 [23], Lapillonne 2004 [24], Maggio 2003 [25], McLeod 2010 [26], Mercado 1990 [27], Merritt 1993 [28], Moro 1989 [29], Moro 1991 [30], Nair 2011 [31], Najm 2017 [32], O'Connor 2016 [33], Sankaran 1996 [34], Salas 2018 [35], Schanler 1988 [36], Siripoonya 1989 [37], Sullivan 2009 [38], Sullivan 2010 [39], Tatwavedi 2018 [40], Techasatid 2017 [41], Unger 2016 [42], Vembenil 2007 [43], Willeitner 2017 [44]
Wrong study design	Bier 2000 [45], Brooke 1987 [46], de Klerk 1997 [47], ElSakka 2016 [48], Gemme 1963 [49], Hanmer 1982 [50], Pittaluga 2011 [51], Yesilipek 1992 [52]
Wrong outcomes	Bell 1986 [53], Kulkarni 1984 [54], Lou 2017 [55], Lucas 1984 [56]
Wrong patient population	de Zegher 2012 [57]
Letter/comment	Davies 1992 [58], Embleton 2017 [59]
Unable to locate	Ayutthaya 2006 [60], Misa 1980 [61], Marangione 2009 [62]

References

1. Bai XM, Liu ZJ, Li SJ, Xin P, Li G. Comparison of two parenteral nutrition methods in low birth weight premature infants. [Chinese].

2. Beauport L, Schneider J, Faouzi M, Hagmann P, Huppi PS, Tolsa JF, et al. Impact of early nutritional intake on preterm brain: a magnetic resonance imaging study. Journal of Pediatrics. 2017;181:29-36 e1. doi:10.1016/j.jpeds.2016.09.073

3. Bernabe-Garcia M, Dominguez-Vallejo P, Cruz-Reynoso L, Villavicencio-Torres A, Villegas-Silva R, Inda-Icaza P. Effect of enteral docosahexaenoic acid on retinopathy of prematurity during their hospital stay. FASEB Journal Conference: Experimental Biology. 2017;31(1 Supplement 1).

4. Boehm G, Borte M, Bellstedt K, Moro G, Minoli I. Protein quality of human milk fortifier in low birth weight infants: effects on growth and plasma amino acid profiles. 1993.

5. Boehm G, Borte M, Muller DM, Senger H, Rademacher C. Nutrition of preterm infants with supplemented human milk: EOPROTIN vs human albumin. 1991.

6. Boehm G, Borte M, Muller DM, Senger H, Rademacher C. [Neonatal nutrition with enriched human milk. EOPROTIN 60 in comparison with human albumin]. Kinderarztliche Praxis. 1991;59(10):293-8.

7. Boehm G, Muller DM, Senger H, Borte M, Moro G. Nitrogen and fat balances in very low birth weight infants fed human milk fortified with human milk or bovine milk protein. European Journal of Pediatrics. 1993;152(3):236-9.
8. Boehm G, Senger H, Friedrich M, Muller DM, Beyreiss K. Protein supplementation of human milk for the nutrition of VLBW-infants: human milk protein vs. meat protein hydrolysate. Klinische Padiatrie. 1990;202(5):316-20.
9. Bora R, Murthy NB. In resource limited areas complete enteral feed in stable very low birth weight infants (1000-1500 g) started within 24 h of life can improve nutritional outcome. Journal of Maternal-Fetal and Neonatal Medicine. 2017;30(21):2572-7. doi:http://dx.doi.org/10.1080/14767058.2016.1256992
10. Carey DE, Rowe JC, Goetz CA, Horak E, Clark RM, Goldberg B. Growth and phosphorus metabolism in premature infants fed human milk, fortified human milk, or special premature formula. Use of serum procollagen as a marker of growth. American Journal of Diseases of Children. 1987;141(5):511-5.
11. Corpeleijn WE, de Waard M, Christmann V, van Goudoever JB, Jansen-van der Weide MC, Kooi EM, et al. Effect of Donor Milk on Severe Infections and Mortality in Very Low-Birth-Weight Infants: The Early Nutrition Study Randomized Clinical Trial. JAMA Pediatrics. 2016;170(7):654-61.
12. Costa HP, Kopelman BI, de Almeida AC, Polycarpo AC, Giaccio CD. Growth of premature infants fed own mother's milk supplied with two milk formulas. [Portuguese]. Jornal de Pediatría. 1996;72(3):164-71.
13. Cristofalo EASRJBCLSSTRUK-KSAGDDJRMLL. Exclusive Human Milk vs Preterm Formula: Randomized Trial in Extremely Preterm Infants. 2011.
14. Cristofalo EA, Schanler RJ, Blanco CL, Sullivan S, Trawoeger R, Kiechl-Kohlendorfer U, et al. Randomized Trial of Exclusive Human Milk versus Preterm Formula Diets in Extremely Premature Infants. The Journal of Pediatrics. 2013;163(6):1592-5.e1. doi:10.1016/j.jpeds.2013.07.011
15. dos Santos MM, Martinez FE, Sieber V, Pinhata M, Felin ML. Acceptability and growth of VLBW-infants fed with own mother's milk enriched with a natural or commercial human milk fortifier (HMF). Pediatric Research. 1997:231A.
16. Faerk J, Petersen S, Peitersen B, Michaelsen KF. Diet and bone mineral content at term in premature infants. Pediatric Research. 2000;47(1):148-56.
17. Faerk J, Petersen S, Peitersen B, Michaelsen KF. Diet, growth, and bone mineralization in premature infants. Advances in Experimental Medicine and Biology. 2001;501:479-83.
18. Florendo. A Comparison of Growth in Preterm Infants Fed Two Different Types of Infant Formula Protein. European Journal of Pediatrics. 2006;165.
19. Gathwala G, Shaw C, Shaw P, Yadav S, Sen J. Human milk fortification and gastric emptying in the preterm neonate. International Journal of Clinical Practice. 2008;62(7):1039-43. doi:http://dx.doi.org/10.1111/j.1742-1241.2006.01201.x
20. Hering AE, Vaisman WS, Beca IJP. Evaluation of a modified milk formula in low birth weight neonates. Revista Chilena de Pediatría. 1987;58(3):197.
21. Juhl SM, Ye X, Zhou P, Li Y, Iyore EO, Zhang L, et al. Bovine Colostrum for Preterm Infants in the First Days of Life: A Randomized Controlled Pilot Trial. Journal of Pediatric Gastroenterology and Nutrition. 2018;66(3):471-8. doi:http://dx.doi.org/10.1097/MPG.0000000000001774
22. Lainwala S, Kosyakova N, Spizzoucco AM, Herson V, Brownell EA. Clinical and nutritional outcomes of two liquid human milk fortifiers for premature infants. Journal of Neonatal-Perinatal Medicine. 2017;10(4):393-401. doi:http://dx.doi.org/10.3233/NPM-16164
23. Lapillonne A, Braillon PM, Glorieux FH, Chambon M, Claris O, Delmas PD, et al. Body composition in very low birthweight (VLBW) infants possible influence of the diet? Acta Paediatrica. 1997.

24. Lapillonne A, Salle BL, Glorieux FH, Claris O. Bone mineralization and growth are enhanced in preterm infants fed an isocaloric, nutrient-enriched preterm formula through term. American Journal of Clinical Nutrition. 2004;80(6):1595-603.
25. Maggio L, Sawatzki G, Gallini F, Zuppa A, Vento G, Papacci P, et al. Randomized controlled trial on nutritional efficacy of preterm hydrolyzed formula. Pediatric Research. 2003;54(4):601.
26. McLeod G, Sherriff J, Hartmann PE, Geddes D, Nathan E, Simmer K. Targeting human milk fortification to achieve preterm infant growth targets - A RCT. Journal of Paediatrics and Child Health. 2010;46:13. doi:http://dx.doi.org/10.1111/j.1440-1754.2010.01707.x
27. Mercado M, Yu VY, Gill A. Clinical experience with preterm formulas in very low birthweight infants. Journal of the Singapore Paediatric Society. 1990;32(3-4):137-43.
28. Merritt RJ. Effects of type of dietary protein on acid-base status, protein nutritional status, plasma levels of amino acids, and nutrient balance in the very low birth weight infant RJ COOKE, D WATSON, S WERKMAN, ET AL University of Tennessee, Memphis. Nutrition in Clinical Practice. 1993;8(4):187. doi:10.1177/088453369300800411
29. Moro G, Fulconis F, Minoli I, Pohlandt F, Raiha N. Plasma amino acid differences in VLBW infants fed either protein fortified human milk or a whey-predominant formula. 1989.
30. Moro GE, Minoli I, Fulconis F, Clementi M, Raiha NC. Growth and metabolic responses in low-birth-weight infants fed human milk fortified with human milk protein or with a bovine milk protein preparation. Journal of Pediatric Gastroenterology and Nutrition. 1991;13(2):150-4.
31. Nair JPMJUGNRRCVLS. Early Fortification of Expressed Breast Milk (EBM) Improves Calcium (Ca) and Phosphorus (P) Intake and Reduces Peak Alkaline Phosphatase (AlkP) Level in Premature Neonates. Pediatric Academic Societies Annual Meeting; 2009 May 2 5; Baltimore MD, United States. 2011.
32. Najm S, Lofqvist C, Hellgren G, Engstrom E, Lundgren P, Hard AL, et al. Effects of a lipid emulsion containing fish oil on polyunsaturated fatty acid profiles, growth and morbidities in extremely premature infants: A randomized controlled trial. Clinical Nutrition ESPEN (no pagination), 2017. 2017;Date of Publication: April 03. doi:http://dx.doi.org/10.1016/j.clnesp.2017.04.004
33. O'Connor DL, Gibbins S, Kiss A, Bando N, Brennan-Donnan J, Ng E, et al. Effect of Supplemental Donor Human Milk Compared With Preterm Formula on Neurodevelopment of Very Low-Birth-Weight Infants at 18 Months: A Randomized Clinical Trial. JAMA. 2016;316(18):1897-905.
34. Sankaran K, Papageorgiou A, Ninan A, Sankaran R. A randomized, controlled evaluation of two commercially available human breast milk fortifiers in healthy preterm neonates. Journal of the American Dietetic Association. 1996;96(11):1145-9.
35. Salas AA, Li P, Parks K, Lal CV, Martin CR, Carlo WA. Early progressive feeding in extremely preterm infants: A randomized trial. American Journal of Clinical Nutrition. 2018;107(3):365-70. doi:http://dx.doi.org/10.1093/ajcn/nqy012
36. Schanler RJ, Abrams SA, Garza C. Efforts to provide fortified human milk to very low birthweight infants. American Journal of Perinatology. 1988;5(4):384.
37. Siripoonya P, Sasivimolkul V, Tejavej A, Hotrakitya S, Tontisirin K. Clinical trial of special premature formula for low-birth-weight infants. Journal of the Medical Association of Thailand. 1989;72 Suppl 1:61-5.
38. Sullivan S, Schanler R, Abrams S, Ehrenkranz R, the HSG. A Randomized Controlled Trial of Human Versus Bovine-Based Human Milk Fortifiers in Extremely Preterm Infants. Pediatric Research. 2009.
39. Sullivan S, Schanler RJ, Kim JH, Patel AL, Trawoger R, Kiechl-Kohlendorfer U, et al. An exclusively human milk-based diet is associated with a lower rate of necrotizing enterocolitis than a diet of human milk and bovine milk-based products. Journal of Pediatrics. 2010;156(4):562-7.e1.

40. Tatwavedi D, Nesargi SV, Shankar N, Mathias P, Rao Pn S. Efficacy of modified Tochen's formula for optimum endotracheal tube placement in low birth weight neonates: an RCT. Journal of Perinatology. 2018:1-5. doi:http://dx.doi.org/10.1038/s41372-018-0044-8

41. Techasatid W, Sapsaprang S, Tantiyavarong P, Luvira A. Effectiveness of multicomponent lipid emulsion in preterm infants requiring parenteral nutrition: A two-center, double-blind randomized clinical trial. Journal of the Medical Association of Thailand. 2017;100(9):972-9.

42. Unger S, Gibbins S, Kiss A, Bando N, O'Connor D. Donor milk reduces necrotizing enterocolitis (NEC) but does not improve neurodevelopment of very low birth weight (VLBW) infants at 18 months corrected age. European Journal of Pediatrics. 2016;175(11):1507.

43. Vembenil. The Effects of Enteral Protein Type on Feeding Tolerance and Growth Rate in VLBW Infants. Pediatric Research. 2007.

44. Willeitner A, Anderson M, Lewis J. Highly Concentrated Preterm Formula as an Alternative to Powdered Human Milk Fortifier: A Randomized Controlled Trial. Journal of Pediatric Gastroenterology and Nutrition. 2017;65(5):574-8. doi:http://dx.doi.org/10.1097/MPG.0000000000001638

45. Bier JA, Oliver TL, Ferguson AE, Vohr B. Improved developmental outcomes at one year in very low birth weight infants fed human milk. Paediatric research. 2000;47(4):176a.

46. Brooke OG, Onubogu O, Heath R, Carter ND. Human milk and preterm formula compared for effects on growth and metabolism. Archives of Disease in Childhood. 1987;62(9):917-23.

47. de Klerk A, Schulze KF, Kashyap S, Sahni R, Fifer W, Myers M. Diet and infant behavior. Acta Paediatrica Supplement. 1997;422:65-8.

48. El Sakka A, El Shimi MS, Salama K, Fayez H. Post discharge formula fortification of maternal human milk of very low birth weight preterm infants: An introduction of a feeding protocol in a University Hospital. Pediatric Reports. 2016;8(3):53-8.

49. Gemme G. [Trial of a New Powered Milk, in Premature and Other Infants]. Minerva Dietologica. 1963;18:174-8.

50. Hanmer OJ, Houlsby WT, Thom H, Ross IS, Lloyd DJ, Russell G. Fats as an energy supplement for preterm infants. 1982.

51. Pittaluga E, AR LL, Vernal P, Vega S. Benefits of supplemented preterm formulas on insulin sensitivity and body composition after discharge from the neonatal intensive care unbti. J Pediatrics. 2011;159(6):926-32.

52. Yesilipek MA. Standard and low birth weight formulas compared for effects on growth of preterm infants. Turkish Journal of Pediatrics. 1992;34(1):31-6.

53. Bell A, Halliday H, McClure G, Reid M. Controlled trial of new formulae for feeding low birth weight infants. Early Human Development. 1986;13(1):97-105.

54. Kulkarni PB, Dorand RD, Bridger WM, Payne JH, 3rd, Montiel DC, Hill JG. Rickets in premature infants fed different formulas. Southern Medical Journal. 1984;77(1):13-6.

55. Lou RY. Early minimal breastfeeding combined with assisted intervention to improve feeding intolerance in low-birth-weight preterm infants. [Chinese]. World Chinese Journal of Digestology. 2017;25(34):3080-3. doi:http://dx.doi.org/10.11569/wcjd.v25.i34.3080

56. Lucas A, McLaughlan P, Coombs RR. Latent anaphylactic sensitisation of infants of low birth weight to cows' milk proteins. British Medical Journal Clinical Research Ed. 1984;289(6454):1254-6.

57. de Zegher F, Sebastiani G, Diaz M, Sanchez-Infantes D, Lopez-Bermejo A, Ibanez L. Body composition and circulating high-molecular-weight adiponectin and IGF-I in infants born small for gestational age: breast- versus formula-feeding. Diabetes. 2012;61(8):1969-73. doi:http://dx.doi.org/10.2337/db11-1797

58. Davies DP. Randomized trial of nutrition for preterm infants after discharge. Archives of Disease in Childhood. 1992;67(11):1413-4.
59. Embleton N, Cleminson J. Randomized trial of exclusive human milk versus preterm formula diets in extremely premature infants. Acta Paediatrica, International Journal of Paediatrics. 2017;106(9):1538. doi:http://dx.doi.org/10.1111/apa.13820
60. Ayutthaya JKN. Comparative study between post-discharge formula and standard preterm/term formulon growth and development in premature infants. The 14th Congress of the Federation of Asia-Oceania Perinatal Societies 2006. 2006.
61. Misa S, Yatar A, Modanlou H, Cordano A. Growth patterns and biochemical aspects of very low birthweight (LBW) neonates fed whey proteins based formula, breast milk and a standard premature formula. Pediatric Research. 1980;14(4):506.
62. Marangione P, Introvini P, Castoldi F, Mancuso D, Balestriero M, Lista G. Effect of Different Dietary Protein Intake on Postnatal Growth and Neurodevelopmental Outcome at 1 Year in VLBW Infants. Pediatric Research. 2009.
